# Plasma-Derived Extracellular Vesicle-Enriched Fractions as a Potential Source of Biomarkers for Systemic Sclerosis-Associated Interstitial Lung Disease (SSc-ILD): A Shotgun Proteomic Exploration Analysis †

**DOI:** 10.3390/diagnostics16121879

**Published:** 2026-06-17

**Authors:** Stela Hrkač, Ruđer Novak, Grgur Salai, Václav Pustka, David Potěšil, Zbyněk Zdráhal, Daria Cvetković Kučić, Lovorka Đerek, Joško Mitrović, Lovorka Grgurević

**Affiliations:** 1 Department of Clinical Immunology, Allergology and Rheumatology, University Hospital Dubrava, 10 000 Zagreb, Croatiajmitrovi@kbd.hr (J.M.); 2 Department of Proteomics, Center for Translational and Clinical Research, School of Medicine, University of Zagreb, 10 000 Zagreb, Croatia; 3 BIMIS—Biomedical Research Center Šalata, School of Medicine, University of Zagreb, 10 000 Zagreb, Croatia; 4 Department of Pulmonology, University Hospital Dubrava, 10 000 Zagreb, Croatia; salai.grgur@gmail.com; 5 Central European Institute of Technology, Masaryk University, 612 00 Brno, Czech Republiczdrahal@sci.muni.cz (Z.Z.); 6 Department of Cardiothoracic Radiology, University Hospital Centre Zagreb, 10 000 Zagreb, Croatia; 7 Clinical Department for Laboratory Diagnostics, University Hospital Dubrava, 10 000 Zagreb, Croatia; 8 School of Medicine, Catholic University of Croatia, 10 000 Zagreb, Croatia; 9 School of Medicine, University of Zagreb, 10 000 Zagreb, Croatia; 10 Faculty of Pharmacy and Biochemistry, University of Zagreb, 10 000 Zagreb, Croatia; 11 Department of Anatomy, “Drago Perović”, School of Medicine, University of Zagreb, 10 000 Zagreb, Croatia

**Keywords:** systemic sclerosis, interstitial lung disease, extracellular vesicles, proteomics, SP-B, Cav-1, Siglec-5, extracellular matrix

## Abstract

**Background:** Systemic sclerosis (SSc)-associated interstitial lung disease (SSc-ILD) is the leading cause of morbidity and mortality in patients with SSc, with an unmet need for validated, minimally invasive biomarkers for early detection. Extracellular vesicles (EVs) present underexplored pathogenic players and potential biomarker sources in SSc-ILD. We performed a proteomic shotgun study aiming to identify disease-specific protein signatures and potential biomarker candidates. **Methods:** The study included 30 SSc patients divided into SSc-ILD and SSc w/o ILD groups and 20 matched controls. Plasma-derived EV-enriched fractions were analysed using liquid chromatography–mass spectrometry. Bioinformatic analysis, including differentially expressed proteins (DEPs), functional enrichment, protein–protein interaction network and Markov Cluster (MCL) analysis was performed. **Results:** Analysis of DEPs showed 14 significantly upregulated and 1 downregulated protein when comparing the SSc-ILD to the SSc w/o ILD group, 222 upregulated and 257 downregulated proteins between the SSc-ILD and control group, as well as 362 upregulated and 492 downregulated proteins between the SSc w/o ILD and control group. Functional enrichment analysis and MCL analysis pointed to disease-specific processes of extracellular matrix (ECM) and immune dysregulation, which largely overlapped between SSc-ILD and SSc w/o ILD groups. Among identified DEPs, SP-B, Cav-1 and Siglec-5 emerged as potential candidate biomarkers for SSc-ILD. **Conclusions:** Proteomic analysis of plasma-derived EV-enriched fractions shows potential EV involvement in pathogenic SSc processes, mainly related to ECM and immune dysregulation, as well as potential candidate biomarkers for SSc-ILD. Further studies are required to validate these results and assess biomarker potential and translational applicability of identified proteins.

## 1. Introduction

Systemic sclerosis (SSc) or scleroderma is a complex autoimmune disease characterized by the development of cutaneous and tissue fibrosis, as well as fibroproliferative vascular changes [1,2]. It more commonly affects women, with a women-to-men ratio of almost 5:1 [3]. SSc is associated with a substantial mortality and disease burden, which is closely linked to its various internal organ manifestations. Among these manifestations SSc-associated interstitial lung disease (SSc-ILD) is one of the leading causes of morbidity and mortality in patients with SSc [1,4,5]. It usually presents with common, non-specific symptoms such as dyspnoea and cough, while a non-specific interstitial pneumonia (NSIP) pattern with ground glass opacities, fine reticulations, and usually with subpleural sparing is most commonly observed as an underlying pattern on high-resolution computed tomography (HRCT) scans [6]. Up to one third of patients present with a typical interstitial pneumonia (UIP) pattern, characterized on HRCT by subpleural basal predominance, reticulations, honeycombing, often traction bronchiectasis, and the absence of features suggesting alternative diagnosis [7]. HRCT remains a key step for establishing the diagnosis of SSc-ILD, however its use is often burdened by restricted access, lack of standardised follow-up guidelines, and the need to rationalize patient radiation exposure. Despite its clinical importance, there are still no validated, minimally invasive biomarkers for screening and early detection, disease stratification, or monitoring of SSc-ILD [5]. There is also a need for better understanding of the complex interplay of fibroblasts, epithelial and immune cells that regulate pulmonary fibrosis in SSc-ILD. The immune, epithelial and vascular dysfunction result in dysregulated fibroblast activation and increased extracellular matrix (ECM) deposition [8,9]. Emerging evidence suggests that the communication between these cells might be mediated by extracellular vesicles (EVs), thereby promoting vascular damage and progressive fibrosis of lung tissue [10].

EVs are lipid bilayer membrane-delimited, nano- to micro-sized particles, which are categorised into apoptotic bodies, microparticles, and exosomes [11,12]. They appear to be released by all cell types and are therefore isolated from different biological fluids. As EVs play a crucial role in intercellular communication by transferring different functionally active molecules, they serve as valuable diagnostic and prognostic biomarkers for a variety of conditions, including SSc-ILD [5,10,13,14,15]. Some studies have shown that a general increase in EV levels is associated with SSc-ILD, while expression of specific EV surface markers is linked to SSc-ILD progression, endothelial damage, and vascular complications [12,15,16]. Supporting their functional relevance, EVs isolated from SSc patients stimulate ECM production by fibroblasts in vitro [17]. Additionally, EVs have been implicated in vasculopathy, coagulation, initiation, and amplification of inflammation, as well as propagation of lung fibrosis, i.e., processes important for SSc-ILD pathogenesis [15,18]. Mesenchymal stem cell-derived EVs have also been proposed as therapeutic options to target these processes and ameliorate SSc symptoms [19,20].

The potential use of EVs to complement the process of diagnosis and follow-up is minimally invasive and offers clear advantages to tissue biopsies or bronchoscopy-obtained samples [4]. However, EV research in SSc-ILD is limited and inconsistent, as most studies focus on quantification rather than on the functional characterization that would provide relevant insight into the roles of EVs in the disease mechanism [5,10,21]. In this study, we performed a shotgun proteomic analysis of plasma-derived EV-enriched fractions in patients with SSc-ILD, SSc without ILD, and matched controls, with the aim of identifying disease-specific protein signatures and potential biomarker candidates.

## 2. Materials and Methods

This cross-sectional, observational study included patients with SSc (*N* = 30), recruited from the Department of Clinical Immunology, Allergology and Rheumatology, University Hospital Dubrava, as well as age- and sex-matched healthy controls (*N* = 20). All patients fulfilled the classification criteria for SSc by the 2013 American College of Rheumatology (ACR)/European League Against Rheumatism (EULAR) [22]. Clinical data from the SSc patients were collected, as well as most recent forced vital capacity (FVC) and diffusing capacity of the lungs for carbon monoxide (DLco) values. Most recent chest HRCT scans were analysed by an experienced radiologist to evaluate and classify ILD presence and extent using Goh score, which classifies ILD into limited (if <20% disease extent on HRCT), and extensive (if >20% disease extent on HRCT). For cases in which the distinction between more or less than 20% is unclear—“indeterminate” cases—FVC values of <70% and ≥70% were used to classify the disease into extensive and limited, respectively [23].

Subjects were divided into three groups: SSc patients with identified SSc-ILD (SSc-ILD group, *N* = 12), SSc patients without identified ILD (SSc w/o ILD group, *N* = 18), and a healthy control group (CTRL, *N* = 20). Exclusion criteria were active malignant disease (cancer), clinical and laboratory signs of acute infection, and an operative procedure in the last 14 days. Baseline demographic characteristics and clinical variables were compared among the three groups using the chi-square test or Fisher’s exact test for categorical variables, and one-way analysis of variance (ANOVA) or the Kruskal–Wallis test for continuous variables, as appropriate according to data distribution. Statistical analyses for demographic and clinical variables were performed using jamovi software, version 2.6.26.

### 2.1. Sample Collection, Plasma-Derived EV-Enriched Fraction Isolation and EV Lysis

Blood samples were obtained from the study participants by venepuncture into vacuettes (containing 3.8% sodium citrate) and centrifuged at 3000 *g* for 15 min to obtain platelet-poor plasma, aliquoted and stored at −80 °C until further analysis. To isolate plasma-derived EV-enriched fractions, 1.1 mL of plasma from each participant was further centrifuged at 21,000 *g* for 35 min at 4 °C. Following a washing step in phosphate-buffered saline (PBS), the obtained EV pellet was resuspended and lysed with 0.1% *n*-dodecyl β-D-maltoside (DDM) in 50 mM ammonium bicarbonate. To aid protein release, 0.01 M dithiotreitol (DTT) was added, the solution was briefly sonicated and incubated for 15 min at 95 °C.

### 2.2. Protein Sample Preparation

Protein samples were alkylated by the addition of 0.04 M iodoacetamide (IAA) which was quenched after 20 min using 0.01 M DTT. Proteins were digested with 250 ng of liquid chromatography–mass spectrometry (LC-MS)-grade trypsin overnight at 37 °C, and digestion was stopped by adding acetic acid. The resulting supernatants were purified using in-house made C18 StageTips [24]. Peptide samples were loaded onto the StageTips, washed with 0.1% formic acid, and stored at 4 °C until liquid chromatography–tandem mass spectrometry (LC-MS/MS) analysis was undertaken.

### 2.3. LC-MS/MS Analysis and Data Processing

Peptides were extracted into LC-MS vials using 80% acetonitrile (ACN) with 20% 0.1% formic acid. Peptide samples were analysed using a nano-flow liquid chromatography system (RSLCnano, Thermo Scientific, Waltham, MA, USA) coupled online to a timsTOF Ultra 2 mass spectrometer (Bruker, Billerica, MA, USA). A 60 min linear LC gradient was used. MS and MS/MS spectra were acquired in a time-of-flight analyser using data-independent acquisition (DIA) mode with trapped ion mobility spectrometry (TIMS), covering a precursor *m*/*z* range of 400–1000. Each peptide mixture was analysed separately. For all samples, 50 ng of peptides were injected per analysis based on concentrations determined during peptide quality control; for samples with limited material, approximately half of the final peptide mixture was used when 50 ng was not available. DIA data were processed using DIA-NN software (version 2.1.0) in library-free search mode. Protein identification was performed against a combined database consisting of an indexed retention time(iRT)/trypsin database (version 241015; 12 protein sequences containing single iRT peptides and one additional trypsin sequence) and the UniProtKB Human reference proteome (taxonomy *Homo sapiens*, taxon ID 9606; version 2025-04-24; 20,647 protein sequences). Match between runs (MBR) was enabled across the entire dataset. False discovery rates were controlled at 1% at both the precursor and protein group levels. Search parameters were optimized based on a preliminary analysis of a subset of the data, after which algorithm parameters were set to automatic optimization and the “unrelated runs” option was enabled. Protein groups were inferred using the default DIA-NN protein inference algorithm based exclusively on proteotypic peptides. During further data processing precursors were filtered for those being quantified in 50% of replicates in at least one sample group. Filtered precursor intensities (raw precursor intensities normalized internally by DIA-NN) were further normalized using loessF function. Filtered and normalized precursor intensities were imputed using global quantile (0.001) value. Filtered, normalized and imputed precursor intensities were used to calculate max label-free quantification (MaxLFQ) protein level intensities using the iq R package (4.2.0). Filtered and normalized precursor intensities were used to calculate DIA-total protein approach (TPA) protein level intensities useable as absolute protein abundance estimates. MaxLFQ-derived protein intensities were subsequently used for statistical analysis using the LIMMA framework. Potential outliers were identified with cluster analysis (using Pearson pairwise correlation coefficients) as well as protein group intensities correlation scatterplots and were excluded from further statistical analysis.

### 2.4. Additional Data Analysis

Study groups were compared using normalized values and imputed data, statistical evaluation was done with a moderated *t*-test using LIMMA package in R. *p* values were adjusted on multiple hypothesis testing using the Benjamini and Hochberg method for multiple hypothesis testing. Proteins with an adjusted *p*-value ≤ 0.05 and a fold change (FC) ≥ 2 (for upregulated) or FC ≤ 0.5 (for downregulated) were considered statistically significant differentially expressed proteins (DEPs). Data were visualized in the in-house built software Proteo Visualizer 3.0.8 (Cupak, M. Proteo-Visualizer 2026 (CEITEC, Brno, Czech Republic)). Top DEPs were identified by highest fold change values.

Functional enrichment analysis, i.e., overrepresentation analysis (ORA), was performed separately on upregulated and downregulated DEPs using ShinyGO 0.85.1 and its enrichment function with all identified proteins as the background, analysing the pathway databases “GO Molecular Function”, “GO Biological Process”, “GO Cellular Component” and “KEGG”. Terms were considered significant at false discovery rate (FDR) < 0.05. The top 20 significant enriched terms in each category (among terms with significant FDR) were selected for further interpretation by highest fold enrichment values. This analysis was not performed on the SSc-ILD vs. SSc contrast due to the low number of identified DEPs.

**Figure 1:**
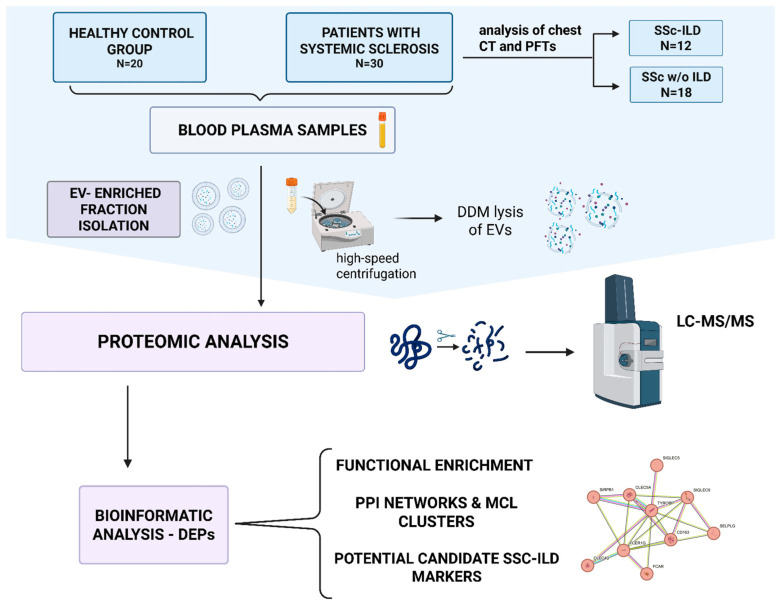
Study outline depicting a combined overview of study groups, the experimental workflow and subsequent analysis of results. The study included two groups of systemic sclerosis (SSc) patients: an (SSc)-associated interstitial lung disease (SSc-ILD) group (*N* = 12) and an SSc w/o ILD group (*N* = 18), as well as a matched control group (*N* = 20). Extracellular vesicle (EV)-enriched fractions were isolated from blood plasma using high-speed centrifugation and the EVs were lysed using *n*-dodecyl β-D-maltoside (DDM) to release protein content. Proteomic analysis was performed using liquid chromatography–tandem mass spectrometry (LC-MS/MS), after which differentially expressed proteins (DEPs) were identified, followed by data analysis including functional enrichment analysis, protein–protein interaction (PPI) network and Markov cluster (MCL) analysis. Created in BioRender. Hrkač, S. (2026) https://BioRender.com/a6vpaje.

Analysis and visualization of protein–protein interactions (PPIs) between sets of DEPs, as well as Markov cluster (MCL) algorithm analysis of clusters (in the PPI networks) was done using STRING (version 12.0) for the SSc-ILD vs. CTRL contrast. This was not performed on the SSc-ILD vs. SSc contrast due to the limited number of identified DEPs, nor on the SSc w/o ILD vs. CTRL contrast to avoid redundancy. The study outline is depicted in Figure 1.

Additional analyses were performed on the three top DEPs (by highest fold change values) from the SSc-ILD vs. SSc w/o ILD contrast to evaluate biomarker potential, namely receiver operating characteristic (ROC) curve analysis was undertaken and a correlation analysis matrix was plotted (which included the top three DEPs, Goh score, pulmonary function tests, presence of SSc-ILD, and immunosuppresive therapy). Additional analyses were performed by employing the jamovi software (2024; Version 2.6) with an additional package DiagROC 0.9.4. Goh score was stratified as 0—no ILD, 1—limited, 2—extensive. DLco and FVC values were used as %predicted. MAXLFQ (KNIME normalized) data were employed as a surrogate measure of protein abundance within each sample, data normality was formally tested with the Shapiro–Wilks test. Correlation analysis was performed by plotting a correlation matrix with Spearman’s correlation due to the fact that the data were non-parametric. Additional ROC analysis (for distinguishing between SSc-ILD and SSc w/o ILD) was performed and optimal cut-off values were found based on the Youden’s Y index. In order to potentially find the best biomarker candidate, ROC curves were mutually compared with the DeLong’s test.

## 3. Results

### 3.1. Subject Clinical Characteristics

According to chest HRCT analysis and scoring according to Goh et al., among the 30 SSc subjects, 12 had SSc-ILD (SSc-ILD group), while 18 did not (SSc w/o ILD group). Among the 12 subjects with verified SSc-ILD, 3 had extensive SSc-ILD. Due to the limited number of patients with extensive SSc-ILD, for further analysis we formed a single SSc-ILD group (Table 1). The subjects were predominantly female, which is in line with existing epidemiological data on prevalence according to sex [3].

As expected, subjects with SSc-ILD had significantly lower values of pulmonary function tests (PFTs)—namely FVC (*p* < 0.001) and DLco (*p* = 0.015)—compared with the SSc w/o ILD group (Table 2.) Additional clinical data from the SSc subjects are shown in Table 2.

**Table 1:** Basic group characteristics.

	SSc-ILD	SSc w/o ILD	CTRL	*p*-Value
Number (*N*)	12	18	20	/
Age (median [Q1–Q3])	62 [54.75–66.25]	58 [50.25–68]	58.5 [50–60.5]	0.368
Female (*N*)	10	17	19	0.536
Male (*N*)	2	1	1	
Current smokers (*N*)	1	2	2	0.338
Previous smokers (*N*)	5	4	2	
Never-smokers (*N*)	6	12	16	
Immunosuppressive therapy (*N*)	9	9	0	<0.001

*N*—number, [Q1–Q3]—interquartile range.

**Table 2:** Additional clinical data on the SSc subjects.

	SSc-ILD	SSc w/o ILD	*p*-Value
FVC, % (mean ± SD)	82.3 ± 15.7	108 ± 17.7	<0.001
DLco, % (mean ± SD)	51.3 ± 18.9	73.6 ± 22	0.015
Limited ILD *	9	0	/
Extensive ILD *	3	0	/
PAH (*N*)	6	3	0.102
Raynaud’s phenomenon (*N*)	11	16	1
Digital ulcers (*N*)	5	6	0.643
Anticentromere antibody (*N*)	0	14	<0.001
Anti-Scl-70 antibody (*N*)	7	1	0.003
Anti-Ro52 antibody (*N*)	1	2	1
Anti-RNA Polymerase III antibody (*N*)	0	0	/
MTX treatment (*N*)	3	4	1
Mycophenolate treatment (*N*)	2	1	0.548
Azathioprine treatment	1	0	0.4
Cyclophosphamide treatment (*N*)	1	0	0.4
Antimalarial treatment (*N*)	1	2	1
Antifibrotic treatment (*N*)	1	0	0.4
Glucocorticoid treatment (*N*)	7	6	0.176

* according to Goh score. *N*—number, SD—standard deviation, FVC—forced vital capacity, DLco—diffusing capacity of the lungs for carbon monoxide, PAH—pulmonary arterial hypertension, MTX—methotrexate.

### 3.2. LC-MS/MS Data Evaluation

**Figure 2:**
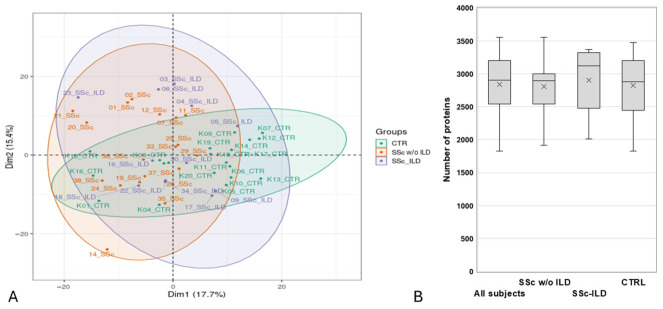
(**A**) Diagram showing principal component analysis (PCA) for the three groups, where each point on the graph corresponds to one subject. (**B**) Graph with box plots showing comparable numbers of identified proteins per subject across sample groups.

The total number of proteins identified in all sample groups was 3685. Principal component analysis (PCA) of the 500 most variable EV-enriched fraction proteins showed partial separation between the CTRL group and SSc samples, while SSc w/o ILD and SSc-ILD exhibited substantial overlap, as shown in Figure 2A.

Across all sample groups, the median [Q1–Q3] number of identified proteins per sample was 2903.5 [2588.75–3191] (with a range of 1824–3558). The median number of identified proteins per sample was comparable across groups: CTRL 2877 [2623–3199], SSc w/o ILD 2899 [2601–2960], and SSc-ILD 3120 [2566–3293], as shown in Figure 2B.

**Figure 3:**
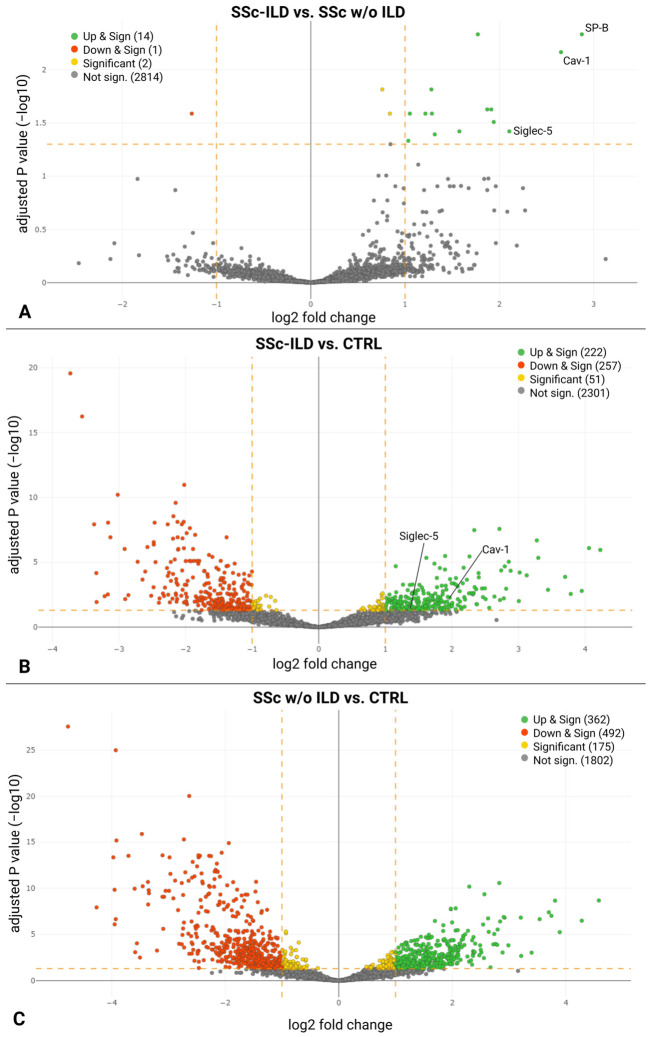
(**A**–**C**) Volcano plots showing the upregulated and downregulated differentially expressed proteins (DEPs) among the group comparisons: SSc-ILD vs. SSc w/o ILD (**A**), SSc-ILD vs. CTRL (**B**), and SSc w/o ILD vs. CTRL (**C**). The yellow dashed lines on the graph indicate thresholds: first, *p*-value ≤ 0.05 (values adjustment on multiple hypothesis testing using the Benjamini and Hochberg method, −log10-transformed) and, second, log2-transformed fold change higher than 1, or lower than −1. Up- and downregulated proteins that meet both thresholds are shown in green and red dots, respectively. The yellow dots represent proteins that did not meet the second threshold. Created with Proteo Visualizer 3.0.8 and BioRender. Hrkač, S. (2026) https://BioRender.com/kas8b06.

When comparing the SSc-ILD group with the SSc w/o ILD group, we identified 14 significantly upregulated DEPs and 1 downregulated DEP (Figure 3A). The upregulated DEPs are listed in Table 3. Among the upregulated DEPs, the three most overabundant proteins were pulmonary surfactant-associated protein B (SP-B) (FC = 7.34, *p* = 0.005), caveolin-1 (Cav-1) (FC = 6.297, *p* = 0.007), and sialic acid-binding Ig-like lectin 5 (Siglec-5) (FC = 4.3, *p* = 0.038). The only downregulated DEP in this contrast was dipeptidase 2 (FC = 0.417, *p* = 0.026).

Between the SSc-ILD and CTRL group, we identified 222 upregulated and 257 downregulated DEPs (Figure 3B). The top 20 upregulated and downregulated DEPs (sorted by fold change) are shown in Table 4 (full lists of DEPs in Supplementary Tables S1 and S2). Interestingly, among the upregulated DEPs we also identified caveolin-1 (FC = 3.91, *p* = 0.005) and siglec-5 (FC = 2.864, *p* = 0.032), which were among the top DEPs in the SSc-ILD vs. SSc contrast. Although SP-B had a substantial fold change in this contrast, it did not reach statistical significance (FC = 2.41, *p* = 0.104). Notably, dipeptidase 2 (which was also downregulated in SSc-ILD vs. SSc) was also identified as significantly downregulated in the SSc-ILD vs. CTRL contrast (FC = 0.23, *p* = 1.191 × 10^−7^).

Comparison of the SSc w/o ILD group with the CTRL group yielded 362 upregulated and 492 downregulated DEPs (Figure 3C). The top 20 upregulated and downregulated DEPs (sorted by fold change) are shown in Table 5 (full list of DEPs visible in Supplementary Tables S3 and S4).

Interestingly, none of the identified upregulated DEPs in the SSc-ILD vs. SSc w/o ILD were identified in this contrast, while there were 155 common upregulated DEPs with the SSc-ILD vs. CTRL contrast, indicating potential SSc-ILD specificity of some of the identified DEPs in the SSc-ILD contrasts. Volcano plots showing the identified DEPs across all the group comparisons can be seen in Figure 3A–C.

### 3.3. Functional Enrichment and Protein–Protein Interaction (PPI) Network Analysis

**Figure 4:**
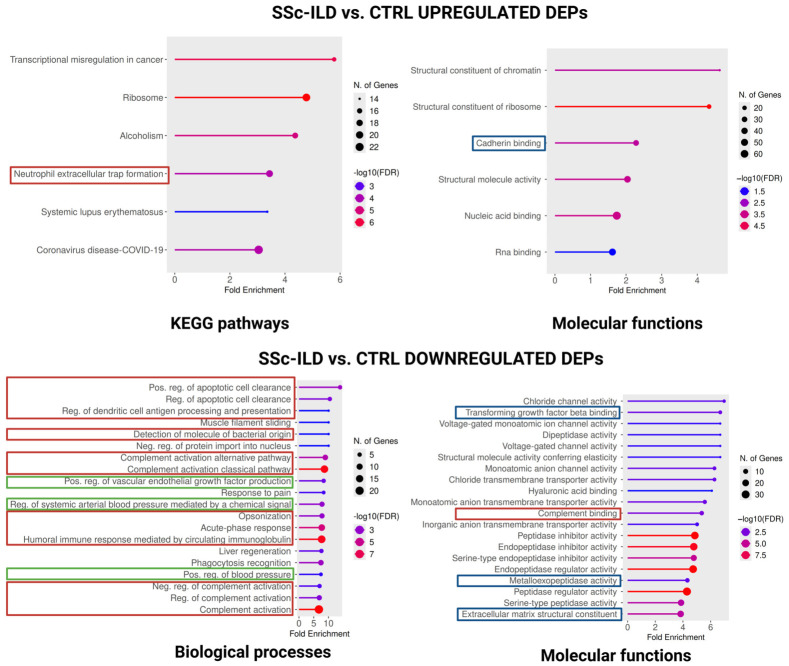
Upregulated and downregulated differentially expressed proteins (DEPs) (SSc-ILD vs. CTRL contrast) are associated with enriched processes and functions depicted in the panels. Enriched terms are marked by coloured boxes according to potential functional and pathophysiological significance: extracellular matrix (ECM) dysregulation/fibrosis (blue), immune dysregulation (red) and vascular dysfunction (green). Created with ShinyGO 0.85.1 and BioRender. Hrkač, S. (2026) https://BioRender.com/e2ar90d.

**Figure 5:**
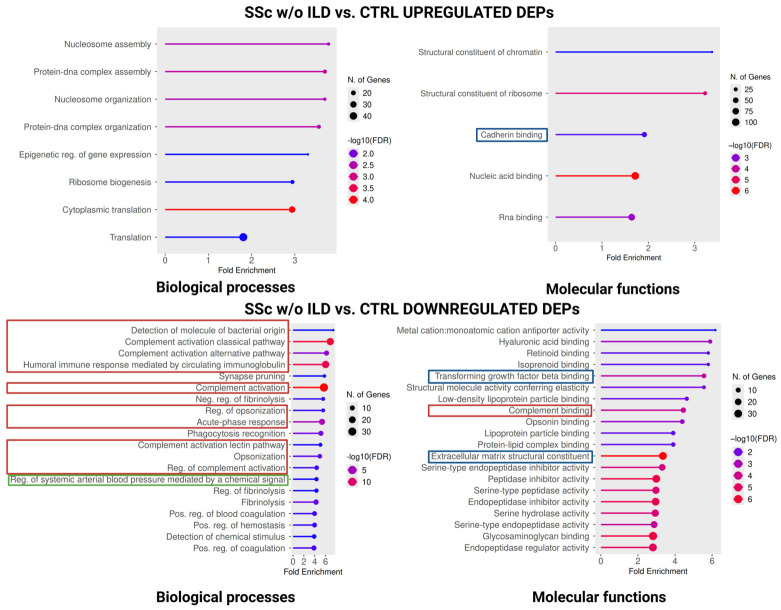
Upregulated and downregulated differentially expressed proteins (DEPs) (SSc w/o ILD vs. CTRL contrast) are associated with enriched processes and functions depicted in the panels. Enriched terms are marked by coloured boxes according to potential functional and pathophysiological significance: extracellular matrix (ECM) dysregulation/fibrosis (blue), immune dysregulation (red), and vascular dysfunction (green). Created with ShinyGO 0.85.1. and BioRender. Hrkač, S. (2026) https://BioRender.com/idqeobj.

For the upregulated DEPs in the SSc-ILD vs. CTRL contrast, functional enrichment analysis revealed neutrophil extracellular trap (NET) formation and cadherin binding (Figure 4). The enrichment of NET formation-related pathways was supported by upregulation of proteins such as S100A12, complement components (C5, C8γ), and lipopolysaccharide-binding protein (LBP), indicating neutrophil-driven inflammatory processes. Enrichment analysis of downregulated DEPs from the SSc-ILD vs. CTRL contrast showed enriched terms related to regulation of apoptotic cell clearance, as well as processes and functions related to immune dysregulation (Figure 4). Processes related to arterial blood pressure regulation and vascular endothelial growth factor production are supported by downregulated proteins, such as endothelial protein C receptor. Several enriched terms, possibly related to ECM dysfunction were also identified (Figure 4), which was supported by differential expression of proteins involved in matrix organization and remodelling, including fibulin-5, ECM metalloproteinase inducer, fibronectin and lumican, suggesting altered ECM homeostasis. Functional enrichment analysis of SSc w/o ILD vs. CTRL contrast, revealed pathways largely overlapping with those observed in the SSc-ILD group, including enriched terms related to immune and ECM dysregulation (Figure 5). These shared patterns suggest that many of the observed proteomic alterations are driven by systemic sclerosis itself rather than ILD-specific mechanisms. However, in the SSc-ILD contrast there seem to be a larger number of relevant enriched terms, particularly those related to ECM dysregulation. Notably, certain pathways, such as NET formation and apoptosis-related pathways, appeared only in the SSc-ILD group, indicating their potential SSc-ILD specificity. The top 20 enriched terms for all analysed pathways can be found in Supplementary Tables S5–S8.

**Figure 6:**
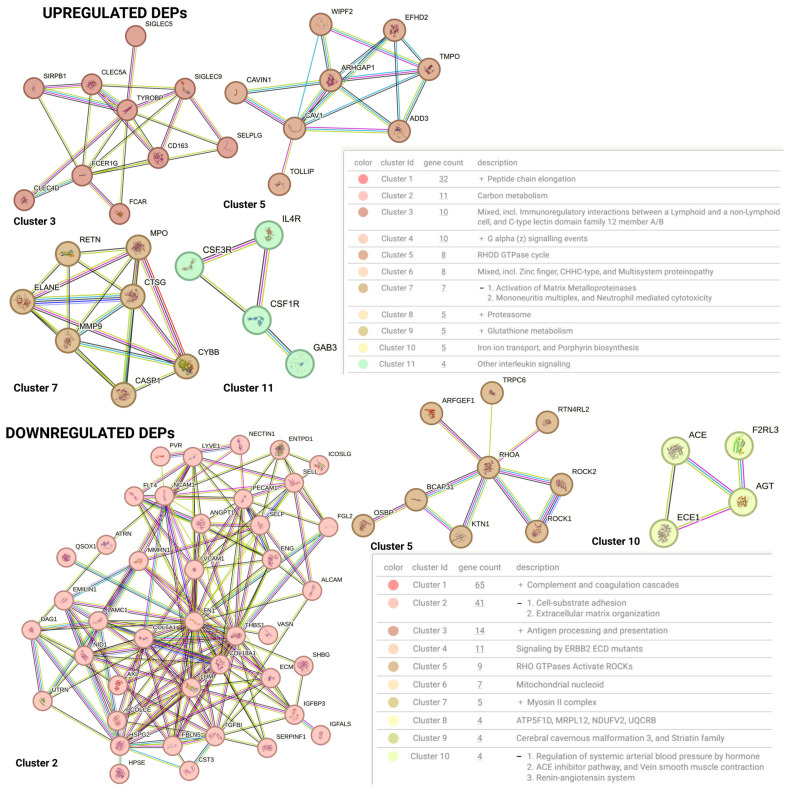
In the upper and lower panel selected protein–protein interaction networks from relevant functional clusters are shown, obtained by Markov cluster (MCL) analysis of differentially expressed proteins (DEPs) from the SSc-ILD vs. CTRL contrast, as well as results from the MCL analysis in table form, showing cluster names and corresponding colour. The upper panel shows selected clusters from upregulated DEPs: the immunoregulatory interaction cluster, the RhoDGTPase cluster, activation of matrix metalloproteinases, and the interleukin signalling cluster. The lower panel shows selected clusters from downregulated DEPs: extracellular matrix organization cluster, Rho GTPase cluster, and regulation of systemic arterial blood pressure cluster. Created using STRING (version 12.0) and BioRender. Hrkač, S. (2026) https://BioRender.com/eoxti3l.

Analysis of PPI networks using STRING for the SSc-ILD vs. CTRL contrast showed densely interconnected protein networks for both upregulated and downregulated DEPs (Supplementary Figures S1 and S2). MCL analysis for upregulated DEPs revealed clusters related to immune regulation and interleukin signalling, Rho GTPase signalling and matrix metalloproteinase activation (Figure 6). For the downregulated DEPs in this contrast, MCL cluster analysis showed clusters related to ECM organization, Rho GTPases, and to the regulation of systemic arterial blood pressure (Figure 6). This points to the pathogenic mechanisms also reflected through enrichment analysis—namely, ECM dysfunction and immune dysregulation, as well as regulation of arterial blood pressure.

### 3.4. ROC Curve and Correlation Analysis for SP-B, Cav-1 and Siglec-5

**Figure 7:**
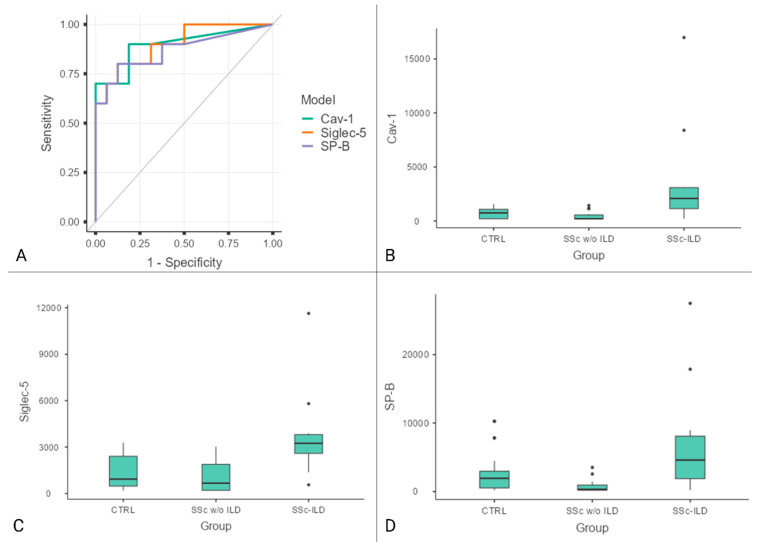
(**A**) Graph showing combined receiver operating characteristic (ROC) curves (x-axis—1-specificity, y-axis—sensitivity) for the three analysed candidate biomarkers—Cav-1 (green), Siglec-5 (orange) and SP-B (light purple). ROC analysis for Siglec-5 yielded an area under the curve (AUC) of 0.9 (95% CI: 0.777–1.000), *p* < 0.001;/ for Cav-1 an AUC of 0.897 (95% CI: 0.758–1.000), *p* < 0.001; and for SP-B the AUC was 0.869 (95% CI 0.707–1), *p* < 0.001. (**B**–**D**) Box plots showing distribution of expression (MAXLFQ, KNIME normalized values) of the biomarker candidate proteins Cav-1, Siglec-5 and SP-B, respectively, across the three subject groups: controls (CTRL), SSc w/o ILD, and SSc-ILD. Created in BioRender. Hrkač, S. (2026) https://BioRender.com/gvz53ji.

The top three DEPs in the SSc-ILD vs. SSc w/o ILD contrast, proteins SP-B, Cav-1 and Siglec-5, were additionally evaluated to determine their biomarker potential for SSc-ILD. All three proteins showed higher expression values in the SSc-ILD group, compared with the SSc w/o ILD group and control group, when visually compared by box plots (Figure 7B–D). However, this difference is much less pronounced when both SSc groups are combined and compared with the control group (Supplementary Figures S3–S5).

ROC curve analysis for Siglec-5 yielded an area under the curve (AUC) of 0.9 (95% CI: 0.777–1.000), *p* < 0.001. With an optimal cut-off (2268.1765), sensitivity was 80% (95% CI: 44.39–97.48%) and specificity 87.5% (95% CI: 61.65–98.45%).

ROC analysis for Cav-1 yielded an AUC of 0.897 (95% CI: 0.758–1.000), *p* < 0.001. With an optimal cut-off (687.0742), sensitivity was 90% (95% CI: 55.5–99.75%) and specificity 81.25% (95% CI: 54.35–95.95%).

ROC analysis for SP-B yielded an AUC of 0.869 (95% CI 0.707–1), *p* < 0.001. With an optimal-cut off (1544.59), sensitivity was 80% (95% CI 44.39–97.48) and specificity 87.5% (95% CI 61.65–98.45%).

Combined ROC curves for the proteins are shown in Figure 7, panel A. ROC curves were mutually compared with the DeLong’s test where no statistically significant difference was found among them (Supplementary Table S9).

Correlation matrix analysis (Supplementary Table S10) found that Cav-1 and Goh score are positively correlated (Spearman’s rho = 0.71, *p* = <0.001). SP-B is negatively correlated with DLco (%pred) (Spearman’s rho = −0.484, *p* = 0.019) and positively correlated with Goh score (Spearman’s rho = 0.639, *p* < 0.001). Siglec-5 is negatively correlated with FVC (%pred) (Spearman’s rho = −0.543, *p* = 0.005) and positively correlated with Goh score (Spearman’s rho = 0.694, *p* < 0.001). Interestingly, Cav-1 and SP-B were also positively correlated with SP-B (Spearman’s rho 0.417, *p* = 0.005) and Siglec-5 (Spearman’s rho = 0.453, *p* = 0.002). No significant correlation between the abundance levels of these proteins and immunosuppression was detected. There was significant correlation for the presence of SSc-ILD for all three proteins (Supplementary Table S10).

## 4. Discussion

In this study, we identified distinct proteomic signatures of plasma-derived EV-enriched fractions in SSc patients, characterized by immune and ECM dysregulation, as well as differentially expressed proteins, when comparing SSc patients with and without lung involvement, among which, we propose potential SSc-ILD candidate biomarkers.

Namely, fibrosis is a hallmark of SSc, with lung involvement being the leading cause of death in these patients [18]. EVs contribute to ECM deposition, propagation of lung fibrosis and immune modulation, supporting their role in SSc-ILD pathophysiology [5,10,18,19,20,25]. We compared subject groups with SSc-ILD and SSc without ILD to matched controls. Our analysis showed no significant differences in age, sex and smoking status among the three groups. The SSc-ILD and SSc w/o ILD group were comparable in pulmonary artery hypertension (PAH), digital ulcerations, Raynaud’s phenomenon, and immunosuppressive therapy. Lower values of pulmonary function tests in the SSc-ILD group are expected, as are the observed differences in antibody profiles. More frequent presence of anti-Scl-70 antibodies in SSc-ILD patients is expected, and anticentromere antibodies align with lower ILD risk [7,26,27].

Our results show that proteomic signatures of plasma-derived EV-enriched fractions from the SSc w/o ILD and SSc-ILD groups largely overlap and differ substantially from the control group. A deeper insight revealed differences in proteins associated with ECM dysregulation, NET formation, and regulation of apoptosis in the SSc-ILD groups, indicating potential specific SSc-ILD mechanisms [8,28].

Transforming growth factor beta (TGF-β) is a central mediator in SSc pathogenesis and SSc-related tissue fibrosis [29,30]. Dysregulation of ECM production is reflected through enriched molecular functions of TGF-β binding among downregulated DEPs. It might also be reflected by enriched cadherin binding, as altered cadherin expression has been linked to epithelial–mesenchymal transition (EMT)-dependent fibrosis in SSc [31,32]. Because cadherins have an important role in sensing ECM stiffness, fibrosis-associated stiffness may contribute to upregulated cadherin binding [33]. The single downregulated DEP in the SSc-ILD vs. SSc w/o ILD contrast-dipeptidase 2 (DPEP), was shown to inhibit EMT in lung adenocarcinoma, raising the question of whether its downregulation in SSc-ILD might contribute to increased EMT [34].

These results align with our PPI network and MCL cluster analysis, which showed ECM organization, activation of matrix metalloproteinases (MMPs), and Rho GTPase clusters. SSc is associated with dysregulation of MMP activity, resulting in ECM accumulation and fibrosis [35]. Specifically, MMP-9 was among the upregulated DEPs in the SSc-ILD vs. CTRL contrast (FC = 3.55). Previous research has shown elevated MMP-9 levels in SSc patients correlating with skin fibrosis, while another study proposed serum MMP-9 levels for screening and evaluating severity in connective tissue disease-associated ILD [36,37]. RhoGTPases are implicated in SSc pathogenesis through regulation of fibroblasts and by promoting fibrogenesis [38,39]. One of the identified proteins in the RhoDGTPase cluster was Cav-1, which had the second highest fold change (FC = 6.3) among DEPs in the SSc-ILD vs. SSc contrast. It stands out as a potential biomarker for SSc-ILD due to its high fold change and potential pathogenic significance. A study by Albacete-Albacete et al. shows that Cav-1 is required for the EV sorting of ECM protein cargo and for fibroblast-derived EVs to deposit ECM in a tumour environment [40]. Although our dataset shows upregulated expression of Cav-1 in SSc-ILD compared with SSc w/o ILD and controls, previous studies have shown reduced expression of Cav-1 in SSc lung and skin tissues, which enhances TGF-β signalling and creates a feedback loop which amplifies fibrotic signalling [41,42]. Conversely, Cav-1 is a key EV cargo protein that regulates EV biogenesis, cargo sorting, and uptake [43]. Further studies should determine whether increased Cav-1 in EV-enriched fractions in SSc-ILD reflects altered EV regulation, enhanced EV-mediated release contributing to tissue depletion, or a compensatory response to profibrotic signalling.

Furthermore, type II alveolar epithelial cells (AECs) may have an important role in SSc-ILD, as they interact with fibroblasts, while AEC undergoing TGF-β-mediated mesenchymal transition in vitro can activate lung fibroblasts [44,45]. In this context, SP-B, a marker of type II AECs, also stands out as a potential SSc-ILD biomarker as the top overabundant protein (FC = 7.34) in the SSc-ILD vs. SSc w/o ILD contrast [46]. This is produced by type II AECs, which are crucial for promoting alveolar stability and may reflect epithelial injury and dysfunction, both important in SSc-ILD pathogenesis [8,47,48]. Enomoto et al. found that SP-B in serum EVs could serve as a biomarker for progressive pulmonary fibrosis, predicting ILD progression better than other candidate biomarkers [49]. However, studies evaluating SP-B in plasma and plasma EVs from SSc-ILD patient cohorts are still lacking.

In addition to ECM dysfunction, functional enrichment also pointed to immune dysregulation, while MCL clusters revealed immunoregulatory interactions and interleukin signalling. Aberrant immune activation and increased pro-inflammatory mediators are important hallmarks of SSc and SSc fibrosis [50]. Additionally, DEPs downregulated in the SSc-ILD vs. CTRL contrast were related to the regulation of apoptotic cell clearance. Dysregulation of apoptotic cell removal is associated with the initiation and progression of inflammation and autoimmunity, specifically in several chronic inflammatory respiratory diseases and systemic lupus erythematosus [51,52]. This may also implicate potential apoptosis dysregulation, as failure of myofibroblast apoptosis, a major source of ECM proteins, has been demonstrated as a key mechanism in mouse models of SSc–ILD [9,28,53]. Additionally, the upregulated DEPs from the SSc-ILD vs. CTRL contrast showed NET formation enrichment. NETs have been identified as key inflammatory mediators in lung fibrosis and shown to activate lung fibroblasts and contribute to their myofibroblast differentiation in lung fibrosis. They have also been implicated in ILD in idiopathic inflammatory myopathy and other fibrotic diseases, such as liver cirrhosis [54,55,56]. The role of the complement system in different phenotypes of SSc is unclear [57,58]. Possibly, altered toll-like receptor signalling, described in SSc patients, might trigger the complement cascade, resulting in hypocomplementemia and possibly influence disease pathogenesis [57].

In the MCL cluster related to immunoregulatory interactions, Siglec-5 stands out as one of the top three DEPs identified in the SSc-ILD vs. SSc w/o ILD contrast (FC = 4.3). Siglec-5 is expressed by innate immune cells, has immunomodulatory effects and acts as an inhibitory T cell immune checkpoint molecule [59]. Siglecs have been implicated as possible contributors in autoimmune diseases. For example, Siglec-1 is proposed to suppress expansion of regulatory T-cells (T regs) and thereby promote effector T cell proliferation and resulting inflammation and was even proposed as a possible biomarker for SSc [60,61]. Siglec-5 can supress NETosis by limiting neutrophil activation [62,63]. Whether increased Siglec-5 in EV-enriched fractions reflects increased shedding from activated immune cells or a compensatory, immune-modulating mechanism due to immune dysregulation in SSc, is yet to be elucidated.

Interestingly, several enriched biological processes and an MCL cluster are related to the regulation of systemic arterial blood pressure. This may reflect the emerging evidence of direct pathologic actions of EVs on vascular endothelial and smooth muscle cells in SSc. This is relevant considering endothelial dysfunction and vasculopathy are early hallmarks of the disease [64]. Specifically, endothelial dysfunction and endothelial-to-mesenchymal transition (EndMT) might be related to an inflammatory milieu leading towards fibrosis [64]. Not to be overlooked, there is the possibility that these results may reflect the existing evidence of EVs as potential biomarkers of pulmonary arterial hypertension (PAH), as PAH was present in some of the subjects in our study [12,64].

In addition to their potential pathophysiological roles in SSc-ILD, proteins Cav-1, SP-B and Siglec-5, as mentioned in their respective sections above, also stand out as potential biomarker candidates for SSc-ILD due to them being the top three most abundant DEPs and because of their pathophysiological rationale. Additionally, preliminary ROC curve analyses for distinguishing between SSc-ILD and SSc w/o ILD (conducted using the abundance levels as preliminary surrogates for concentrations), yielded high AUC, with accompanying satisfactory sensitivity and specificity values. Furthermore, all three proteins were positively correlated with Goh score and the presence of SSc-ILD, while SP-B and Siglec-5 were also negatively correlated with pulmonary function tests (DLco and FVC, respectively). Taken together, these results show promise and support the rationale for further validation studies for these proteins to test, confirm, and validate their biomarker applicability.

Our study has several limitations. Firstly, definitive conclusions are limited by the relatively small sample size, especially in the SSc-ILD group. Additional limitations arise from the inherent heterogeneity of SSc patients, which could drive proteomic differences independent of ILD. Moreover, proteomic differences can arise from treatments, most importantly immunosuppressants, which are expected to alter protein expression to some extent [65,66]. Furthermore, given the lack of comprehensive EV characterization, the analysed material likely represents plasma-derived EV-enriched fractions, which may include co-isolated plasma components, such as abundant plasma proteins like albumin, immunoglobulins, and fibrinogen [11,67]. This may influence LC-MS results by causing overrepresentation of pathways due to artifacts and potentially masking low-abundance EV-associated proteins [68]. Consequently, the results of this study, especially pathway-level findings, as well as biomarker candidates, should be verified in future studies.

In conclusion, our results suggest the presence of distinct proteomic signatures characterized by ECM and immune dysregulation in plasma-derived EV-enriched fractions of SSc patients. Notably, most of these alterations were observed in both SSc-ILD and SSc patients without ILD, indicating that they may primarily reflect underlying SSc pathophysiology rather than ILD-specific mechanisms. However, among the identified DEPs between patients with and without lung involvement, proteins SP-B, Siglec-5 and Cav-1 emerged as potential candidate biomarkers for SSc-ILD. However, these findings require validation in larger, independent cohorts to further elucidate the role of these molecules in SSc-ILD pathogenesis and their potential clinical utility in the diagnostic pathway of SSc-ILD.

## Figures and Tables

**Table 3:** Upregulated DEPs from the SSc-ILD vs. SSc w/o ILD contrast.

Accession (UNIPROT ID)	Name	Fold Change	Adjusted *p*-Value
P07988	Pulmonary surfactant-associated protein B (SP-B)	7.335835	0.004655
Q03135	Caveolin-1	6.296655	0.00684
O15389	Sialic acid-binding Ig-like lectin 5 (Siglec-5)	4.304996	0.037961
A0A0A0MS15	Immunoglobulin heavy variable 3–49	3.838354	0.030972
P13591	Neural cell adhesion molecule 1 (*N*-CAM-1)	3.771378	0.023597
P00739	Haptoglobin-related protein	3.660706	0.023597
P07360	Complement component C8 gamma chain	3.415022	0.004655
Q13449	Limbic system-associated membrane protein (LSAMP)	2.981821	0.037961
P55058	Phospholipid transfer protein	2.488206	0.040514
P01857	Immunoglobulin heavy constant gamma 1	2.436224	0.025819
A0A075B6K5	Immunoglobulin lambda variable 3–9	2.42701	0.015321
P18428	Lipopolysaccharide-binding protein (LBP)	2.32269	0.025819
O14791	Apolipoprotein L1	2.072121	0.025819
P01031	Complement C5	2.047894	0.046555

**Table 4:** Top 20 upregulated and downregulated DEPs in the SSc-ILD vs. CTRL contrast.

	Accession (UNIPROT ID)	Name	Fold Change	Adjusted *p*-Value
upregulated	Q9ULQ0	Striatin-interacting protein 2	18.74411149	1.13 × 10^−6^
	Q9NPR2	Semaphorin-4B (Semaphorin-C)	16.66200018	8.04855 × 10^−7^
	P68431;Q71DI3	Histone H3.1	15.4547124	0.001621601
	O60287	Nucleolar pre-ribosomal-associated protein 1	13.78001957	0.00280258
	O60927	E3 ubiquitin-protein ligase PPP1R11	12.99940701	0.00013344
	P02538	Keratin, type II cytoskeletal 6A	10.87661456	0.001316071
	P38405	Guanine nucleotide-binding protein G(olf) subunit alpha	9.828450904	4.58332 × 10^−6^
	Q6NZI2	Caveolae-associated protein 1	9.682145971	2.06336 × 10^−7^
	P37198	Nuclear pore glycoprotein p62	8.714891285	0.000101794
	Q86YZ3	Hornerin	8.097186865	6.11991 × 10^−5^
	P80511	Protein S100-A12	8.028541638	0.009800157
	P15880	Small ribosomal subunit protein uS5	7.371082661	4.48512 × 10^−5^
	P24394	Interleukin-4 receptor subunit alpha	7.231902661	9.09545 × 10^−6^
	Q9NRX4	14 kDa phosphohistidine phosphatase	7.033642369	0.001096678
	Q14667	Bridge-like lipid transfer protein family member 2	6.935468663	2.08111 × 10^−5^
	Q8NHG8	E3 ubiquitin-protein ligase ZNRF2	6.868960088	0.006077111
	Q13336	Urea transporter 1	6.682560505	4.25444 × 10^−5^
	P54277	PMS1 protein homolog 1	6.598724207	0.008162924
	P14209	CD99 antigen	6.560199542	2.68304 × 10^−8^
	Q99732	Lipopolysaccharide-induced tumour necrosis factor-alpha factor	6.227070448	0.004434099
downregulated	Q9Y5Y7	Lymphatic vessel endothelial hyaluronic acid receptor 1 (cell surface retention sequence-binding protein 1)	0.075364446	2.74418 × 10^−20^
	P18428	Lipopolysaccharide-binding protein	0.085178976	5.73801 × 10^−17^
	Q6UXB8	Peptidase inhibitor 16 (cysteine-rich secretory protein 9, CD antigen CD364)	0.096553388	1.20342 × 10^−8^
	P01706	Immunoglobulin lambda variable 2–11	0.098744172	6.85325 × 10^−5^
	P05976	Myosin light chain 1/3, skeletal muscle isoform	0.099062874	0.011687094
	Q9NP78	ABC-type oligopeptide transporter ABCB9	0.107659987	0.00416174
	Q9NX76	CKLF-like MARVEL transmembrane domain-containing protein 6 (chemokine-like factor superfamily member 6)	0.11139252	0.003035595
	Q96KN2	Beta-Ala-His dipeptidase (carnosine dipeptidase 1)	0.111648777	8.80909 × 10^−9^
	Q14165	Malectin	0.114369684	1.19073 × 10^−7^
	O75144	ICOS ligand (CD antigen CD275)	0.123449449	6.23239 × 10^−11^
	Q9UNN8	Endothelial protein C receptor (CD antigen CD201)	0.132663349	9.43238 × 10^−7^
	Q9UBX5	Fibulin-5	0.133414898	0.006894944
	A0A075B7D0	Immunoglobulin heavy variable 1/OR15-1	0.137902766	0.003430811
	Q76LX8	A disintegrin and metalloproteinase with thrombospondin motifs 13 (ADAMTS13)	0.151849663	9.09545 × 10^−6^
	P01709	Immunoglobulin lambda variable 2–8	0.159732028	0.000216383
	P05546	Heparin cofactor 2	0.164368558	5.22625 × 10^−5^
	P23470	Receptor-type tyrosine-protein phosphatase gamma	0.16808505	6.76723 × 10^−7^
	Q8TDY8	Immunoglobulin superfamily DCC subclass member 4	0.174862513	0.00379555
	Q86X83	COMM domain-containing protein 2	0.179234423	0.00085255
	P04180	Phosphatidylcholine-sterol acyltransferase	0.179585662	2.81494 × 10^−7^

**Table 5:** Top 20 upregulated and downregulated DEPs in the SSc w/o ILD vs. CTRL contrast.

	Accession (UNIPROT ID)	Name	Fold Change	Adjusted *p*-Value
upregulated	Q9ULQ0	Striatin-interacting protein 2	23.93245294	2.1051 × 10^−9^
	O60927	E3 ubiquitin-protein ligase PPP1R11 (Protein phosphatase inhibitor 3)	19.42844081	3.23597 × 10^−7^
	Q8NHG8	E3 ubiquitin-protein ligase ZNRF2 (Protein Ells2)	14.84281112	5.61536 × 10^−6^
	P38405	Guanine nucleotide-binding protein G(olf) subunit alpha	14.02383296	2.21444 × 10^−9^
	P37198	Nuclear pore glycoprotein p62	13.41152542	9.70748 × 10^−8^
	Q86SS6	Synaptotagmin-9	12.99348761	3.99894 × 10^−8^
	Q9NPR2	Semaphorin-4B (Semaphorin-C)	11.61553188	2.2456 × 10^−7^
	O60287	Nucleolar pre-ribosomal-associated protein 1	10.53104686	0.00092675
	P07766	T-cell surface glycoprotein CD3 epsilon chain	9.273320154	0.002090095
	P15880	Small ribosomal subunit protein uS5	9.231460378	1.51151 × 10^−7^
	Q9Y333	U6 snRNA-associated Sm-like protein LSm2	7.93380256	0.000151408
	P78417	Glutathione S-transferase omega-1	7.571078464	1.54844 × 10^−7^
	O43768	Alpha-endosulfine	7.492512944	1.3214 × 10^−7^
	Q9P0T7	Proton-transporting V-type ATPase complex assembly regulator TMEM9 (Transmembrane protein 9)	7.388282719	0.00089825
	Q14667	Bridge-like lipid transfer protein family member 2	7.16957502	3.9082 × 10^−7^
	P13797	Plastin-3	7.098533778	2.7286 × 10^−11^
	Q99952	Tyrosine-protein phosphatase non-receptor type 18	6.953636036	2.93535 × 10^−6^
	Q96MW1	Coiled-coil domain-containing protein 43	6.918166585	0.000133124
	Q9NRX4	14 kDa phosphohistidine phosphatase	6.788631149	0.000107243
	Q8TB36	Ganglioside-induced differentiation-associated protein 1 (GDAP1)	6.67368977	8.91272 × 10^−6^
downregulated	P18428	Lipopolysaccharide-binding protein	0.036672562	2.70586 × 10^−28^
	P01706	Immunoglobulin lambda variable 2–11	0.051992651	1.18711 × 10^−8^
	Q6UXB8	Peptidase inhibitor 16 (Cysteine-rich secretory protein 9 (CRISP-9))	0.063793375	4.68391 × 10^−14^
	P01709	Immunoglobulin lambda variable 2–8	0.064776155	1.45991 × 10^−10^
	A0A075B7D0	Immunoglobulin heavy variable 1/OR15-1	0.064831421	8.07474 × 10^−7^
	Q9Y5Y7	Lymphatic vessel endothelial hyaluronic acid receptor 1 (LYVE-1)	0.065683461	1.00064 × 10^−25^
	P51572	B-cell receptor-associated protein 31	0.065698529	2.24813 × 10^−7^
	P23470	Receptor-type tyrosine-protein phosphatase gamma	0.066267881	6.87916 × 10^−16^
	Q96KN2	Beta-Ala-His dipeptidase	0.076731962	3.17469 × 10^−14^
	P05546	Heparin cofactor 2	0.08276003	1.12134 × 10^−10^
	P05976	Myosin light chain 1/3, skeletal muscle isoform	0.083282479	0.000830285
	Q9NP78	ABC-type oligopeptide transporter ABCB9	0.085066002	9.13521 × 10^−5^
	A0A075B6I1	Immunoglobulin lambda variable 4–60	0.088296512	0.003144152
	O75144	ICOS ligand	0.09036279	1.37179 × 10^−16^
	P19652	Alpha-1-acid glycoprotein 2 (Orosomucoid-2)	0.091285018	6.19098 × 10^−11^
	P13591	Neural cell adhesion molecule 1	0.097334213	2.1088 × 10^−11^
	P22891	Vitamin K-dependent protein Z	0.097568465	5.75664 × 10^−9^
	Q14165	Malectin	0.098241368	1.6739 × 10^−10^
	A0A0A0MS15	Immunoglobulin heavy variable 3–49	0.098576793	3.5261 × 10^−10^
	Q7Z3E2	Coiled-coil domain-containing protein 186	0.108723603	0.000554537

## Data Availability

The mass spectrometry proteomics data have been deposited to the ProteomeXchange Consortium via the PRIDE partner repository [https://www.ebi.ac.uk/pride/archive] (accessed on 10 December 2025) with the dataset identifier PXD071797.

## Supplementary Materials

The following supporting information can be downloaded at https://www.mdpi.com/article/10.3390/diagnostics16121879/s1, Table S1: Full list of identified upregulated DEPs in the SSc-ILD vs. CTRL contrast. Table S2: Full list of identified downregulated DEPs in the SSc-ILD vs. CTRL contrast. Table S3: Full list of identified upregulated DEPs in the SSc w/o ILD vs. CTRL contrast. Table S4: Full list of identified downregulated DEPs in the SSc w/o ILD vs. CTRL contrast. Table S5: Results of functional enrichment analysis for the upregulated DEPs of the SSc-ILD vs. CTRL contrast showing top 20 significant enriched terms (or all significant results if less than 20 were identified) in all analysed pathway databases. Table S6: Results of functional enrichment analysis for the downregulated DEPs of the SSc-ILD vs. CTRL contrast showing top 20 significant enriched terms (or all significant results if less than 20 were identified) in all analysed pathway databases. Table S7: Results of functional enrichment analysis for the upregulated DEPs of the SSc w/o ILD vs. CTRL contrast showing top 20 significant enriched terms (or all significant results if less than 20 were identified) in all analysed pathway databases. Table S8: Results of functional enrichment analysis for the downregulated DEPs of the SSc w/o ILD vs. CTRL contrast showing top 20 significant enriched terms (or all significant results if less than 20 were identified) in all analysed pathway databases. Table S9: Pairwise AUC comparisons (DeLong’s Test) of the three biomarker candidate proteins. No statistically significant difference was found among them. Table S10: Correlation matrix of MAXLFQ, KNIME normalized data with %predicted values for diffusion capacity for CO (DLco), forced vital capacity (FVC), Goh score and immunosuppression (IS) (yes/no) for patients with SSc. Figure S1: PPI network for the upregulated DEPs from the SSc-ILD vs. CTRL contrast, (created using STRING v.12.0). Figure S2: PPI network for the downregulated DEPs from the SSc-ILD vs. CTRL contrast, (created using STRING v.12.0). Figure S3: Box plot comparing the distribution of expression (MAXLFQ, KNIME normalized values) of the biomarker candidate protein Siglec-5 between all subjects with SSc (the SSc-ILD and SSc w/o ILD groups combined) and the control (CTRL) group. Figure S4: Box plot comparing distribution of expression (MAXLFQ, KNIME normalized values) of the biomarker candidate protein Cav-1 between all subjects with SSc (the SSc-ILD and SSc w/o ILD groups combined) and the control (CTRL) group. Figure S5: Box plot comparing distribution of expression (MAXLFQ, KNIME normalized values) of the biomarker candidate protein SP-B between all subjects with SSc (the SSc-ILD and SSc w/o ILD groups combined) and the control (CTRL) group.
