# Supplementary material for: Plasma-Derived Extracellular Vesicle-Enriched Fractions as a Potential Source of Biomarkers for Systemic Sclerosis-Associated Interstitial Lung Disease (SSc-ILD): A Shotgun Proteomic Exploration Analysis
Source: Diagnostics (Basel). 2026 Jun 17;16(12):1879. doi: 10.3390/diagnostics16121879 (PMC13297690; doi:10.3390/diagnostics16121879)
Supplement: Supplementary file 1 [file diagnostics-16-01879-s001.zip › diagnostics-4336815-supplementary.pdf]

Table S1: Full list of identified upregulated DEPs in the SSc-ILD vs. CTRL contrast.

Accession (UNIPROT ID)	Name	Fold change	Adjusted p-value
Q9ULQ0	Striatin-interacting protein 2	18.74411149	1.13E-06
Q9NPR2	Semaphorin-4B (Semaphorin-C)	16.66200018	8.05E-07
P68431;Q71DI3	Histone H3.1	15.4547124	0.001622
O60287	Nucleolar pre-ribosomal-associated protein 1	13.78001957	0.002803
O60927	E3 ubiquitin-protein ligase PPP1R11	12.99940701	0.000133
P02538	Keratin, type II cytoskeletal 6A	10.87661456	0.001316
P38405	Guanine nucleotide-binding protein G(olf) subunit alpha	9.828450904	4.58E-06
Q6NZI2	Caveolae-associated protein 1	9.682145971	2.06E-07
P37198	Nuclear pore glycoprotein p62	8.714891285	0.000102
Q86YZ3	Hornerin	8.097186865	6.12E-05
P80511	Protein S100-A12	8.028541638	0.0098
P15880	Small ribosomal subunit protein uS5	7.371082661	4.49E-05
P24394	Interleukin-4 receptor subunit alpha	7.231902661	9.1E-06
Q9NRX4	14 kDa phosphohistidine phosphatase	7.033642369	0.001097
Q14667	Bridge-like lipid transfer protein family member 2	6.935468663	2.08E-05
Q8NHG8	E3 ubiquitin-protein ligase ZNRF2	6.868960088	0.006077
Q13336	Urea transporter 1	6.682560505	4.25E-05
P54277	PMS1 protein homolog 1	6.598724207	0.008163
P14209	CD99 antigen	6.560199542	2.68E-08
Q99732	Lipopolysaccharide-induced tumour necrosis factor-alpha factor	6.227070448	0.004434
Q9C075	Keratin, type I cytoskeletal 23	5.901310906	0.032676
Q0VGL1	Ragulator complex protein LAMTOR4	5.683580301	0.006864
Q86SS6	Synaptotagmin-9	5.640873585	0.00103
O75367	Core histone macro-H2A.1	5.525657595	0.004824
P04216	Thy-1 membrane glycoprotein (CD antigen CD90)	5.470469898	0.00105
P62424	Large ribosomal subunit protein eL8	5.292744302	0.000146
Q9UBI6	Guanine nucleotide-binding protein G(I)/G(S)/G(O) subunit gamma-12	5.280700794	6.85E-05
P24071	Immunoglobulin alpha Fc receptor (CD antigen CD89)	5.192685887	0.017514
O00743	Serine/threonine-protein phosphatase 6 catalytic subunit	5.174249327	0.016913
Q8TB36	Ganglioside-induced differentiation-associated protein 1	5.153025472	0.001195
Q99952	Tyrosine-protein phosphatase non-receptor type 18	5.067718202	0.00083
P80723	Brain acid soluble protein 1	5.049292164	3.35E-08
Q96DR8	Mucin-like protein 1	5.017383137	0.002358
Q8WXI8	C-type lectin domain family 4 member D	4.976193628	0.003013
P27701	CD82 antigen (Suppressor of tumorigenicity 6 protein) (Tetraspanin-27)	4.957214136	0.002462
P16452	Protein 4.2	4.865801606	0.002419
P13797	Plastin-3	4.805433126	3.56E-06
P18077	Large ribosomal subunit protein eL33	4.780553748	0.002473
Q9NQ34	Transmembrane protein 9B	4.675848849	0.000222
O95819	Mitogen-activated protein kinase kinase kinase kinase 4	4.587522898	0.007453
O43237	Cytoplasmic dynein 1 light intermediate chain 2	4.562319974	0.02678

Q9UGM3	Scavenger receptor cysteine-rich domain-containing protein DMBT1	4.549802166	0.004849
P81605	Dermcidin	4.519621643	2.63E-05
O75871	Cell adhesion molecule CEACAM4	4.480273572	0.000417
P04259	Keratin, type II cytoskeletal 6B	4.419239661	0.040488
Q9Y333	U6 snRNA-associated Sm-like protein LSm2	4.374940144	0.027577
Q9Y336	Sialic acid-binding Ig-like lectin 9 (CD antigen CD329)	4.354089144	0.010579
P78417	Glutathione S-transferase omega-1	4.336825236	0.001716
Q9H246	Uncharacterized protein C1orf21	4.254859467	0.007027
P39023	Large ribosomal subunit protein uL3	4.251659497	0.019818
O43175	D-3-phosphoglycerate dehydrogenase	4.249535276	0.000734
P42166	Lamina-associated polypeptide 2, isoform alpha	4.171763891	0.000167
O15427	Monocarboxylate transporter 4	4.167686377	0.048981
P59666;P59665	Neutrophil defensin 1/3	4.166260801	0.000524
Q15323	Keratin, type I cuticular Ha1	4.161750366	0.042107
P53004	Biliverdin reductase A	4.090842762	0.000441
P29508	Serpin B3	4.015919129	0.013678
P06899	Histone H2B type 1-J	4.007267674	0.024516
Q92890	Ubiquitin recognition factor in ER-associated degradation protein 1	3.997140428	0.019727
Q9Y287	Integral membrane protein 2B	3.991545334	0.000414
P26599	Polypyrimidine tract-binding protein 1	3.966811386	0.024966
P63220	Small ribosomal subunit protein eS21	3.923026138	0.000201
Q03135	Caveolin-1	3.910391126	0.005451
Q96MW1	Coiled-coil domain-containing protein 43	3.900025293	0.027888
P08246	Neutrophil elastase (Bone marrow serine protease)	3.882420565	0.018568
Q96M27	Protein PRRC1	3.833739453	0.004616
P14550	Aldo-keto reductase family 1 member A1	3.80451484	0.046144
O95969	Secretoglobin family 1D member 2	3.803418987	0.010579
P16989	Y-box-binding protein 3	3.77781223	0.001277
Q8NFU3	Thiosulfate:glutathione sulfurtransferase	3.751085731	0.000638
Q13574	Diacylglycerol kinase zeta	3.745154404	0.024859
P06733	Alpha-enolase	3.727462567	3.31E-06
P20290	Transcription factor BTF3	3.72210799	3.62E-05
P49768	Presenilin-1	3.705082728	0.036261
Q3I5F7;Q86TX2	Acyl-coenzyme A thioesterase 6	3.702431435	0.012351
Q8IV03	Leucine rich adaptor protein 1-like	3.670813823	0.046158
O00629	Importin subunit alpha-3	3.65984545	0.033051
P56385	ATP synthase F(0) complex subunit e, mitochondrial	3.640515883	0.001688
P26373	Large ribosomal subunit protein eL13	3.63211923	0.002998
Q9UN19	Dual adapter for phosphotyrosine and 3-phosphotyrosine and 3-phosphoinositide	3.6158877	0.032069
P29466	Caspase-1 (Interleukin-1 beta-converting enzyme)	3.598225323	0.000638
P62942	Peptidyl-prolyl cis-trans isomerase FKBP1A	3.594070454	0.009001
Q9NRV9	Heme-binding protein 1	3.559003756	0.012351
P14780	Matrix metalloproteinase-9	3.548679486	0.005127
P50995	Annexin A11	3.542557019	0.044631
Q71RC9	Small integral membrane protein 5	3.53729419	0.010077

Q9NRW7	Vacuolar protein sorting-associated protein 45	3.529301722	2.79E-05
O14957	Cytochrome b-c1 complex subunit 10	3.499515283	0.028112
P08311	Cathepsin G	3.484816658	0.016345
P49721	Proteasome subunit beta type-2	3.476325298	0.025619
P61313	Large ribosomal subunit protein eL15	3.467573688	0.010869
O43768	Alpha-endosulfine	3.461330901	0.004441
A0A0B4J1V6	Immunoglobulin heavy variable 3-73	3.429796811	0.007742
P04899	Guanine nucleotide-binding protein G(i) subunit alpha-2	3.425035901	0.000845
O43914	TYRO protein tyrosine kinase-binding protein	3.420011147	0.000163
Q9Y376	Calcium-binding protein 39	3.366685078	0.010579
Q96FS4	Signal-induced proliferation-associated protein 1	3.36505015	0.002544
P13647	Keratin, type II cytoskeletal 5	3.36414457	0.017514
Q7Z403	Transmembrane channel-like protein 6	3.34701198	0.037005
B2RUZ4	Small integral membrane protein 1	3.335517386	1.28E-05
Q8WWW8	GRB2-associated-binding protein 3	3.302544891	0.022979
Q99062	Granulocyte colony-stimulating factor receptor	3.296833354	0.024665
Q92608	Dedicator of cytokinesis protein 2	3.278757857	0.006475
P55795	Heterogeneous nuclear ribonucleoprotein H2	3.270326503	0.006127
P40121	Macrophage-capping protein	3.266969309	0.003706
P46940	Ras GTPase-activating-like protein IQGAP1	3.252609164	0.008201
Q8TF74	WAS/WASL-interacting protein family member 2	3.221467619	0.003706
P10645	Chromogranin-A	3.19841529	0.026293
P19338	Nucleolin	3.156096201	0.0123
Q8NFZ5	TNFAIP3-interacting protein 2	3.131400253	0.04144
Q8IUW5	RELT-like protein 1	3.130190893	0.002461
P46779	Large ribosomal subunit protein eL28	3.124655475	0.006369
O00241	Signal-regulatory protein beta-1 (CD antigen CD172b)	3.104540341	0.007942
P48556	26S proteasome non-ATPase regulatory subunit 8	3.103348996	0.01254
P30566	Adenylosuccinate lyase	3.089364408	0.020318
Q92743	Serine protease HTRA1	3.081839435	0.022843
P29966	Myristoylated alanine-rich C-kinase substrate	3.065262479	4.61E-06
Q7Z4W1	L-xylulose reductase	3.056877684	0.032069
Q13191	E3 ubiquitin-protein ligase CBL-B	3.037748954	0.024519
Q8N7R7	Cyclin-Y-like protein 1	3.032404478	0.037227
O94760	N(G),N(G)-dimethylarginine dimethylaminohydrolase 1	3.022924137	0.029303
P05388	Large ribosomal subunit protein uL10	3.018440967	0.007489
Q9BZL6	Serine/threonine-protein kinase D2	3.01173236	0.046198
P35527	Keratin, type I cytoskeletal 9	3.010626561	0.036369
P37108	Signal recognition particle 14 kDa protein	2.988295492	0.013847
P52597	Heterogeneous nuclear ribonucleoprotein F	2.982111579	0.002537
P62750	Large ribosomal subunit protein uL23	2.965897519	0.00154
P83731	Large ribosomal subunit protein eL24	2.953662175	0.037532
Q09327	Beta-1,4-mannosyl-glycoprotein 4-beta-N-acetylglucosaminyltransferase	2.949490324	0.036685
P13716	Delta-aminolevulinic acid dehydratase	2.944019637	0.026349
O43566	Regulator of G-protein signalling 14	2.935373284	0.045367
P62081	Small ribosomal subunit protein eS7	2.926913194	0.000878

Q96IG2	F-box/LRR-repeat protein 20	2.871568189	0.038725
O15389	Sialic acid-binding Ig-like lectin 5	2.863771865	0.032119
Q9NP97	Dynein light chain roadblock-type 1	2.853612098	0.000545
P05089	Arginase-1	2.847742069	0.031997
P46776	Large ribosomal subunit protein uL15	2.840045109	0.010869
P04264	Keratin, type II cytoskeletal 1	2.832143174	0.014939
Q02878	Large ribosomal subunit protein eL6	2.814108283	0.008278
Q15637	Splicing factor 1 (Zinc finger protein 162)	2.814106965	0.00547
P08708	Small ribosomal subunit protein eS17	2.811497759	0.001396
P35236	Tyrosine-protein phosphatase non-receptor type 7 (Hematopoietic protein-tyrosine phosphatase)	2.811169403	0.043444
P09211	Glutathione S-transferase P	2.803393559	0.026527
P34897	Serine hydroxymethyltransferase, mitochondrial	2.802599558	0.019818
P33121	Long-chain-fatty-acid–CoA ligase 1	2.794832985	0.042797
Q92734	Protein TFG	2.772137438	0.010969
Q3YEC7	Rab-like protein 6	2.766064794	0.031997
P49411	Elongation factor Tu, mitochondrial	2.748139921	0.002718
P48507	Glutamate–cysteine ligase regulatory subunit	2.730090073	0.002473
P05164	Myeloperoxidase (MPO)	2.718867517	0.001697
Q86VB7	Scavenger receptor cysteine-rich type 1 protein M130	2.710379898	0.006133
Q92854	Semaphorin-4D (CD antigen CD100)	2.679392501	0.027653
Q9H1K6	Talin rod domain-containing protein 1	2.672802938	0.032504
P62906	Large ribosomal subunit protein uL1	2.660089892	0.000518
P06744	Glucose-6-phosphate isomerase	2.642215802	0.002214
P43250	G protein-coupled receptor kinase 6	2.634652871	0.002734
P61247	Small ribosomal subunit protein eS1	2.63182557	0.007353
P62917	Large ribosomal subunit protein uL2	2.627645787	0.023963
Q15046	Lysine–tRNA ligase	2.616988185	0.022614
P36957	Dihydrolipoyllysine-residue succinyltransferase component of 2-oxoglutarate dehydrogenase complex, mitochondrial	2.60624038	0.013916
P55196	Afadin	2.599017609	0.027888
Q7Z6I6	Rho GTPase-activating protein 30	2.591223684	0.025067
P60900	Proteasome subunit alpha type-6	2.583708185	0.04982
P37837	Transaldolase	2.581665288	0.000562
P32119	Peroxiredoxin-2 (natural killer cell-enhancing factor B)	2.571047022	0.039301
P49795	Regulator of G-protein signalling 19	2.548336878	0.00593
Q5JWF2	Guanine nucleotide-binding protein G(s) subunit alpha isoforms XLas	2.542002168	0.035031
P69905	Haemoglobin subunit alpha	2.525856582	0.001414
Q9UGI8	Testin	2.515769245	0.024588
Q9Y5W9	Sorting nexin-11	2.512199198	0.013916
Q9NRR8	CDC42 small effector protein 1	2.507082707	0.015407
Q9BSL1	Ubiquitin-associated domain-containing protein 1	2.491623793	0.027577
P08493	Matrix Gla protein	2.483058735	0.004528
P30273	High affinity immunoglobulin epsilon receptor subunit gamma	2.47602218	0.004455
Q5HYW2	NHS-like protein 2	2.460772403	0.010284
Q96EP5	DAZ-associated protein 1	2.457530612	0.033051

Q9NY25	C-type lectin domain family 5 member A	2.422189536	0.037036
Q8N434	Putative transporter SVOPL	2.404697747	0.000533
Q9Y3E2	BolA-like protein 1	2.390423497	0.011915
P62241	Small ribosomal subunit protein eS8	2.384368704	0.023811
Q07960	Rho GTPase-activating protein 1	2.384143988	0.024453
P06729	T-cell surface antigen CD2	2.372004247	0.023051
P04083	Annexin A1	2.355644255	0.047893
P62266	Small ribosomal subunit protein uS12	2.354371991	0.004328
P14174	Macrophage migration inhibitory factor	2.348253669	0.005156
Q9NZL9	Methionine adenosyltransferase 2 subunit beta	2.345131634	0.011609
Q15185	Prostaglandin E synthase 3	2.340554067	0.007102
P01116	GTPase KRas	2.305140122	0.037192
P09543	2',3'-cyclic-nucleotide 3'-phosphodiesterase	2.30012312	0.018197
P02788	Lactotransferrin	2.282181953	0.023353
Q9Y5Y0	Choline/ethanolamine transporter FLVCR1	2.273219469	0.010579
P61106	Ras-related protein Rab-14	2.270453621	0.035114
Q9NR56	Muscleblind-like protein 1	2.261639162	0.029044
P07333	Macrophage colony-stimulating factor 1 receptor	2.241988671	0.022819
P13987	CD59 glycoprotein (MAC-inhibitory protein)	2.240010289	0.002053
P30041	Peroxiredoxin-6	2.235575823	0.019866
P07355	Annexin A2	2.2315299	0.02785
Q9BUH6	Protein PAXX	2.231305132	0.009664
Q9H4G4	Golgi-associated plant pathogenesis-related protein 1	2.228428573	2E-05
P04075	Fructose-bisphosphate aldolase A	2.21794769	0.004747
B0I1T2	Unconventional myosin-Ig	2.205133801	0.01039
P62913	Large ribosomal subunit protein uL5	2.195828663	0.026293
Q9NTK5	Obg-like ATPase 1	2.193490913	0.017681
O00233	26S proteasome non-ATPase regulatory subunit 9	2.190853437	0.009384
P50552	Vasodilator-stimulated phosphoprotein	2.154203248	0.010768
Q9UBW5	Bridging integrator 2	2.153915438	0.006126
P04839	NADPH oxidase 2	2.14583254	0.049353
Q9UQ80	Proliferation-associated protein 2G4 (ErbB3-binding protein 1)	2.145181374	0.0124
Q96FE7	Phosphoinositide-3-kinase-interacting protein 1	2.136180251	0.014071
Q9Y2D4	Exocyst complex component 6B	2.135075249	0.032877
Q13469	Nuclear factor of activated T-cells, cytoplasmic 2	2.132520802	0.046382
Q9UEY8	Gamma-adducin	2.129610565	0.032153
Q04759	Protein kinase C theta type	2.125698279	0.043224
P02786	Transferrin receptor protein 1	2.114363351	0.011945
Q96C19	EF-hand domain-containing protein D2	2.088488958	0.003113
P04040	Catalase	2.084245765	0.010075
P37840	Alpha-synuclein	2.07570109	0.00691
Q9HD89	Resistin	2.042453869	0.011344
Q14242	P-selectin glycoprotein ligand 1	2.024409802	0.017531
Q9H0E2	Toll-interacting protein	2.020612615	0.005749
P13639	Elongation factor 2	2.016175051	0.04772

P63218	Guanine nucleotide-binding protein G(I)/G(S)/G(O) subunit gamma-5	2.011356009	0.02678

Table S2: Full list of identified downregulated DEPs in the SSc-ILD vs. CTRL contrast.

Accession (UNIPROT ID)	Name	Fold change	Adjusted p-value
Q9Y5Y7	Lymphatic vessel endothelial hyaluronic acid receptor 1(Cell surface retention sequence-binding protein 1)	0.075364446	2.74418E-20
P18428	Lipopolysaccharide-binding protein	0.085178976	5.73801E-17
Q6UXB8	Peptidase inhibitor 16 (Cysteine-rich secretory protein 9, CD antigen CD364)	0.096553388	1.20342E-08
P01706	Immunoglobulin lambda variable 2-11	0.098744172	6.85325E-05
P05976	Myosin light chain 1/3, skeletal muscle isoform	0.099062874	0.011687094
Q9NP78	ABC-type oligopeptide transporter ABCB9	0.107659987	0.00416174
Q9NX76	CKLF-like MARVEL transmembrane domain-containing protein 6 (Chemokine-like factor superfamily member 6)	0.11139252	0.003035595
Q96KN2	Beta-Ala-His dipeptidase (Carnosine dipeptidase 1)	0.111648777	8.80909E-09
Q14165	Malectin	0.114369684	1.19073E-07
O75144	ICOS ligand (CD antigen CD275)	0.123449449	6.23239E-11
Q9UNN8	Endothelial protein C receptor (CD antigen CD201)	0.132663349	9.43238E-07
Q9UBX5	Fibulin-5	0.133414898	0.006894944
A0A075B7D0	Immunoglobulin heavy variable 1/OR15-1	0.137902766	0.003430811
Q76LX8	A disintegrin and metalloproteinase with thrombospondin motifs 13	0.151849663	9.09545E-06
P01709	Immunoglobulin lambda variable 2-8	0.159732028	0.000216383
P05546	Heparin cofactor 2	0.164368558	5.22625E-05
P23470	Receptor-type tyrosine-protein phosphatase gamma	0.16808505	6.76723E-07
Q8TDY8	Immunoglobulin superfamily DCC subclass member 4	0.174862513	0.00379555
Q86X83	COMM domain-containing protein 2	0.179234423	0.00085255
P04180	Phosphatidylcholine-sterol acyltransferase	0.179585662	2.81494E-07
P20851	C4b-binding protein beta chain	0.179677636	4.29703E-07
P19022	Cadherin-2 (N-cadherin, CD antigen CD325)	0.181184934	8.80909E-09
P05160	Coagulation factor XIII B chain	0.182882862	9.75277E-06
Q14789	Golgin subfamily B member 1	0.189484081	0.013361485
P51572	B-cell receptor-associated protein 31	0.190738584	0.00612461
Q9NVI7;Q5T9A4	ATPase family AAA domain-containing protein 3A;ATPase family AAA domain-containing protein 3B	0.197653663	0.002544065
Q15389	Angiopoietin-1	0.199838623	0.003989289
Q9Y6D6	Brefeldin A-inhibited guanine nucleotide-exchange protein 1	0.201818972	0.000900375

Q14314	Fibroleukin (Fibrinogen-like protein 2)	0.206173077	2.79321E-05
P05452	Tetranectin	0.207743766	1.20342E-08
P06276	Cholinesterase	0.207760368	0.000113345
Q9HCN8	Stromal cell-derived factor 2-like protein 1	0.208753224	0.003189807
P54289	Voltage-dependent calcium channel subunit alpha-2/delta-1	0.210457003	6.26166E-05
O75396	Vesicle-trafficking protein SEC22b	0.212323179	0.012351488
Q14126	Desmoglein-2 (Cadherin family member 5)	0.214498031	9.37153E-06
P20036	HLA class II histocompatibility antigen, DP alpha 1 chain	0.21479712	0.000272669
O60279	Sushi domain-containing protein 5	0.218186898	0.006458157
P01019	Angiotensinogen (Serpin A8)	0.219485264	1.50983E-07
P41222	Prostaglandin-H2 D-isomerase	0.219583743	8.10748E-05
O00533	Neural cell adhesion molecule L1-like protein	0.220342165	2.81588E-09
P35858	Insulin-like growth factor-binding protein complex acid labile subunit	0.223508683	6.06181E-06
P08185	Corticosteroid-binding globulin (Serpin A6)	0.225518212	2.60955E-10
Q9NZA1	Chloride intracellular channel protein 5	0.225899995	0.002544065
Q14392	Transforming growth factor beta activator LRRC32	0.22633787	0.003360159
Q99653	Calcineurin B homologous protein 1	0.226918597	0.000197149
P00387	NADH-cytochrome b5 reductase 3	0.22755984	0.000374285
O75298	Reticulon-2 (Neuroendocrine-specific protein-like 1)	0.227769464	0.010874668
P02751	Fibronectin	0.229120336	0.00677966
P0C0L5	Complement C4-B	0.229841474	7.62516E-09
Q9H4A9	Dipeptidase 2	0.230257139	1.19073E-07
P19652	Alpha-1-acid glycoprotein 2 (Orosomucoid-2)	0.230409494	0.000541837
O00391	Sulfhydryl oxidase 1 (Quiescin Q6)	0.231351247	1.07084E-06
P00450	Ceruloplasmin	0.231992772	9.43238E-07
Q16610	Extracellular matrix protein 1	0.239264939	0.003793995
P19823	Inter-alpha-trypsin inhibitor heavy chain H2	0.240466925	1.17792E-08
P18850	Cyclic AMP-dependent transcription factor ATF-6 alpha	0.241418024	4.26133E-05
P43652	Afamin	0.242267991	1.43373E-07
P04278	Sex hormone-binding globulin	0.24253919	0.001316071
P19827	Inter-alpha-trypsin inhibitor heavy chain H1	0.2452365	7.62516E-09
P04003	C4b-binding protein alpha chain	0.245718125	8.30792E-07

P63267	Actin, gamma-enteric smooth muscle (Alpha-actin-3)	0.246069827	0.012716943
P06681	Complement C2	0.246673865	1.083E-11
Q86UP2	Kinectin (CG-1 antigen)	0.248068944	0.000545297
P12821	Angiotensin-converting enzyme	0.250068086	0.010868955
Q86UN3	Reticulon-4 receptor-like 2	0.250561101	8.06263E-06
P0C0L4	Complement C4-A	0.250855197	0.002594423
Q92499	ATP-dependent RNA helicase DDX1	0.251516896	0.010252121
Q96RI0	Proteinase-activated receptor 4	0.251918955	0.012351488
A0A087WW87;P01614	Immunoglobulin kappa variable 2-40;immunoglobulin kappa variable 2D-40	0.252421766	0.000553137
P51884	Lumican	0.253012264	5.66202E-08
Q9UBV8	Peflin	0.25348836	0.00487781
P57735	Ras-related protein Rab-25	0.253925844	0.017530815
A0A0J9YY99	Ig-like domain-containing protein	0.255651399	0.000268128
Q9Y6R7	IgGFc-binding protein	0.260832007	7.78889E-06
O75882	Attractin	0.261582496	2.35575E-08
P00734	Prothrombin	0.264453602	8.29539E-06
P15151	Poliovirus receptor (Nectin-like protein 5)	0.270003754	0.000147574
P07357	Complement component C8 alpha chain	0.270422623	9.43238E-07
P36955	Pigment epithelium-derived factor (Serpin F1)	0.272177485	7.78889E-06
Q86TH1	ADAMTS-like protein 2	0.274917331	0.010862519
P07225	Vitamin K-dependent protein S	0.276346425	7.72292E-08
Q96IY4	Carboxypeptidase B2	0.278128452	0.004210502
Q15582	Transforming growth factor-beta-induced protein ig-h3	0.281283482	7.78889E-06
P60660	Myosin light polypeptide 6	0.282080349	0.005796007
P52815	Large ribosomal subunit protein bL12m	0.282502691	0.010747157
Q6ZNJ1	Neurobeachin-like protein 2	0.285207863	0.020972991
P19320	Vascular cell adhesion protein 1	0.285622733	1.30338E-05
Q14118	Dystroglycan 1	0.288386836	7.78889E-06
Q9UHL4	Dipeptidyl peptidase 2	0.291474907	0.008930624
A0A0B4J1Y9	Immunoglobulin heavy variable 3-72	0.292919669	0.004711593
O00585	C-C motif chemokine 21	0.293617616	0.00192502
P50416	Carnitine O-palmitoyltransferase 1, liver isoform	0.295808209	0.049148177

P14927	Cytochrome b-c1 complex subunit 7	0.297597195	0.008868279
P51153	Ras-related protein Rab-13 (Cell growth-inhibiting gene 4 protein)	0.297754603	0.005037804
A0A0B4J1U3	Immunoglobulin lambda variable 1-36	0.299482977	0.006344165
Q6ZMP0	Thrombospondin type-1 domain-containing protein 4 (ADAMTS-like protein 6)	0.300667007	0.016506949
P02750	Leucine-rich alpha-2-glycoprotein	0.303231913	4.76998E-05
P14543	Nidogen-1 (Entactin)	0.304390589	0.000562286
P16109	P-selectin (Leukocyte-endothelial cell adhesion molecule 3)	0.304647259	4.7057E-05
P61601	Neurocalcin-delta	0.304698953	0.006048947
Q96AG4	Leucine-rich repeat-containing protein 59	0.305742111	0.022540971
P06858	Lipoprotein lipase	0.306785312	0.001924771
Q9Y608	Leucine-rich repeat flightless-interacting protein 2	0.308176735	0.006135238
P35579	Myosin-9	0.309693101	0.00194945
Q14515	SPARC-like protein 1 (Hevin)	0.310341495	0.000424782
Q9Y646	Carboxypeptidase Q	0.313978457	8.19345E-05
P55058	Phospholipid transfer protein	0.315273561	0.00013103
P35443	Thrombospondin-4	0.315619261	0.013846575
P16671	Platelet glycoprotein 4 (Fatty acid translocase, Leukocyte differentiation antigen CD36)	0.317155397	0.002544065
P26927	Hepatocyte growth factor-like protein (Macrophage stimulatory protein)	0.318699448	0.000401123
P11047	Laminin subunit gamma-1	0.318980258	0.039782882
P17813	Endoglin (CD antigen CD105)	0.319182714	2.75812E-06
Q9BRV8	Suppressor of IKBKE 1	0.320432169	0.038725079
P21926	CD9 antigen (Motility-related protein)	0.320502993	0.004762967
Q9NRL3	Striatin-4 (Zinedin)	0.32094344	0.013257125
Q15113	Procollagen C-endopeptidase enhancer 1	0.323114491	0.000951698
O14960	Leukocyte cell-derived chemotaxin-2	0.323403108	0.003035595
Q9UKU6	Thyrotropin-releasing hormone-degrading ectoenzyme	0.323928057	0.018567508
P61586	Transforming protein RhoA	0.327864531	0.007809492
Q9UK55	Protein Z-dependent protease inhibitor (Serpin A10)	0.330714997	3.84408E-06
P98160	Basement membrane-specific heparan sulfate proteoglycan core protein	0.331688929	0.024518548
P30530	Tyrosine-protein kinase receptor UFO	0.3347692	0.035538048
Q14BN4	Sarcolemmal membrane-associated protein	0.336786246	0.010747157
P02675	Fibrinogen beta chain	0.337635051	0.001540356

O14498	Immunoglobulin superfamily containing leucine-rich repeat protein	0.337711128	0.011915209
P22059	Oxysterol-binding protein 1	0.338328903	0.032118718
O00625	Pirin	0.338954982	0.015976636
Q92614	Unconventional myosin-XVIIIa (Surfactant protein receptor SP-R210)	0.34198292	0.038349285
Q16627	C-C motif chemokine 14	0.342762318	0.007942378
P30825	High affinity cationic amino acid transporter 1	0.344467795	0.023097868
P22792	Carboxypeptidase N subunit 2	0.344641048	2.62789E-05
P12109	Collagen alpha-1(VI) chain	0.34692352	0.01634494
P22891	Vitamin K-dependent protein Z	0.349068453	0.032785253
Q8IUL8	Cartilage intermediate layer protein 2	0.349351212	0.002803702
P42892	Endothelin-converting enzyme 1	0.350369451	0.010266024
Q9Y2A7	Nck-associated protein 1	0.351866034	0.031868502
O15247	Chloride intracellular channel protein 2 (Glutaredoxin-like oxidoreductase CLIC2)	0.351982739	0.024631199
Q15075	Early endosome antigen 1	0.352457052	0.006458157
Q13740	CD166 antigen (Activated leukocyte cell adhesion molecule)	0.352661965	3.6348E-05
Q9NZM3	Intersectin-2	0.353678441	0.013306142
P02679	Fibrinogen gamma chain	0.355686143	0.007986603
Q9Y6C2	EMILIN-1 (Elastin microfibril interface-located protein 1)	0.357531785	0.019715029
P04070	Vitamin K-dependent protein C	0.358770664	0.00023424
P01780	Immunoglobulin heavy variable 3-7	0.35923674	0.00058363
P00533	Epidermal growth factor receptor	0.360070174	0.002544065
Q9NZP8	Complement C1r subcomponent-like protein	0.360681809	7.78889E-06
Q9Y210	Short transient receptor potential channel 6 (Transient receptor protein 6)	0.361832414	0.006368793
P08603	Complement factor H	0.362194914	0.000165223
P01859	Immunoglobulin heavy constant gamma 2	0.362717286	0.000221622
Q13201	Multimerin-1 (Elastin microfibril interface located protein 4)	0.364412192	0.006077111
Q5SQ64	Lymphocyte antigen 6 complex locus protein G6f	0.364981331	0.039791926
Q53HC0	Coiled-coil domain-containing protein 92	0.365580423	0.026779863
P15586	N-acetylglucosamine-6-sulfatase	0.366928722	0.048457146
P13591	Neural cell adhesion molecule 1 (CD antigen CD56)	0.367084132	0.014283222
P48740	Mannan-binding lectin serine protease 1 (Complement factor MASP-3)	0.36721473	0.00677966
P07996	Thrombospondin-1 (Glycoprotein G)	0.367533979	0.016013617

O75976	Carboxypeptidase D	0.3685362 33	0.0310605 19
Q9Y696	Chloride intracellular channel protein 4 (Glutaredoxin-like oxidoreductase CLIC4)	0.3686172 33	0.0010754 87
P04179	Superoxide dismutase [Mn], mitochondrial	0.3689007 13	0.0108746 68
Q9BWP8	Collectin-11	0.3693947 07	0.0101312 22
Q04756	Hepatocyte growth factor activator serine protease	0.3710097 5	0.0001665 43
P00751	Complement factor B	0.3718173 38	0.0003454 97
Q9Y251	Heparanase (Endo-glucoronidase)	0.3731281 21	0.0398587 08
P01023	Alpha-2-macroglobulin	0.3750041 72	6.26166E-05
P19404	NADH dehydrogenase [ubiquinone] flavoprotein 2, mitochondrial	0.3757495 59	0.0483802 22
Q8WWI5	Choline transporter-like protein 1	0.3758983 78	0.0003665 98
Q9BSF0	Small membrane A-kinase anchor protein	0.3772431 31	0.0211267 22
A0A0A0MS15	Immunoglobulin heavy variable 3-49	0.3783726 06	0.0304230 58
P01009	Alpha-1-antitrypsin	0.3829183 52	1.99915E-05
P01008	Antithrombin-III (Serpin C1)	0.3832505 36	1.19073E-07
Q15223	Nectin-1 (Nectin cell adhesion molecule 1, CD antigen CD111)	0.3855926 04	0.0366848 04
P00746	Complement factor D	0.3859667 76	0.0025440 65
P09172	Dopamine beta-hydroxylase	0.3865654 4	0.0159229 3
Q9P121	Neurotrimin	0.3881283 63	0.0142459 2
Q8NHP8	Putative phospholipase B-like 2 (LAMA-like protein 2)	0.3883594 2	0.0198979 15
P02765	Alpha-2-HS-glycoprotein	0.3894024 87	1.87645E-05
P06312	Immunoglobulin kappa variable 4-1	0.3900807 56	0.0027534 37
O75116	Rho-associated protein kinase 2	0.3901666	0.0209108 26
Q5T447	E3 ubiquitin-protein ligase HECTD3	0.3913285 1	0.0432237 83
P43251	Biotinidase	0.3931668 18	0.0010654 59
P02763	Alpha-1-acid glycoprotein 1	0.3940513 04	0.0006336 02
O15155	BET1 homolog (Golgi vesicular membrane-trafficking protein p18)	0.3966878 95	0.0364743 75
P07360	Complement component C8 gamma chain	0.3971286 59	0.0031409 19
Q92882	Osteoclast-stimulating factor 1	0.4006581 36	0.0195424 18
Q01968	Inositol polyphosphate 5-phosphatase OCRL	0.4010461 91	0.0073777 63
P51790	H(+)/Cl(-) exchange transporter 3 (Chloride transporter ClC-3)	0.4022012 72	0.0122251 17
Q70J99	Protein unc-13 homolog D	0.4041614 96	0.0348075 55
P35613	Basigin (Extracellular matrix metalloproteinase inducer)	0.4043194 28	0.0029049 39

P49961	Ectonucleoside triphosphate diphosphohydrolase 1	0.404589163	0.038349285
P55011	Solute carrier family 12 member 2	0.409101674	0.042651075
P02743	Serum amyloid P-component	0.409107375	0.030120909
P03952	Plasma kallikrein	0.409643888	6.67075E-05
Q8N128	Protein FAM177A1	0.410830408	0.00931409
P05154	Plasma serine protease inhibitor (Serpin A5)	0.412851738	0.000424782
P05155	Plasma protease C1 inhibitor	0.413735931	0.00421766
Q9BX68	Adenosine 5'-monophosphoramidase HINT2	0.413875901	0.034915022
P11021	Endoplasmic reticulum chaperone BiP (Heat shock protein 70 family protein 5)	0.414588991	0.000363003
P00740	Coagulation factor IX	0.414933824	0.001226601
Q14766	Latent-transforming growth factor beta-binding protein 1	0.416321941	0.038725079
P01024	Complement C3	0.416978436	1.25948E-05
P35916	Vascular endothelial growth factor receptor 3	0.419973853	0.002430586
Q9Y6G5	COMM domain-containing protein 10	0.420732269	0.042910267
P25311	Zinc-alpha-2-glycoprotein	0.423481605	0.002764262
Q9BVK6	Transmembrane emp24 domain-containing protein 9 (Glycoprotein 25L2)	0.423501249	0.04202402
P29353	SHC-transforming protein 1	0.42683245	0.012622226
P39060	Collagen alpha-1(XVIII) chain	0.42817824	0.026062142
P00736	Complement C1r subcomponent	0.428854552	0.042337418
Q08379	Golgin subfamily A member 2	0.430549297	0.028628578
Q9NYL9	Tropomodulin-3	0.432335572	0.007809492
Q9Y224	RNA transcription, translation and transport factor protein	0.432514021	0.039858708
Q9H8J5	MANSC domain-containing protein 1	0.433291466	0.008278381
Q96BS2	Calcineurin B homologous protein 3	0.434147499	0.00767633
P08697	Alpha-2-antiplasmin (Serpin F2)	0.434598929	9.24E-05
P46939	Utrophin (Dystrophin-related protein 1)	0.43526848	0.013614893
Q4VC31	Protein MIX23	0.43587266	0.04426666
Q06323	Proteasome activator complex subunit 1	0.436087421	0.007209671
Q15828	Cystatin-M	0.43739916	0.010868955
B9A064	Immunoglobulin lambda-like polypeptide 5	0.439538334	0.041489235
P80303	Nucleobindin-2	0.439841484	0.042337418
Q13464	Rho-associated protein kinase 1	0.440667387	0.033050793

P13804	Electron transfer flavoprotein subunit alpha, mitochondrial	0.441258876	0.03743508
O14744	Protein arginine N-methyltransferase 5 (Jak-binding protein 1)	0.441390396	0.026755577
P04843	Dolichyl-diphosphooligosaccharide–protein glycosyltransferase subunit 1 (Ribophorin I)	0.442264172	0.031868502
P22234	Bifunctional phosphoribosylaminoimidazole carboxylase/phosphoribosylaminoimidazole succinocarboxamide synthetase	0.443358048	0.033123725
Q8WZA0	Protein LZIC	0.445349981	0.025067051
P30049	ATP synthase F(1) complex subunit delta, mitochondrial	0.445591566	0.049573793
Q13449	Limbic system-associated membrane protein	0.446054414	0.030817552
P21796	Non-selective voltage-gated ion channel VDAC1	0.447571457	0.015632864
Q4KMQ2	Anoctamin-6 (SCAN channel)	0.447617163	0.039664218
Q96PD5	N-acetylmuramoyl-L-alanine amidase	0.44846473	0.000749734
P27824	Calnexin	0.449025476	0.032069072
P01031	Complement C5	0.450195965	0.001029923
P09493	Tropomyosin alpha-1 chain (Tropomyosin-1)	0.45208515	0.016101547
P02790	Hemopexin (Beta-1B-glycoprotein)	0.456198665	0.000504156
P02766	Transthyretin	0.456814924	0.00612461
Q6UWL2	Sushi domain-containing protein 1	0.457717458	0.042651075
P15144	Aminopeptidase N (CD antigen CD13)	0.458688163	0.014434978
P14151	L-selectin (CD62 antigen-like family member L, Leukocyte adhesion molecule 1)	0.459900657	0.001665671
P05156	Complement factor I	0.459959433	7.85543E-05
P17936	Insulin-like growth factor-binding protein 3	0.461380188	0.000224517
P00488	Coagulation factor XIII A chain	0.46545248	0.022614075
P26447	Protein S100-A4	0.468051084	0.046200986
P61604	10 kDa heat shock protein, mitochondrial	0.473483688	0.009451193
P01011	Alpha-1-antichymotrypsin	0.473853602	0.00416174
Q9NTJ5	Phosphatidylinositol-3-phosphatase SAC1	0.474525466	0.036500921
Q9UI30	Multifunctional methyltransferase subunit TRM112-like protein	0.476413097	0.046891468
P02748	Complement component C9	0.47718047	0.002317774
Q14624	Inter-alpha-trypsin inhibitor heavy chain H4	0.47765481	0.000367127
Q96RT1	Erbin	0.481520615	0.025618508
P52943	Cysteine-rich protein 2	0.482583538	0.008278381
Q12913	Receptor-type tyrosine-protein phosphatase eta	0.483932379	0.000489411
P02760	Protein AMBP	0.48546165	0.000104321

P16284	Platelet endothelial cell adhesion molecule	0.4856254 7	0.0258202 07
Q9NQS1	Cell death regulator Aven	0.4911705 44	0.0397919 26
P01034	Cystatin-C (Neuroendocrine basic polypeptide)	0.4911900 43	0.0102521 21
P00742	Coagulation factor X	0.4967576 44	0.0005531 37
Q6EMK4	Vasorin (Protein slit-like 2)	0.4992016 1	5.23453E-05

Table S3: Full list of identified upregulated DEPs in the SSc w/o ILD vs. CTRL contrast.

Accession (UNIPROT ID)	Name	Fold change	Adjusted p-value
Q9ULQ0	Striatin-interacting protein 2	23.932452 94	2.1051E-09
O60927	E3 ubiquitin-protein ligase PPP1R11 (Protein phosphatase inhibitor 3)	19.428440 81	3.23597E-07
Q8NHG8	E3 ubiquitin-protein ligase ZNRF2 (Protein Ells2)	14.842811 12	5.61536E-06
P38405	Guanine nucleotide-binding protein G(olf) subunit alpha	14.023832 96	2.21444E-09
P37198	Nuclear pore glycoprotein p62	13.411525 42	9.70748E-08
Q86SS6	Synaptotagmin-9	12.993487 61	3.99894E-08
Q9NPR2	Semaphorin-4B (Semaphorin-C)	11.615531 88	2.2456E-07
O60287	Nucleolar pre-ribosomal-associated protein 1	10.531046 86	0.0009267 5
P07766	T-cell surface glycoprotein CD3 epsilon chain	9.2733201 54	0.0020900 95
P15880	Small ribosomal subunit protein uS5	9.2314603 78	1.51151E-07
Q9Y333	U6 snRNA-associated Sm-like protein LSm2	7.9338025 6	0.0001514 08
P78417	Glutathione S-transferase omega-1	7.5710784 64	1.54844E-07
O43768	Alpha-endosulfine	7.4925129 44	1.3214E-07
Q9P0T7	Proton-transporting V-type ATPase complex assembly regulator TMEM9 (Transmembrane protein 9)	7.3882827 19	0.0008982 5
Q14667	Bridge-like lipid transfer protein family member 2	7.1695750 2	3.9082E-07
P13797	Plastin-3	7.0985337 78	2.7286E-11
Q99952	Tyrosine-protein phosphatase non-receptor type 18	6.9536360 36	2.93535E-06
Q96MW1	Coiled-coil domain-containing protein 43	6.9181665 85	0.0001331 24
Q9NRX4	14 kDa phosphohistidine phosphatase	6.7886311 49	0.0001072 43
Q8TB36	Ganglioside-induced differentiation-associated protein 1 (GDAP1)	6.6736897 7	8.91272E-06
Q9UN19	Dual adapter for phosphotyrosine and phosphoinositide	6.5971171 88	0.0001182 89
Q6P9F5	E3 ubiquitin ligase TRIM40	6.3750284 28	0.0366264 82
Q96M27	Protein PRRC1	6.2380535 59	5.25315E-06
P53004	Biliverdin reductase A	6.1923170 04	1.83145E-07

P14209	CD99 antigen (Protein MIC2)	5.920286708	4.55695E-10
Q9H3H3	UPF0696 protein C11orf68	5.881985472	0.003616607
Q9UBI6	Guanine nucleotide-binding protein G(I)/G(S)/G(O) subunit gamma-12	5.829544984	8.40661E-07
P05787	Keratin, type II cytoskeletal 8	5.701765007	0.000877119
O75367	Core histone macro-H2A.1	5.659329571	0.000452394
P16083	Ribosyldihydronicotinamide dehydrogenase [quinone]	5.618318772	0.000148973
O00244	Copper transport protein ATOX1	5.561882016	0.001825865
P62328	Thymosin beta-4	5.288065917	9.84146E-05
P06899	Histone H2B type 1-J	5.2568093	0.000813549
P26599	Polypyrimidine tract-binding protein 1	5.227397159	0.000814405
Q13336	Urea transporter 1	5.180239159	1.91627E-05
Q92890	Ubiquitin recognition factor in ER-associated degradation protein 1	5.154739864	0.00063975
Q05397	Focal adhesion kinase 1	5.150933842	0.001422065
P14550	Aldo-keto reductase family 1 member A1	5.150357956	0.002090095
Q3ZCW2	Galectin-related protein	5.095420728	4.23064E-05
P68431;Q71DI3	Histone H3.1	5.090783868	0.01876999
A9UHW6	MIF4G domain-containing protein	5.032403233	0.006425801
P06733	Alpha-enolase (Enolase 1)	4.921008715	6.79975E-11
P02538	Keratin, type II cytoskeletal 6A	4.910592038	0.008704785
Q96FS4	Signal-induced proliferation-associated protein 1 (Sipa-1)	4.841742622	3.99617E-06
Q8IV03	Leucine rich adaptor protein 1-like	4.810987311	0.002444619
P05388	Large ribosomal subunit protein uL10	4.682102179	9.5764E-06
P62942	Peptidyl-prolyl cis-trans isomerase FKBP1A	4.634596409	0.000139868
Q9NQ34	Transmembrane protein 9B	4.577542846	1.43663E-05
Q15046	Lysine–tRNA ligase	4.569463438	1.58318E-05
P24394	Interleukin-4 receptor subunit alpha	4.567985078	3.07435E-05
P62424	Large ribosomal subunit protein eL8	4.485672118	3.79921E-05
Q9BVJ7	Dual specificity protein phosphatase 23	4.484168314	0.000237778
Q9ULP9	TBC1 domain family member 24	4.424723852	0.005507664
P60900	Proteasome subunit alpha type-6	4.401879563	9.58716E-05
P19338	Nucleolin (Protein C23)	4.366647976	0.000104588
Q8TF64	PDZ domain-containing protein GIPC3	4.313522052	0.000239876

P17174	Aspartate aminotransferase, cytoplasmic	4.3114695 17	0.0005039 52
P16152	Carbonyl reductase [NADPH] 1	4.3090252 47	0.0004396 88
Q13574	Diacylglycerol kinase zeta	4.3034754 58	0.0019673 82
Q9BW62	Katanin p60 ATPase-containing subunit A-like 1	4.2879424 93	0.0012645 52
P63220	Small ribosomal subunit protein eS21	4.2728603 39	3.42819E-06
P13716	Delta-aminolevulinic acid dehydratase	4.2688377	0.0002434 18
P54277	PMS1 protein homolog 1	4.2667508 01	0.0110985 53
P49207	Large ribosomal subunit protein eL34	4.2411230 53	0.0034969 28
O95819	Mitogen-activated protein kinase kinase kinase kinase 4 (Nck-interacting kinase)	4.2361001 71	0.0016840 79
P41226	Ubiquitin-like modifier-activating enzyme 7	4.2240376 83	0.0142898 86
P25789	Proteasome subunit alpha type-4	4.1949719 58	0.0005713 1
P62906	Large ribosomal subunit protein uL1	4.1662688 05	1.51522E-08
Q07960	Rho GTPase-activating protein 1	4.1640206 14	3.75814E-06
P09211	Glutathione S-transferase P	4.1387747 35	0.0001764 35
P49768	Presenilin-1	4.0929477 98	0.0046209 41
P11172	Uridine 5'-monophosphate synthase (UMP synthase)	4.0874335 83	0.0027057 64
P61313	Large ribosomal subunit protein eL15	4.0690107 59	0.0004416 63
Q71RC9	Small integral membrane protein 5	4.0669764 13	0.0004500 66
P56385	ATP synthase F(0) complex subunit e, mitochondrial	4.0669010 2	4.23064E-05
Q03013	Glutathione S-transferase Mu 4	4.0221328 59	0.0067399 21
P16452	Protein 4.2 (Erythrocyte membrane protein band 4.2)	4.0055512 35	0.0009630 67
P37288	Vasopressin V1a receptor	3.9943886 78	0.0168553 66
P30273	High affinity immunoglobulin epsilon receptor subunit gamma	3.9911439 33	5.07527E-07
Q8TF74	WAS/WASL-interacting protein family member 2 (WIP-related protein)	3.9740486 82	4.02409E-05
Q9NR45	N-acetylneuraminate-9-phosphate synthase	3.9562359 73	0.0048419 3
Q9BZL6	Serine/threonine-protein kinase D2	3.9437767 78	0.0018284 14
P18621	Large ribosomal subunit protein uL22	3.9403927 97	0.0002721 22
Q9H6U6	BCAS3 microtubule associated cell migration factor	3.9326067 36	0.0082038 35
B2RUZ4	Small integral membrane protein 1	3.9297231	1.65245E-08
P80723	Brain acid soluble protein 1	3.9292602 89	1.85604E-08
Q9Y5X3	Sorting nexin-5	3.9233976 95	0.0006028 97
P78559	Microtubule-associated protein 1A	3.9058249 8	0.0158503 53

Q92734	Protein TFG	3.888675578	4.85395E-05
P00918	Carbonic anhydrase 2	3.888376299	0.000301796
Q9Y376	Calcium-binding protein 39	3.879310319	0.000459333
O95644	Nuclear factor of activated T-cells, cytoplasmic 1 (NF-ATc1)	3.87322774	0.016391701
P13798	Acylamino-acid-releasing enzyme	3.872279662	0.002572417
Q8N7R7	Cyclin-Y-like protein 1	3.861244845	0.001506555
Q5JQS6	Germinal centre-associated signalling and motility-like protein	3.85444967	0.00788748
Q96IU4	Putative protein-lysine deacylase ABHD14B	3.836421989	0.00123905
P54727	UV excision repair protein RAD23 homolog B	3.809119045	0.000148128
P60981	Destrin (Actin-depolymerizing factor)	3.808403326	0.000140016
Q9NRW4	Dual specificity protein phosphatase 22	3.805908682	0.003698994
P49721	Proteasome subunit beta type-2 (Macropain subunit C7-I)	3.792869419	0.002792503
P98170	E3 ubiquitin-protein ligase XIAP	3.790297313	0.004592406
P18077	Large ribosomal subunit protein eL33	3.789793764	0.001382134
P19971	Thymidine phosphorylase (Gliostatin)	3.783835648	0.002517282
P17538;Q6GPI1	Chymotrypsinogen B	3.756252581	0.003645144
Q6NZI2	Caveolae-associated protein 1 (Cavin-1)	3.756135834	0.000104113
Q15785	Mitochondrial import receptor subunit TOM34	3.751620069	0.003033159
O43237	Cytoplasmic dynein 1 light intermediate chain 2	3.712167052	0.015852363
Q8NFU3	Thiosulfate:glutathione sulfurtransferase	3.699953999	5.02862E-05
O95969	Secretoglobin family 1D member 2 (Lipophilin-B)	3.645126663	0.002147422
Q9NR56	Muscleblind-like protein 1	3.638454512	3.12054E-05
Q9NV70	Exocyst complex component 1	3.61329319	0.002595619
O00743	Serine/threonine-protein phosphatase 6 catalytic subunit	3.611185749	0.019255199
P29466	Caspase-1 (Interleukin-1 beta convertase)	3.59990025	4.25521E-05
Q15637	Splicing factor 1	3.590508914	3.79921E-05
Q9NP97	Dynein light chain roadblock-type 1	3.586393798	1.01081E-06
Q9UBN7	Protein deacetylase HDAC6	3.586376931	0.010276885
Q8WZ82	Esterase OVCA2	3.580052605	0.021059681
P30041	Peroxiredoxin-6	3.577767816	4.22371E-06
P30046	D-dopachrome decarboxylase	3.569059164	0.001438279
Q92997	Segment polarity protein dishevelled homolog DVL-3	3.561315994	0.029941801

Q13630	GDP-L-fucose synthase	3.515282026	0.009088851
P48444	Coatomer subunit delta	3.511977728	0.001264552
Q9H6Q3	Src-like-adapter 2	3.495054456	0.003553911
Q9H008	Phospholysine phosphohistidine inorganic pyrophosphate phosphatase	3.477412502	0.040561013
Q9NX14	NADH dehydrogenase [ubiquinone] 1 beta subcomplex subunit 11, mitochondrial	3.475897973	0.006327338
O15551	Claudin-3	3.450081378	0.013116645
P50995	Annexin A11	3.448946776	0.012767934
Q9BW04	Specifically androgen-regulated gene protein	3.447539942	0.020649185
Q15942	Zyxin	3.438125675	0.000139868
O75962	Triple functional domain protein(PTPRF-interacting protein)	3.436284014	0.028826895
Q8TDQ1	CMRF35-like molecule 1	3.420352612	0.021096464
P63272	Transcription elongation factor SPT4	3.40833587	0.019911358
Q9NR19	Acetyl-coenzyme A synthetase, cytoplasmic	3.397379373	0.001692348
Q02543	Large ribosomal subunit protein eL20	3.38264344	0.003133511
Q9H3S1	Semaphorin-4A (Semaphorin-B)	3.356321686	0.034628582
P25788	Proteasome subunit alpha type-3	3.352863472	0.005443831
P81605	Dermcidin	3.350059097	4.81241E-05
P36957	Dihydrolipoyllysine-residue succinyltransferase component of 2-oxoglutarate dehydrogenase complex, mitochondrial	3.337956869	0.000167687
P28070	Proteasome subunit beta type-4	3.308617518	0.009911282
Q8TCD5	5'(3')-deoxyribonucleotidase, cytosolic type	3.300165219	0.012350549
P42166	Lamina-associated polypeptide 2, isoform alpha	3.289634539	0.000131841
Q9Y316	Protein MEMO1	3.268469652	0.036055317
P26373	Large ribosomal subunit protein eL13	3.249532278	0.000815166
P51531	SWI/SNF-related matrix-associated actin-dependent regulator of chromatin subfamily A member 2 (SAMRCA2)	3.23814017	0.005087936
P04075	Fructose-bisphosphate aldolase A	3.235691342	3.49065E-07
Q96DR8	Mucin-like protein 1	3.23389869	0.00565484
Q96BJ3	Axin interactor, dorsalization-associated protein	3.226284271	0.011280356
P30047	GTP cyclohydrolase 1 feedback regulatory protein	3.218718466	0.00417118
Q9P2X3	Protein IMPACT	3.212564764	0.014974826
O95336	6-phosphogluconolactonase	3.210191199	0.004335554
P37108	Signal recognition particle 14 kDa protein (SRP14)	3.208892075	0.001212189
P48556	26S proteasome non-ATPase regulatory subunit 8	3.200564913	0.001524331

P43308	Translocon-associated protein subunit beta (TRAP-beta)	3.187919366	0.039343126
O75935	Dynactin subunit 3	3.185771676	0.026126721
P23588	Eukaryotic translation initiation factor 4B	3.181862426	0.003645144
O43175	D-3-phosphoglycerate dehydrogenase	3.124637335	0.001031052
Q16539	Mitogen-activated protein kinase 14 (MAP kinase 14)	3.116761909	0.027413009
Q15185	Prostaglandin E synthase 3	3.115118984	1.86454E-05
Q92686	Neurogranin	3.106589813	0.003830121
Q9NRR8	CDC42 small effector protein 1	3.085549971	0.000278588
O14713	Integrin beta-1-binding protein 1	3.073689134	0.042629507
Q8N392	Rho GTPase-activating protein 18	3.069210313	0.009016013
P08311	Cathepsin G	3.045328045	0.007507631
Q7KZF4	Staphylococcal nuclease domain-containing protein 1	3.04240905	0.026465769
P53367	Arfaptin-1 (ADP-ribosylation factor-interacting protein 1)	3.042062204	0.001531892
P20290	Transcription factor BTF3	3.039651962	2.38777E-05
O75871	Cell adhesion molecule CEACAM4	3.026746116	0.00123905
Q9UM22	Mammalian ependymin-related protein 1	3.025509132	0.010672057
Q13191	E3 ubiquitin-protein ligase CBL-B	3.021326358	0.004975513
Q9NRV9	Heme-binding protein 1	3.01274621	0.006855276
P27701	CD82 antigen (Tetraspanin-27)	3.009733796	0.009016013
P61081	NEDD8-conjugating enzyme Ubc12	3.003363256	1.07084E-05
P43405	Tyrosine-protein kinase SYK	3.001544583	0.00080348
P35236	Tyrosine-protein phosphatase non-receptor type 7	3.00125603	0.006824237
P62750	Large ribosomal subunit protein uL23	2.994615407	0.000109017
P42356	Phosphatidylinositol 4-kinase alpha	2.985564258	0.002966932
C9JLW8	Mapk-regulated corepressor-interacting protein 1 (Granulin-2)	2.979342073	0.00673987
Q7Z6I6	Rho GTPase-activating protein 30	2.972644285	0.001385533
P06744	Glucose-6-phosphate isomerase (Autocrine motility factor)	2.966488534	2.61713E-05
Q92608	Dedicator of cytokinesis protein 2	2.964949706	0.002033583
Q9Y336	Sialic acid-binding Ig-like lectin 9 (Siglec-9)	2.963985161	0.018075216
P06132	Uroporphyrinogen decarboxylase	2.95654084	0.007391055
P04899	Guanine nucleotide-binding protein G(i) subunit alpha-2	2.95507456	0.000325711
Q8WXI8	C-type lectin domain family 4 member D	2.944742743	0.012631232

Q9UGM3	Scavenger receptor cysteine-rich domain-containing protein DMBT1	2.943667077	0.012120293
Q8WWW8	GRB2-associated-binding protein 3	2.937869675	0.009870902
P61812	Transforming growth factor beta-2 proprotein (Cetermin)	2.935520041	0.012025733
Q7Z4W1	L-xylulose reductase	2.919415097	0.009653262
Q05823	2-5A-dependent ribonuclease	2.918719258	0.004352095
P21291	Cysteine and glycine-rich protein 1	2.910191044	0.000390697
Q9Y2D4	Exocyst complex component 6B	2.909129559	0.000222455
Q9HBL8	NmrA-like family domain-containing protein 1	2.902950431	0.016165526
Q9NRG0	Chromatin accessibility complex protein 1 (CHRAC-1)	2.901277708	0.000805922
Q9NRW7	Vacuolar protein sorting-associated protein 45	2.894074664	2.00419E-05
P59666;P59665	Neutrophil defensin 1/3	2.89078151	0.001385533
O00571	ATP-dependent RNA helicase DDX3X	2.879197947	0.001044785
P55795	Heterogeneous nuclear ribonucleoprotein H2	2.874285534	0.002437013
P19623	Spermidine synthase	2.856711213	0.004637278
O75190	DnaJ homolog subfamily B member 6	2.847874778	0.015916414
Q7Z422	SUZ RNA-binding domain-containing protein	2.842657991	0.00174718
Q99879	Histone H2B type 1-M	2.842233499	0.033504697
O15263	Defensin beta 4A	2.838670602	0.047454451
P36551	Oxygen-dependent coproporphyrinogen-III oxidase, mitochondrial	2.837281541	0.023323256
P60842	Eukaryotic initiation factor 4A-I	2.823667819	0.010449473
P46776	Large ribosomal subunit protein uL15	2.822217179	0.00172766
P08708	Small ribosomal subunit protein eS17	2.804544664	0.000112177
O75083	WD repeat-containing protein 1 (Actin-interacting protein 1)	2.803345973	0.009870902
P46779	Large ribosomal subunit protein eL28	2.801218548	0.002218661
P51452	Dual specificity protein phosphatase 3	2.798370864	0.01189449
P62280	Small ribosomal subunit protein uS17	2.790496984	0.004695105
Q9UMR7	C-type lectin domain family 4 member A	2.784081297	0.03475221
P61247	Small ribosomal subunit protein eS1	2.779831305	0.000503952
P12724	Eosinophil cationic protein	2.774262761	0.030959301
O95685	Protein phosphatase 1 regulatory subunit 3D	2.769932366	0.048345231
Q86YZ3	Hornerin	2.76568689	0.018511378
Q99719	Septin-5	2.765018518	0.009706989

P21731	Thromboxane A2 receptor	2.75508385	0.012025733
P48507	Glutamate–cysteine ligase regulatory subunit	2.745238742	0.000199406
Q8NEU8	DCC-interacting protein 13-beta	2.742997146	0.023214117
Q12882	Dihydropyrimidine dehydrogenase [NADP(+)]	2.740081292	0.018632257
P61088	Ubiquitin-conjugating enzyme E2 N	2.73814712	0.021302282
O43353	Receptor-interacting serine/threonine-protein kinase 2	2.732086568	0.018767681
P31943	Heterogeneous nuclear ribonucleoprotein H	2.724682536	1.7979E-05
P62081	Small ribosomal subunit protein eS7	2.712523794	0.000168672
Q9H1K6	Talin rod domain-containing protein 1	2.690741254	0.006848778
Q9BWD1	Acetyl-CoA acetyltransferase, cytosolic	2.690500413	0.012825062
P62241	Small ribosomal subunit protein eS8	2.683879425	0.001351775
P52597	Heterogeneous nuclear ribonucleoprotein F	2.677002961	0.0007703
O14595	Carboxy-terminal domain RNA polymerase II polypeptide A small phosphatase 2 (Protein OS-4)	2.656702456	0.001162769
Q96JY6	PDZ and LIM domain protein 2	2.656030123	0.000536677
Q5MNZ9	WD repeat domain phosphoinositide-interacting protein 1	2.64524198	0.049573267
P32969	Large ribosomal subunit protein uL6	2.639586178	0.039078294
Q9UBW5	Bridging integrator 2 (Breast cancer-associated protein 1)	2.616372124	3.78313E-05
Q92530	Proteasome inhibitor PI31 subunit	2.61261784	0.030406111
O00330	Pyruvate dehydrogenase protein X component, mitochondrial (E3-binding protein)	2.609012509	0.043277133
P30566	Adenylosuccinate lyase (ADSL)	2.606082306	0.013116645
O43182	Rho GTPase-activating protein 6	2.604914883	0.001136762
P50552	Vasodilator-stimulated phosphoprotein (VASP)	2.599607365	0.000119506
P14174	Macrophage migration inhibitory factor	2.59935277	0.000140371
Q8IXJ6	NAD-dependent protein deacetylase sirtuin-2	2.586254017	0.028781022
Q96K21	Abscission/NoCut checkpoint regulator	2.5775609	0.035621403
P01116	GTPase KRas	2.573372189	0.003080629
Q8IZ83	Aldehyde dehydrogenase family 16 member A1	2.57202478	0.035367079
O14818	Proteasome subunit alpha type-7	2.568855318	0.010769455
Q92625	Ankyrin repeat and SAM domain-containing protein 1A (Odin)	2.565718064	0.042037501
O43516	WAS/WASL-interacting protein family member 1	2.548791056	0.000123696
P13639	Elongation factor 2	2.545604489	0.000721907
P61981	14-3-3 protein gamma	2.534338299	0.006979935

P49411	Elongation factor Tu, mitochondrial	2.53188564	0.000693888
Q7L7X3	Serine/threonine-protein kinase TAO1	2.524135477	0.016981528
P37837	Transaldolase	2.523417504	4.41273E-05
O95833	Chloride intracellular channel protein 3	2.521549695	0.040400161
Q9Y2Z0	Protein SGT1 homolog	2.513009193	0.002549289
O14908	PDZ domain-containing protein GIPC1	2.50810142	0.007425659
P40121	Macrophage-capping protein (Actin regulatory protein CAP-G)	2.50538584	0.005029534
P56749	Claudin-12	2.489088116	0.012266009
Q9BQ89	Protein FAM110A	2.48356689	0.026638193
Q9NTK5	Obg-like ATPase 1	2.480317051	0.000709866
Q9BYM8	RanBP-type and C3HC4-type zinc finger-containing protein 1 (HBV-associated factor 4)	2.474304591	0.008146985
Q13347	Eukaryotic translation initiation factor 3 subunit I (TGF-beta receptor-interacting protein 1)	2.468677351	0.01958089
Q86VP6	Cullin-associated NEDD8-dissociated protein 1	2.467472812	0.026182043
Q9H6T3	RNA polymerase II-associated protein 3	2.460837077	0.009870902
P09543	2',3'-cyclic-nucleotide 3'-phosphodiesterase	2.460700625	0.001509264
O75521	Enoyl-CoA delta isomerase 2	2.439070079	0.025541787
P62834	Ras-related protein Rap-1A	2.438610738	0.006743319
Q9BUH6	Protein PAXX	2.430904301	0.000441663
Q04637	Eukaryotic translation initiation factor 4 gamma 1	2.430062587	0.020697577
P53999	Activated RNA polymerase II transcriptional coactivator p15 (Positive cofactor 4)	2.423412135	0.0114716
P37840	Alpha-synuclein	2.422101875	5.13568E-05
Q02878	Large ribosomal subunit protein eL6	2.411374021	0.005199499
P09651	Heterogeneous nuclear ribonucleoprotein A1	2.398977563	0.005071647
Q8ND76	Cyclin-Y	2.395060025	0.013116645
P49795	Regulator of G-protein signalling 19	2.388877603	0.001461039
Q92558	Actin-binding protein WASF1 (Protein WAVE-1)	2.385391799	0.021054045
O60493	Sorting nexin-3	2.381660714	0.000421775
A6NI79	Coiled-coil domain-containing protein 69	2.36798948	0.034307981
P34897	Serine hydroxymethyltransferase, mitochondrial	2.350146823	0.015017811
Q9H223	EH domain-containing protein 4 (Hepatocellular carcinoma-associated protein 10/11)	2.343669401	0.002512372
Q96P48	Arf-GAP with Rho-GAP domain, ANK repeat and PH domain-containing protein 1	2.335143798	0.015173355
Q96BY6	Dedicator of cytokinesis protein 10	2.333048383	0.007775401

O15027	Protein transport protein Sec16A	2.328287745	0.010928919
Q15758	Neutral amino acid transporter B(0)	2.321729017	0.000180274
P51148	Ras-related protein Rab-5C	2.317938184	0.004352095
Q9NUU7;Q9UMR2	ATP-dependent RNA helicase DDX19A	2.313053634	0.018632257
Q15056	Eukaryotic translation initiation factor 4H	2.306692352	0.007554608
O60749	Sorting nexin-2	2.303161409	0.030059347
Q9NP79	Vacuolar protein sorting-associated protein VTA1 homolog	2.301403782	0.032439741
Q3YEC7	Rab-like protein 6	2.300303343	0.026291567
Q9H0E2	Toll-interacting protein	2.295321228	7.93533E-05
Q6P9G4	Transmembrane protein 154	2.291863775	0.03448413
O43312	Protein MTSS1 (Metastasis suppressor YGL-1)	2.28501577	0.008864676
P31431	Syndecan-4	2.28483901	0.046011115
Q9Y3C5	RING finger protein 11	2.284563089	0.000496023
P09564	T-cell antigen CD7	2.28277246	0.040287932
Q96AT9	Ribulose-phosphate 3-epimerase	2.279357467	0.002212341
Q9UKK9	ADP-sugar pyrophosphatase	2.266155056	0.031926257
P07195	L-lactate dehydrogenase B chain	2.260982877	7.55843E-05
Q9UKY7	Protein CDV3 homolog	2.258192718	8.88203E-05
Q6ICL3	Transport and Golgi organization protein 2 homolog	2.257948694	0.037411584
Q9NSD9	Phenylalanine–tRNA ligase beta subunit	2.248011057	0.004902387
O95810	Caveolae-associated protein 2 (Cavin-2)	2.235897934	0.025541787
P61106	Ras-related protein Rab-14	2.23443462	0.009213503
Q96IG2	F-box/LRR-repeat protein 20	2.228664207	0.047250025
Q8N434	Putative transporter SVOPL	2.225316185	0.000125903
O43396	Thioredoxin-like protein 1	2.22327095	0.002300372
Q9BY44	Eukaryotic translation initiation factor 2A	2.206176716	0.019346429
Q5JWF2	Guanine nucleotide-binding protein G(s) subunit alpha isoforms XLas	2.204449425	0.02388719
P06241	Tyrosine-protein kinase Fyn (Proto-oncogene Syn)	2.203181817	0.023630956
P78344	Eukaryotic translation initiation factor 4 gamma 2	2.203096324	0.022319843
Q9NZL9	Methionine adenosyltransferase 2 subunit beta	2.20265813	0.003698994
P26641	Elongation factor 1-gamma	2.200672752	0.000350885
Q9H299	SH3 domain-binding glutamic acid-rich-like protein 3 (TNF inhibitory protein B1)	2.194173	0.024119378

P46783	Small ribosomal subunit protein eS10	2.192132616	0.00456697
P55196	Afadin	2.189010292	0.022967908
P31025;Q5VSP4	Lipocalin-1	2.183203	0.010769455
Q15126	Phosphomevalonate kinase	2.182388184	0.018395594
Q8IUW5	RELT-like protein 1	2.175807957	0.009745779
O43914	TYRO protein tyrosine kinase-binding protein	2.175545659	0.002943807
P62917	Large ribosomal subunit protein uL2	2.175047423	0.021688702
P05089	Arginase-1 (Liver-type arginase)	2.167905145	0.046699383
Q8IYJ3	Synaptotagmin-like protein 1 (Exophilin-7)	2.161349511	0.012767934
P29966	Myristoylated alanine-rich C-kinase substrate	2.15514276	0.000134689
Q15746	Myosin light chain kinase, smooth muscle	2.148865957	0.006779123
O43432	Eukaryotic translation initiation factor 4 gamma 3	2.143649454	0.041824118
Q5VVQ6	Ubiquitin thioesterase OTU1	2.137394536	0.04134592
P12081	Histidine–tRNA ligase, cytoplasmic	2.130109443	0.01042095
Q9GZU7	Carboxy-terminal domain RNA polymerase II polypeptide A small phosphatase 1	2.129644618	0.000789031
P62491;Q15907	Ras-related protein Rab-11A	2.126691916	0.040994731
P06729	T-cell surface antigen CD2	2.125151629	0.0125102
Q96I24	Far upstream element-binding protein 3 (FUSE-binding protein 3)	2.118252514	0.0323583
Q765P7	Protein MTSS2	2.097216994	0.044642534
O00299	Chloride intracellular channel protein 1	2.090380964	0.001031052
Q04759	Protein kinase C theta type	2.084823019	0.012731879
P04083	Annexin A1	2.082996363	0.031422092
P27448	MAP/microtubule affinity-regulating kinase 3 (C-TAK1)	2.081265311	0.033540113
Q99490	Arf-GAP with GTPase, ANK repeat and PH domain-containing protein 2 (Centaurin-gamma-1)	2.081247763	0.037563943
B0I1T2	Unconventional myosin-Ig	2.080425972	0.003151226
O95319	CUGBP Elav-like family member 2 (CELF-2)	2.080273141	0.049223534
Q562R1	Beta-actin-like protein 2 (Kappa-actin)	2.080048215	0.029484101
O60234	Glia maturation factor gamma	2.076342709	0.013116645
Q9Y287	Integral membrane protein 2B	2.076259006	0.019296907
P15391	B-lymphocyte antigen CD19	2.075401397	0.012172962
Q7Z4H3	5'-deoxynucleotidase HDDC2	2.072656405	0.000265725
Q9Y3E2	BolA-like protein 1	2.070230337	0.008883044

P62993	Growth factor receptor-bound protein 2	2.066000419	0.013803219
P07305	Histone H1.0	2.064997504	0.030501992
P11940	Polyadenylate-binding protein 1	2.0610111	0.01501699
Q9NZ32	Actin-related protein 10	2.059886087	0.035367079
Q9HB71	Calcyclin-binding protein	2.059459867	0.00484193
O95372	Acyl-protein thioesterase 2 (Lysophospholipase II)	2.05797091	0.011444667
Q9H4A3	Serine/threonine-protein kinase WNK1	2.056919304	0.034298153
P22392	Nucleoside diphosphate kinase B	2.05255745	0.008984928
Q9NY25	C-type lectin domain family 5 member A	2.045997362	0.03287001
P62249	Small ribosomal subunit protein uS9	2.043413696	0.026182043
P11586	C-1-tetrahydrofolate synthase, cytoplasmic	2.037883941	0.014289886
Q8IZP0	Abl interactor 1 (Nap1-binding protein)	2.037257189	0.007384711
P62263	Small ribosomal subunit protein uS11	2.034663819	0.002790634
Q96NA2	Rab-interacting lysosomal protein	2.030293398	0.013116645
Q9BV68	E3 ubiquitin-protein ligase RNF126	2.025555618	0.03336905
Q5HYW2	NHS-like protein 2	2.010325883	0.012767934
Q8N699	Myc target protein 1	2.00727962	0.000788175
P24666	Low molecular weight phosphotyrosine protein phosphatase	2.000118889	0.014373848

Table S4: Full list of identified downregulated DEPs in the SSc w/o ILD vs. CTRL contrast.

Accession (UNIPROT ID)	Name	Fold change	Adjusted p-value
P18428	Lipopolysaccharide-binding protein	0.036672562	2.70586E-28
P01706	Immunoglobulin lambda variable 2-11	0.051992651	1.18711E-08
Q6UXB8	Peptidase inhibitor 16 (Cysteine-rich secretory protein 9, CRISP-9)	0.063793375	4.68391E-14
P01709	Immunoglobulin lambda variable 2-8	0.064776155	1.45991E-10
A0A075B7D0	Immunoglobulin heavy variable 1/OR15-1	0.064831421	8.07474E-07
Q9Y5Y7	Lymphatic vessel endothelial hyaluronic acid receptor 1 (LYVE-1)	0.065683461	1.00064E-25
P51572	B-cell receptor-associated protein 31	0.065698529	2.24813E-07
P23470	Receptor-type tyrosine-protein phosphatase gamma	0.066267881	6.87916E-16
Q96KN2	Beta-Ala-His dipeptidase	0.076731962	3.17469E-14
P05546	Heparin cofactor 2	0.08276003	1.12134E-10
P05976	Myosin light chain 1/3, skeletal muscle isoform	0.083282479	0.000830285

Q9NP78	ABC-type oligopeptide transporter ABCB9	0.085066002	9.13521E-05
A0A075B6I1	Immunoglobulin lambda variable 4-60	0.088296512	0.003144152
O75144	ICOS ligand	0.09036279	1.37179E-16
P19652	Alpha-1-acid glycoprotein 2 (Orosomucoid-2)	0.091285018	6.19098E-11
P13591	Neural cell adhesion molecule 1	0.097334213	2.1088E-11
P22891	Vitamin K-dependent protein Z	0.097568465	5.75664E-09
Q14165	Malectin	0.098241368	1.6739E-10
A0A0A0MS15	Immunoglobulin heavy variable 3-49	0.098576793	3.5261E-10
Q7Z3E2	Coiled-coil domain-containing protein 186	0.108723603	0.000554537
Q9UNN8	Endothelial protein C receptor	0.116144177	8.4329E-10
P07360	Complement component C8 gamma chain	0.11628875	2.75303E-14
Q9HCN8	Stromal cell-derived factor 2-like protein 1	0.117359606	2.47174E-06
P06276	Cholinesterase	0.118506347	8.4329E-10
P41222	Prostaglandin-H2 D-isomerase	0.120747766	1.90337E-10
Q14314	Fibroleukin	0.125998983	1.90337E-10
P55058	Phospholipid transfer protein	0.126707153	4.68391E-14
P04180	Phosphatidylcholine-sterol acyltransferase	0.128051045	2.86228E-12
P54289	Voltage-dependent calcium channel subunit alpha-2/delta-1	0.132784685	1.24339E-09
P20851	C4b-binding protein beta chain	0.135612824	1.50785E-11
Q9HDC9	Adipocyte plasma membrane-associated protein	0.140647968	2.32581E-08
Q8TER0	Sushi, nidogen and EGF-like domain-containing protein 1	0.142399002	0.000102147
P35858	Insulin-like growth factor-binding protein complex acid labile subunit	0.147091235	2.7286E-11
Q8TDY8	Immunoglobulin superfamily DCC subclass member 4	0.147131011	0.000115197
Q86X83	COMM domain-containing protein 2	0.148320874	1.14234E-05
P33151	Cadherin-5	0.1494912	2.29478E-06
Q13449	Limbic system-associated membrane protein	0.1495913	5.47707E-10
P0C0L5	Complement C4-B	0.150997238	5.26374E-16
P0C0L4	Complement C4-A	0.151051883	5.22744E-07
P30519	Heme oxygenase 2	0.151389648	0.000259343
O00391	Sulfhydryl oxidase 1	0.153584034	1.91658E-12
Q9UBV8	Peflin	0.159324246	8.20733E-06
Q86UN3	Reticulon-4 receptor-like 2	0.159899506	1.56344E-11

P06681	Complement C2 (C3/C5 convertase)	0.161148715	9.20202E-21
O60613	Selenoprotein F	0.161212476	0.00123905
P52815	Large ribosomal subunit protein bL12m	0.165004293	1.56141E-05
A0A0B4J1Y9	Immunoglobulin heavy variable 3-72	0.165835909	4.01854E-07
P63267	Actin, gamma-enteric smooth muscle	0.165876129	7.55843E-05
Q96PC5	Melanoma inhibitory activity protein 2	0.166290492	0.001181421
P00450	Ceruloplasmin	0.166717475	7.23392E-12
O60279	Sushi domain-containing protein 5	0.167800726	0.000110966
P43652	Afamin (Alpha-albumin)	0.168070979	1.44974E-13
P15954	Cytochrome c oxidase subunit 7C, mitochondrial	0.169178574	0.00045775
A0A075B6I4	Immunoglobulin lambda variable 10-54	0.171684503	0.006971399
P19022	Cadherin-2	0.171858001	5.20903E-12
P20036	HLA class II histocompatibility antigen, DP alpha 1 chain	0.173650034	1.34229E-06
P51153	Ras-related protein Rab-13	0.174732312	2.33097E-06
P14927	Cytochrome b-c1 complex subunit 7	0.174801713	8.13629E-06
Q9Y6D6	Brefeldin A-inhibited guanine nucleotide-exchange protein 1	0.177092082	1.92776E-05
Q76LX8	A disintegrin and metalloproteinase with thrombospondin motifs 13 (ADAMTS-13)	0.177157455	1.06695E-06
P07357	Complement component C8 alpha chain	0.177320274	4.3505E-13
P35052	Glypican-1	0.178312283	0.001218634
Q92823	Neuronal cell adhesion molecule	0.1784862	0.000733971
P00387	NADH-cytochrome b5 reductase 3	0.178835156	1.36198E-06
Q8WTV0	Scavenger receptor class B member 1	0.179814906	0.000610516
O00533	Neural cell adhesion molecule L1-like protein	0.180297271	2.75303E-14
A0A0J9YY99	Ig-like domain-containing protein	0.180413036	4.53066E-08
Q15293	Reticulocalbin-1	0.180553954	0.000569253
P51884	Lumican	0.180638142	4.68391E-14
Q92747	Actin-related protein 2/3 complex subunit 1A	0.18116737	0.008895163
P01704	Immunoglobulin lambda variable 2-14	0.181170474	0.043277133
P19823	Inter-alpha-trypsin inhibitor heavy chain H2	0.183733003	3.17469E-14
Q14515	SPARC-like protein 1 (Hevin)	0.185130502	1.94E-09
P18850	Cyclic AMP-dependent transcription factor ATF-6 alpha	0.186854066	1.08599E-08
P05452	Tetranectin	0.187244971	2.54232E-12

P04003	C4b-binding protein alpha chain	0.188947109	1.87879E-11
P01019	Angiotensinogen	0.190093318	2.98915E-11
Q14392	Transforming growth factor beta activator LRRC32 (Garpin)	0.191272	8.23509E-05
Q9UBX5	Fibulin-5	0.192354486	0.006129686
Q9NZA1	Chloride intracellular channel protein 5 (Glutaredoxin-like oxidoreductase CLIC5)	0.194521651	6.41401E-05
P00746	Complement factor D	0.197251922	8.4329E-10
Q14126	Desmoglein-2 (Cadherin family member 5)	0.19749002	2.98058E-08
P11047	Laminin subunit gamma-1	0.198921344	0.000218405
Q9P121	Neurotrimin	0.199005724	1.18642E-06
P15151	Poliovirus receptor (Nectin-like protein 5)	0.202308788	3.44659E-08
P12821	Angiotensin-converting enzyme	0.202885701	0.000264798
P00734	Prothrombin	0.202975174	4.68664E-10
P01857	Immunoglobulin heavy constant gamma 1	0.205365595	5.57301E-11
Q9NZP8	Complement C1r subcomponent-like protein	0.205376717	3.17469E-14
P02750	Leucine-rich alpha-2-glycoprotein	0.205821937	3.72199E-10
P02766	Transthyretin	0.207337569	7.25672E-11
P08185	Corticosteroid-binding globulin (Serpin A6)	0.210311311	3.38735E-14
Q92496	Complement factor H-related protein 4	0.211516721	3.7328E-06
P20674	Cytochrome c oxidase subunit 5A, mitochondrial	0.211702604	0.000109635
A0A075B7B8	Immunoglobulin heavy variable 3/OR16-12	0.212413038	0.000428186
O43169	Cytochrome b5 type B	0.213829214	0.005196881
P05160	Coagulation factor XIII B chain	0.214614656	1.64569E-06
Q9NVI7;Q5T9A4	ATPase family AAA domain-containing protein 3A	0.214685362	0.000446126
Q08380	Galectin-3-binding protein	0.216223982	0.000109635
P00739	Haptoglobin-related protein	0.21741265	2.76541E-06
P08603	Complement factor H	0.218388328	7.04252E-11
P22792	Carboxypeptidase N subunit 2	0.219407281	1.0206E-11
P01031	Complement C5	0.219833598	1.60414E-12
O00585	C-C motif chemokine 21	0.222173096	6.6104E-06
P07225	Vitamin K-dependent protein S	0.22230979	1.04494E-12
O75146	Huntingtin-interacting protein 1-related protein	0.222968524	0.000805922
P15586	N-acetylglucosamine-6-sulfatase	0.223638331	0.00025842

P01009	Alpha-1-antitrypsin	0.225774419	2.21253E-13
P04179	Superoxide dismutase [Mn], mitochondrial	0.226078836	7.53813E-06
P11177	Pyruvate dehydrogenase E1 component subunit beta, mitochondrial	0.226333666	0.001309422
P19827	Inter-alpha-trypsin inhibitor heavy chain H1	0.227572161	1.53106E-12
O75882	Attractin (DPPT-L)	0.227893113	1.73887E-12
Q8NHP8	Putative phospholipase B-like 2	0.228218465	1.42042E-05
P20742	Pregnancy zone protein	0.228433788	0.008111161
Q86TH1	ADAMTS-like protein 2	0.22914405	0.000291942
Q9Y6R7	IgGFc-binding protein	0.229208699	6.65021E-09
A0A0A0MT36	Immunoglobulin kappa variable 6D-21	0.232467539	0.001988104
Q9Y646	Carboxypeptidase Q	0.232971032	5.33613E-09
Q9Y240	C-type lectin domain family 11 member A	0.233111936	0.000247721
P19320	Vascular cell adhesion protein 1	0.233597574	2.76846E-09
Q9UK55	Protein Z-dependent protease inhibitor	0.234290963	5.57604E-12
A0A0B4J1X5	Immunoglobulin heavy variable 3-74	0.234947069	0.000721907
P0DP08	Immunoglobulin heavy variable 4-38-2	0.234976525	0.000501951
Q9NZF1	Placenta-specific gene 8 protein	0.235644298	0.000503952
P02763	Alpha-1-acid glycoprotein 1 (Orosomucoid-1)	0.23569135	4.8655E-10
O60437	Periplakin	0.237418945	0.001170103
Q6ZNJ1	Neurobeachin-like protein 2	0.237648875	0.00101081
Q02985	Complement factor H-related protein 3	0.238631897	0.008904245
P01024	Complement C3	0.239472717	1.45649E-14
Q9H2M9	Rab3 GTPase-activating protein non-catalytic subunit	0.239758294	0.005241512
P04278	Sex hormone-binding globulin	0.239896885	7.55843E-05
Q14789	Golgin subfamily B member 1	0.240226264	0.008254412
Q13641	Trophoblast glycoprotein	0.241978926	0.000823124
Q8N4L2	Type 2 phosphatidylinositol 4,5-bisphosphate 4-phosphatase (Transmembrane protein 55A)	0.242159938	0.000602897
P35916	Vascular endothelial growth factor receptor 3	0.245457043	3.4673E-09
P01780	Immunoglobulin heavy variable 3-7	0.246115961	1.96641E-08
P54108	Cysteine-rich secretory protein 3 (CRISP-3)	0.246321983	0.000181564
Q16610	Extracellular matrix protein 1	0.246530767	0.000450066
Q92614	Unconventional myosin-XVIIIa (Molecule associated with JAK3 N-terminus)	0.247072608	0.0007703

P30825	High affinity cationic amino acid transporter 1	0.248139491	0.000258246
B9A064	Immunoglobulin lambda-like polypeptide 5	0.249769211	1.37053E-05
Q96A33	PAT complex subunit CCDC47 (Calumin)	0.251216782	0.030882946
Q9Y608	Leucine-rich repeat flightless-interacting protein 2 (LRR FLII-interacting protein 2)	0.251561772	0.00010253
P11226	Mannose-binding protein C	0.251568826	0.002792503
O75396	Vesicle-trafficking protein SEC22b	0.251832404	0.005682582
P01703	Immunoglobulin lambda variable 1-40	0.255070517	4.41273E-05
P25311	Zinc-alpha-2-glycoprotein	0.255364172	1.04781E-08
Q9Y2Q3	Glutathione S-transferase kappa 1	0.255825098	0.009016013
P02748	Complement component C9	0.256291869	4.83142E-11
O15247	Chloride intracellular channel protein 2 (Glutaredoxin-like oxidoreductase CLIC2)	0.256411979	0.000303894
P13611	Versican core protein	0.256977884	0.000186285
A0A0A0MT96	Immunoglobulin kappa joining 3	0.257107875	1.63248E-05
Q99653	Calcineurin B homologous protein 1	0.258335554	4.25521E-05
Q15389	Angiopoietin-1	0.258894145	0.002697759
P00751	Complement factor B	0.259280897	6.36393E-09
P01008	Antithrombin-III	0.261227612	1.31903E-15
Q8IUL8	Cartilage intermediate layer protein 2	0.262672	3.60329E-06
Q16627	C-C motif chemokine 14	0.263149663	6.75934E-05
A0A0C4DH55	Immunoglobulin kappa variable 3D-7	0.263459742	0.035766945
P00736	Complement C1r subcomponent	0.264104348	5.09464E-05
Q9UBI1	COMM domain-containing protein 3	0.2651505	0.00198119
P22234	Bifunctional phosphoribosylaminoimidazole carboxylase/phosphoribosylaminoimidazole succinocarboxamide synthetase (PAICS)	0.265201311	2.86588E-05
P06858	Lipoprotein lipase	0.265501187	3.01138E-05
P12110	Collagen alpha-2(VI) chain	0.266158694	3.98506E-06
P01861	Immunoglobulin heavy constant gamma 4	0.266288428	0.009939506
P19404	NADH dehydrogenase [ubiquinone] flavoprotein 2, mitochondrial	0.266993993	0.000838639
P04070	Vitamin K-dependent protein C	0.270911494	2.41004E-08
P36955	Pigment epithelium-derived factor	0.274142526	1.02943E-07
Q9BRT2	Ubiquinol-cytochrome c reductase complex assembly factor 2	0.274406903	0.00432978
P02765	Alpha-2-HS-glycoprotein	0.275525828	2.21295E-11
O00291	Huntingtin-interacting protein 1	0.275868118	0.002966932

P02753	Retinol-binding protein 4	0.276307768	0.000236237
Q13740	CD166 antigen (Activated leukocyte cell adhesion molecule)	0.277463729	2.28032E-09
Q86UP2	Kinectin	0.277505534	0.000116805
P01893	Putative HLA class I histocompatibility antigen, alpha chain H	0.277715571	0.013173906
Q9UEW3	Macrophage receptor MARCO	0.278372435	0.003453447
Q8N2S1	Latent-transforming growth factor beta-binding protein 4	0.278374956	0.013338742
P35579	Myosin-9	0.280188649	3.76214E-05
Q9UL25	Ras-related protein Rab-21	0.282304759	0.003773365
Q9NRL3	Striatin-4	0.283280331	0.000717182
Q9UBG0	C-type mannose receptor 2	0.283697839	3.98506E-06
Q15582	Transforming growth factor-beta-induced protein ig-h3	0.284703605	1.18887E-07
P14151	L-selectin (Leukocyte adhesion molecule 1)	0.285710817	1.50515E-09
P01715	Immunoglobulin lambda variable 3-1	0.286794594	0.005884649
P60660	Myosin light polypeptide 6	0.287606447	0.000711256
P51790	H(+)/Cl(-) exchange transporter 3	0.28807872	4.1115E-05
Q13201	Multimerin-1 (EMILIN-4)	0.288896037	3.01138E-05
P02790	Hemopexin (Beta-1B-glycoprotein)	0.289092415	1.43838E-10
P26842	CD27 antigen (Tumour necrosis factor receptor superfamily member 7)	0.28950242	0.012937425
P02751	Fibronectin	0.289864121	0.00474016
P00740	Coagulation factor IX	0.290014941	4.24996E-08
Q9BWM7	Sideroflexin-3	0.290404295	0.001538774
O75947	ATP synthase peripheral stalk subunit d, mitochondrial	0.291036996	0.000127882
A0A075B6I9	Immunoglobulin lambda variable 7-46	0.291639149	6.33788E-05
O00625	Pirin	0.292025495	0.000720761
Q15223	Nectin-1	0.292317002	0.000602897
P26927	Hepatocyte growth factor-like protein (Macrophage stimulatory protein)	0.292901857	4.26105E-06
Q9NY15	Stabilin-1	0.293477926	6.44653E-06
P01034	Cystatin-C	0.293698659	5.80926E-08
P61586	Transforming protein RhoA	0.294059191	0.000351477
Q9NX76	CKLF-like MARVEL transmembrane domain-containing protein 6	0.294302553	0.039078294
Q9UHL4	Dipeptidyl peptidase 2	0.294434671	0.001316649
Q9NTJ5	Phosphatidylinositol-3-phosphatase SAC1	0.294553083	3.36249E-05

P08236	Beta-glucuronidase	0.295117492	0.003140735
Q9Y4D8	Probable E3 ubiquitin-protein ligase HECTD4	0.2956854	0.008203835
Q02818	Nucleobindin-1 (CALNUC)	0.296675655	2.84856E-05
Q9UN36	Protein NDRG2	0.297420546	0.002818747
Q00325	Solute carrier family 25 member 3	0.29760613	0.017528466
P49961	Ectonucleoside triphosphate diphosphohydrolase 1	0.300684549	0.000623259
Q7Z7G0	Target of Nesh-SH3	0.302318494	0.004705674
Q14BN4	Sarcolemmal membrane-associated protein	0.302908447	0.000581575
Q9NPY3	Complement component C1q receptor	0.303301066	0.006226734
Q15075	Early endosome antigen 1	0.303960295	0.000154627
Q93033	Immunoglobulin superfamily member 2	0.304825622	0.025436182
P12109	Collagen alpha-1(VI) chain	0.305296022	0.000735682
Q70J99	Protein unc-13 homolog D	0.305406727	0.000602183
Q9BRV8	Suppressor of IKBKE 1	0.305419286	0.006739921
P03952	Plasma kallikrein	0.305603138	6.16201E-10
Q92581	Sodium/hydrogen exchanger 6	0.305840098	0.00172766
P01023	Alpha-2-macroglobulin	0.306124324	1.09777E-08
O15212	Prefoldin subunit 6	0.308258286	0.003140735
Q4VC31	Protein MIX23	0.310472607	0.000441663
P35443	Thrombospondin-4	0.310797049	0.001832513
O15533	Tapasin	0.310937105	0.009870902
P15144	Aminopeptidase N	0.311360264	5.82583E-06
Q96AG4	Leucine-rich repeat-containing protein 59	0.312285603	0.004937639
Q96NZ9	Proline-rich acidic protein 1	0.313568583	0.000315946
Q92520	Protein FAM3C	0.314457978	0.001509264
O14791	Apolipoprotein L1	0.31498559	2.11334E-09
P05155	Plasma protease C1 inhibitor	0.31519058	4.26105E-06
P00533	Epidermal growth factor receptor	0.315570018	2.94888E-05
O14744	Protein arginine N-methyltransferase 5	0.315656922	0.000133286
P16671	Platelet glycoprotein 4	0.317530012	0.000236237
P05386	Large ribosomal subunit protein P1	0.318062364	0.010899896
P11279	Lysosome-associated membrane glycoprotein 1	0.318444815	7.43861E-05

O95202	Mitochondrial proton/calcium exchanger protein	0.318445837	0.013908466
P26447	Protein S100-A4 (Calvasculin)	0.318995216	0.000209414
P13804	Electron transfer flavoprotein subunit alpha, mitochondrial (Alpha-ETF)	0.319016628	0.000326821
Q9BX68	Adenosine 5'-monophosphoramidase HINT2	0.319394307	0.000695334
P08697	Alpha-2-antiplasmin	0.320438638	4.8655E-10
P30049	ATP synthase F(1) complex subunit delta, mitochondrial	0.32045449	0.000565508
Q6P2E9	Enhancer of mRNA-decapping protein 4	0.322342161	0.002264109
P10643	Complement component C7	0.323745238	3.76724E-07
O43567	E3 ubiquitin-protein ligase RNF13 (RING finger protein 13)	0.324461402	0.011992439
P35555	Fibrillin-1	0.325512164	0.002147422
P16109	P-selectin (CD62 antigen-like family member P)	0.326023894	4.06817E-06
Q8N149	Leukocyte immunoglobulin-like receptor subfamily A member 2	0.327165729	0.015359556
O75298	Reticulon-2 (NSP-like protein 1)	0.327920858	0.016452649
P17936	Insulin-like growth factor-binding protein 3	0.328116153	5.05621E-10
P07988	Pulmonary surfactant-associated protein B	0.32863317	0.008234968
A2NJV5	Immunoglobulin kappa variable 2-29	0.328876296	0.015163926
O75954	Tetraspanin-9	0.328904858	0.005551645
P00747	Plasminogen	0.329796641	8.78159E-08
P04430	Immunoglobulin kappa variable 1-16	0.330451875	7.43861E-05
P27824	Calnexin	0.330452049	0.000258347
P19256	Lymphocyte function-associated antigen 3	0.330849264	0.029953717
Q9NVK5	FGFR1 oncogene partner 2	0.332053394	0.012634219
P02745	Complement C1q subcomponent subunit A	0.332316816	0.004157062
P02743	Serum amyloid P-component	0.332880341	0.000709866
A0A0C4DH35	Probable non-functional immunoglobulin heavy variable 3-35	0.333834023	0.001382134
Q99941	Cyclic AMP-dependent transcription factor ATF-6 beta	0.334473286	0.002726669
P09769	Tyrosine-protein kinase Fgr	0.33484421	0.015096232
Q08379	Golgin subfamily A member 2	0.334863966	0.000450066
Q16663	C-C motif chemokine 15	0.335283319	0.010769455
Q9NZ43	Vesicle transport protein USE1	0.33543013	0.003479178
P22105	Tenascin-X	0.335842383	0.015322952
Q04756	Hepatocyte growth factor activator serine protease	0.337450614	7.62676E-07

Q6P3X3	Tetratricopeptide repeat protein 27	0.3376995<br>8	0.0022186<br>61
P48740	Mannan-binding lectin serine protease 1	0.3378256<br>27	0.0002661<br>97
P07204	Thrombomodulin	0.3385517<br>72	0.0002979<br>14
O75340	Programmed cell death protein 6	0.3389152<br>76	7.55952E-<br>05
P09172	Dopamine beta-hydroxylase	0.3395102<br>65	0.0005948<br>28
A0A0B4J1U3	Immunoglobulin lambda variable 1-36	0.3396607<br>85	0.0023823<br>82
Q8NBJ4	Golgi membrane protein 1	0.3399842<br>22	0.0002248<br>03
P35613	Basigin (Extracellular matrix metalloproteinase inducer)	0.3403080<br>73	2.38777E-<br>05
Q8N128	Protein FAM177A1	0.3408305<br>06	0.0001307<br>25
Q9BVK6	Transmembrane emp24 domain-containing protein 9	0.3419689<br>24	0.0014523<br>74
Q15828	Cystatin-M	0.3420988<br>96	4.25521E-<br>05
Q9NRX2	Large ribosomal subunit protein bL17m	0.3422560<br>24	0.0039413<br>26
P17813	Endoglin (CD antigen CD105)	0.3422865<br>73	1.28515E-<br>07
Q9HCU0	Endosialin (Tumour endothelial marker 1, CD antigen CD248)	0.3427383<br>3	0.0003790<br>14
P42892	Endothelin-converting enzyme 1	0.3432584<br>35	0.0012115<br>32
Q5T447	E3 ubiquitin-protein ligase HECTD3	0.3442191<br>02	0.0038480<br>56
O14786	Neuropilin-1	0.3449456<br>18	1.31489E-<br>05
Q9HD42	Charged multivesicular body protein 1a	0.3454403<br>14	0.0326762<br>07
Q9NS62	Thrombospondin type-1 domain-containing protein 1	0.3456912<br>23	0.0121729<br>62
Q9NVS9	Pyridoxine-5'-phosphate oxidase	0.3461456<br>97	0.0004461<br>26
Q14118	Dystroglycan 1	0.3465407<br>39	4.51564E-<br>06
P04211	Immunoglobulin lambda variable 7-43	0.3481614<br>06	0.0223198<br>43
Q92499	ATP-dependent RNA helicase DDX1	0.3487144<br>68	0.0140196<br>44
P04843	Dolichyl-diphosphooligosaccharide–protein glycosyltransferase subunit 1 (Ribophorin-1)	0.3493878<br>18	0.0005933<br>63
P02675	Fibrinogen beta chain	0.3494386<br>76	0.0001612<br>77
P43251	Biotinidase	0.3495191<br>8	7.51306E-<br>06
P13671	Complement component C6	0.3498419<br>73	2.8847E-<br>07
Q15113	Procollagen C-endopeptidase enhancer 1	0.3501316<br>99	0.0001706<br>39
Q6UY14	ADAMTS-like protein 4	0.3505486<br>05	0.0003836<br>79
P80303	Nucleobindin-2	0.3518384<br>14	0.0012390<br>5
P11021	Endoplasmic reticulum chaperone BiP	0.3521449<br>63	3.49813E-<br>07
Q6NW29	RWD domain-containing protein 4	0.3528376<br>71	0.0221859<br>5

Q9P000	COMM domain-containing protein 9	0.353292722	0.001064397
P35542	Serum amyloid A-4 protein	0.35408102	0.000650842
P61201	COP9 signalosome complex subunit 2	0.35440236	0.002036786
Q9Y6G5	COMM domain-containing protein 10	0.354734795	0.002364514
A0A0A0MT89	Immunoglobulin kappa joining 1	0.355266562	0.039894068
P01833	Polymeric immunoglobulin receptor	0.355395288	0.007251145
Q9H8J5	MANSC domain-containing protein 1	0.355784796	8.27016E-05
Q07021	Complement component 1 Q subcomponent-binding protein, mitochondrial (Hyaluronan-binding protein 1)	0.356323882	0.000709866
O75636	Ficolin-3	0.356860114	4.81241E-05
P52943	Cysteine-rich protein 2	0.35706142	9.86096E-06
P07998	Ribonuclease pancreatic	0.358947615	0.000997459
P14543	Nidogen-1	0.360586796	0.000313069
P24592	Insulin-like growth factor-binding protein 6	0.361297982	6.41401E-05
P02760	Protein AMBP	0.361770815	2.31418E-10
P15260	Interferon gamma receptor 1	0.362122666	0.039898011
P61278	Somatostatin	0.362283045	0.006802455
Q92930	Ras-related protein Rab-8B	0.363240901	0.011288304
P01877	Immunoglobulin heavy constant alpha 2	0.363482687	0.004368678
P61601	Neurocalcin-delta	0.363548183	0.003599564
Q6EMK4	Vasorin (Protein slit-like 2)	0.364405665	2.01369E-11
P01717	Immunoglobulin lambda variable 3-25	0.365722604	0.002724657
O43752	Syntaxin-6	0.365738255	0.012731879
P50502	Hsc70-interacting protein	0.367008411	0.000104588
P02679	Fibrinogen gamma chain	0.367692192	0.001382134
P23142	Fibulin-1 (FIBL-1)	0.367913725	0.026182043
Q06210	Glutamine–fructose-6-phosphate aminotransferase 1	0.368689783	0.007356704
Q96DU3	SLAM family member 6 (NK-T-B-antigen)	0.369056298	0.014312822
Q9HC35	Echinoderm microtubule-associated protein-like 4	0.369317883	0.009286141
Q9Y5M8	Signal recognition particle receptor subunit beta	0.370165349	0.031926257
A0A075B6H9	Immunoglobulin lambda variable 4-69	0.371648986	0.012791294
P05156	Complement factor I	0.372172352	3.27785E-09
Q9NYL4	Peptidyl-prolyl cis-trans isomerase FKBP11	0.372203882	0.003399859

P06756	Integrin alpha-V (Vitronectin receptor, CD antigen CD51)	0.372737087	0.027139448
Q0VD83	Apolipoprotein B receptor	0.372925151	0.00363142
Q13137	Calcium-binding and coiled-coil domain-containing protein 2 (Nuclear dot protein 52)	0.373131681	0.019296907
Q9UJJ9	N-acetylglucosamine-1-phosphotransferase subunit gamma	0.37379247	0.000442793
P39060	Collagen alpha-1(XVIII) chain	0.374256848	0.001089361
Q8NBP7	Proprotein convertase subtilisin/kexin type 9 (Neural apoptosis-regulated convertase 1)	0.374876278	0.007077261
P01011	Alpha-1-antichymotrypsin	0.374952693	3.88424E-06
P20645	Cation-dependent mannose-6-phosphate receptor	0.37512141	0.022976746
A0A087WW87;P01614	Immunoglobulin kappa variable 2-40	0.375425878	0.002724657
P30048	Thioredoxin-dependent peroxide reductase, mitochondrial (Antioxidant protein 1)	0.375798332	0.004243541
Q9Y224	RNA transcription, translation and transport factor protein	0.37701262	0.002716272
Q8NC42	E3 ubiquitin-protein ligase RNF149	0.377143095	0.004705674
Q8N183	NADH dehydrogenase [ubiquinone] 1 alpha subcomplex assembly factor 2	0.37715495	0.011305173
P07996	Thrombospondin-1	0.377301333	0.003336973
P09871	Complement C1s subcomponent	0.377450039	0.001446851
O75531	Barrier-to-autointegration factor	0.378128816	0.003769767
Q96BS2	Calcineurin B homologous protein 3	0.378390587	0.000156593
O00264	Membrane-associated progesterone receptor component 1	0.379266701	0.008539446
Q92882	Osteoclast-stimulating factor 1	0.379516113	0.002048088
Q96F46	Interleukin-17 receptor A	0.380372319	0.007076626
Q8WZA0	Protein LZIC	0.380944511	0.000880524
P05067	Amyloid-beta precursor protein	0.381685419	0.010892675
Q96BZ4	5'-3' exonuclease PLD4 (Phospholipase D4)	0.381920565	0.005015982
P13497	Bone morphogenetic protein 1	0.383734361	0.001139043
P02787	Serotransferrin	0.38414412	0.000122996
P98160	Basement membrane-specific heparan sulfate proteoglycan core protein (Perlecan)	0.385142689	0.014118175
Q9GZX9	Twisted gastrulation protein homolog 1	0.38712833	0.041440407
O14960	Leukocyte cell-derived chemotaxin-2	0.387842321	0.002070282
P61604	10 kDa heat shock protein, mitochondrial	0.388599788	7.55751E-05
Q14624	Inter-alpha-trypsin inhibitor heavy chain H4	0.388650981	4.69252E-08
Q9NNW7	Thioredoxin reductase 2, mitochondrial	0.389274027	0.015868205
Q99627	COP9 signalosome complex subunit 8	0.389675468	0.006743319

P08195	Amino acid transporter heavy chain SLC3A2	0.389704658	1.70757E-06
O95197	Reticulon-3	0.389787817	0.007781628
P00742	Coagulation factor X	0.390997945	2.98058E-08
Q9Y4L1	Hypoxia up-regulated protein 1	0.391586751	0.00123905
O75116	Rho-associated protein kinase 2	0.391964646	0.004025564
Q9BWP8	Collectin-11	0.392373388	0.002572431
Q9BRG1	Vacuolar protein-sorting-associated protein 25	0.393197359	0.006660575
Q8NBF2	NHL repeat-containing protein 2	0.393517231	0.009714933
P22059	Oxysterol-binding protein 1	0.395178035	0.020386035
Q9Y6C2	EMILIN-1	0.395703481	0.008368921
P13073	Cytochrome c oxidase subunit 4 isoform 1, mitochondrial	0.396096748	0.040330227
P05154	Plasma serine protease inhibitor (Plasminogen activator inhibitor 3)	0.396101565	8.06602E-06
Q3LXA3	Triokinase/FMN cyclase	0.396480517	0.047250025
Q8WWI5	Choline transporter-like protein 1 (CD antigen CD92)	0.396687237	4.85395E-05
P06312	Immunoglobulin kappa variable 4-1	0.39693233	0.000285244
Q9Y251	Heparanase	0.397546942	0.0150169 9
O95373	Importin-7	0.39818318	0.012738397
P02654	Apolipoprotein C-I	0.398512686	3.27939E-05
Q53HC0	Coiled-coil domain-containing protein 92 (Limkain beta-2)	0.401080006	0.011523516
Q9UKU6	Thyrotropin-releasing hormone-degrading ectoenzyme	0.402088459	0.017139335
O00461	Golgi integral membrane protein 4	0.40246521	0.006855276
P49257	Protein ERGIC-53	0.404090827	0.032612399
O95497	Pantetheinase	0.404096899	0.019813296
Q6P4E1	Protein GOLM2	0.404970972	0.016889517
P08582	Melanotransferrin	0.405528169	0.017913391
P01597;P04432	Immunoglobulin kappa variable 1-39	0.405613323	0.015274928
P36980	Complement factor H-related protein 2	0.406014156	0.000178174
Q96SB3	Neurabin-2	0.406535277	0.016206125
Q9Y696	Chloride intracellular channel protein 4 (Glutaredoxin-like oxidoreductase CLIC4)	0.40700795	0.000316474
Q96FN4	Copine-2	0.407354274	0.008146985
Q9NYL9	Tropomodulin-3	0.40748762	0.000474573
P20827	Ephrin-A1	0.40796264	0.004202287

Q99417	c-Myc-binding protein	0.408870314	0.044721772
Q9UIA9	Exportin-7	0.409431985	0.043277133
P38117	Electron transfer flavoprotein subunit beta	0.410584502	0.023565328
Q6ZMP0	Thrombospondin type-1 domain-containing protein 4 (ADAMTS-like protein 6)	0.410705969	0.026127992
Q12805	EGF-containing fibulin-like extracellular matrix protein 1 (Fibulin-3)	0.411224085	0.038305336
Q13464	Rho-associated protein kinase 1	0.412189108	0.00386856
P08571	Monocyte differentiation antigen CD14	0.416969032	2.16579E-08
P01859	Immunoglobulin heavy constant gamma 2	0.417647884	0.000115197
P20774	Mimecan (Osteoglycin, Osteoinductive factor)	0.418600165	0.000669861
P55145	Mesencephalic astrocyte-derived neurotrophic factor (Arginine-rich protein)	0.419488537	0.010985737
Q8N5N7	Large ribosomal subunit protein mL50	0.420021192	0.045062746
Q9Y2A7	Nck-associated protein 1	0.420274047	0.024198191
P51970	NADH dehydrogenase [ubiquinone] 1 alpha subcomplex subunit 8	0.420731295	0.013375144
Q7Z4G1	COMM domain-containing protein 6	0.421168876	0.031731167
P04196	Histidine-rich glycoprotein	0.421710406	8.36467E-05
Q9NP80	Calcium-independent phospholipase A2-gamma	0.422067815	0.028433849
P13688	Cell adhesion molecule CEACAM1	0.422373641	0.007234472
Q9BQS8	FYVE and coiled-coil domain-containing protein 1	0.422380377	0.004976963
L0R819	ASNSD1 upstream open reading frame protein	0.424533413	0.013601273
O94989	Rho guanine nucleotide exchange factor 15	0.424734211	0.043277133
Q01968	Inositol polyphosphate 5-phosphatase OCRL	0.426477021	0.001967576
Q7Z3B1	Neuronal growth regulator 1	0.428322601	1.32705E-05
P07237	Protein disulfide-isomerase	0.429972378	2.83611E-05
P01601	Immunoglobulin kappa variable 1D-16	0.430494966	0.044932429
P46939	Utrophin (Dystrophin-related protein 1, DRP-1)	0.430852269	0.001990883
P07358	Complement component C8 beta chain	0.432433141	4.92473E-06
Q9BUN1	Protein MENT	0.43391088	0.002265231
P23219	Prostaglandin G/H synthase 1 (Cyclooxygenase-1)	0.43450574	0.044798698
A1L0T0	2-hydroxyacyl-CoA lyase 2	0.434792006	0.002763828
Q12841	Follistatin-related protein 1	0.436000427	0.002724657
O75976	Carboxypeptidase D	0.436761477	0.02370942
P12111	Collagen alpha-3(VI) chain	0.437342736	0.020552814

Q14766	Latent-transforming growth factor beta-binding protein 1 (TGF-beta1-BP-1)	0.437518504	0.013300177
P50148	Guanine nucleotide-binding protein G(q) subunit alpha	0.43773523	0.031654971
A0A0C4DH24	Immunoglobulin kappa variable 6-21	0.438418873	0.017342253
P49238	CX3C chemokine receptor 1	0.438629072	0.037411584
O95816	BAG family molecular chaperone regulator 2	0.438907382	0.023565328
P21796	Non-selective voltage-gated ion channel VDAC1	0.439005216	0.002117041
Q96PD5	N-acetylmuramoyl-L-alanine amidase	0.440277242	2.86537E-05
P27105	Stomatin	0.441624102	0.00857979
O60826	Coiled-coil domain-containing protein 22	0.442679565	0.023497821
P80108	Phosphatidylinositol-glycan-specific phospholipase D	0.442831827	9.84146E-05
Q9BSF0	Small membrane A-kinase anchor protein	0.443365698	0.015274928
P12830	Cadherin-1	0.444223409	0.000563624
Q6PJW8	Consortin	0.444458224	0.025341136
P05186	Alkaline phosphatase, tissue-nonspecific isozyme	0.447510924	0.011844744
Q8NF50	Dedicator of cytokinesis protein 8	0.448734754	0.013385599
O14498	Immunoglobulin superfamily containing leucine-rich repeat protein	0.449783023	0.02157879
P59768	Guanine nucleotide-binding protein G(I)/G(S)/G(O) subunit gamma-2 (G gamma-I)	0.450153553	0.008794194
Q9NZM3	Intersectin-2	0.450355897	0.017459022
A0A075B6K5	Immunoglobulin lambda variable 3-9	0.451935131	0.000153119
Q13308	Inactive tyrosine-protein kinase 7	0.452932209	0.000667843
Q16270	Insulin-like growth factor-binding protein 7	0.453437633	0.000112565
Q9BSJ2	Gamma-tubulin complex component 2	0.454640537	0.015163926
P0DPI2	Glutamine amidotransferase-like class 1 domain-containing protein 3, mitochondrial	0.455359447	0.012675434
Q10588	ADP-ribosyl cyclase/cyclic ADP-ribose hydrolase 2	0.456301838	0.00363142
Q9Y210	Short transient receptor potential channel 6	0.456659084	0.008704785
Q96RU3	Formin-binding protein 1	0.457554128	0.014247931
O75208	Ubiquinone biosynthesis protein COQ9, mitochondrial	0.45808273	0.004352095
P15814	Immunoglobulin lambda-like polypeptide 1	0.458434554	0.048871438
Q13045	Protein flightless-1 homolog	0.458600867	0.024465155
Q9H0X4	Protein FAM234A	0.459340328	0.030684983
A0A0G2JRQ6	Ig-like domain-containing protein	0.459841418	0.024406698
Q6ZRY4	RNA-binding protein with multiple splicing 2	0.460211764	0.036121654

Q8TDL5	BPI fold-containing family B member 1	0.460461844	0.043194604
Q09666	Neuroblast differentiation-associated protein AHNAK	0.460625886	0.000422432
P01871	Immunoglobulin heavy constant mu	0.462915698	0.005007195
P49908	Selenoprotein P	0.463128933	2.14237E-05
Q13813	Spectrin alpha chain, non-erythrocytic 1	0.466805175	0.022976746
Q6UWL2	Sushi domain-containing protein 1	0.468775435	0.012830525
Q9NVA2	Septin-11	0.472203583	0.026208522
P55290	Cadherin-13	0.472360725	2.74849E-05
P55011	Solute carrier family 12 member 2	0.473740718	0.031926257
P00738	Haptoglobin (Zonulin)	0.473969336	2.38777E-05
P02746	Complement C1q subcomponent subunit B	0.47427647	0.016502521
Q6UX71	Plexin domain-containing protein 2	0.474286614	0.000167057
P22692	Insulin-like growth factor-binding protein 4	0.475038631	0.006080503
Q01469	Fatty acid-binding protein 5	0.475492849	0.004792249
Q9Y394	Dehydrogenase/reductase SDR family member 7	0.475783711	0.030959301
P07602	Prosaposin (Proactivator polypeptide)	0.476095682	0.010994737
P02747	Complement C1q subcomponent subunit C	0.477024659	0.007748006
A0A075B6K4	Immunoglobulin lambda variable 3-10	0.477253591	0.025541787
A0A0C4DH68	Immunoglobulin kappa variable 2-24	0.478910262	0.005890279
P00492	Hypoxanthine-guanine phosphoribosyltransferase	0.479136594	0.007076626
A0A0C4DH31	Immunoglobulin heavy variable 1-18	0.480919185	0.006971399
P15169	Carboxypeptidase N catalytic chain	0.481087135	0.002910627
P09493	Tropomyosin alpha-1 chain	0.483752672	0.006113615
Q9UM47	Neurogenic locus notch homolog protein 3 (Notch 3)	0.48389171	0.005598666
P06727	Apolipoprotein A-IV	0.485076641	0.00091215
P18054	Polyunsaturated fatty acid lipoxygenase ALOX12	0.485372981	0.012524692
P01593;P01594	Immunoglobulin kappa variable 1D-33	0.488917385	0.003178715
P01599	Immunoglobulin kappa variable 1-17	0.49107631	0.002048088
A1L4H1	Soluble scavenger receptor cysteine-rich domain-containing protein SSC5D	0.492075178	0.041824118
P35241	Radixin	0.49306147	0.00673987
Q0VDF9	Heat shock 70 kDa protein 14	0.495795668	0.040994731
Q4V328	GRIP1-associated protein 1	0.497832695	0.028979887

O14773	Tripeptidyl-peptidase 1	0.498886755	0.040979458

Table S5: Results of functional enrichment analysis for the upregulated DEPs of the SSc-ILD vs. CTRL contrast showing top 20 significant enriched terms (or all significant results if less than 20 were identified) in all analysed pathway databases.

Enrichment FDR	Number of genes	Fold Enrichment	Pathway name
1.56E-06	15	5.785594327	Path:hsa05202 Transcriptional misregulation in cancer
1.86E-07	21	4.776824034	Path:hsa03010 Ribosome
1.16E-05	17	4.371337936	Path:hsa05034 Alcoholism
7.05E-05	19	3.439870369	Path:hsa04613 Neutrophil extracellular trap formation
0.002001298	14	3.356687159	Path:hsa05322 Systemic lupus erythematosus
6.43E-05	23	3.045352636	Path:hsa05171 Coronavirus disease-COVID-19
0.00101753	5	17.74248927	GO:0031340 Pos. reg. of vesicle fusion
0.004321761	5	12.67320662	GO:0031338 Reg. of vesicle fusion
0.003153071	7	8.871244635	GO:0042273 Ribosomal large subunit biogenesis
0.003564467	10	5.544527897	GO:0050832 Defence response to fungus
0.005236508	10	5.069282649	GO:0009620 Response to fungus
0.003153071	12	4.83886071	GO:0006334 Nucleosome assembly
0.003153071	12	4.731330472	GO:0034728 Nucleosome organization
0.003698439	12	4.529997261	GO:0016072 rRNA metabolic proc.
0.039359773	9	4.202168511	GO:0006364 rRNA processing
0.005842587	12	4.174703358	GO:0065004 Protein–DNA complex assembly
0.011803961	11	4.152497489	GO:0040029 Epigenetic reg. of gene expression
0.008135895	12	4.017167382	GO:0071824 Protein–DNA complex organization
0.003931549	14	3.881169528	GO:0042254 Ribosome biogenesis
0.039359773	10	3.857062885	GO:0031640 Killing of cells of another organism
0.039359773	10	3.857062885	GO:0141061 Disruption of cell in another organism
0.042670709	10	3.774997717	GO:0141060 Disruption of anatomical structure in another organism
0.020180129	12	3.608641886	GO:0032200 Telomere organization
0.00101753	22	3.336194564	GO:0002181 Cytoplasmic translation
0.016434336	17	2.872593501	GO:0022613 Ribonucleoprotein complex biogenesis
3.01E-06	14	6.05841097	GO:0022625 Cytosolic large ribosomal subunit
0.03350761	5	5.544527897	GO:0005834 Heterotrimeric g-protein complex
0.039509753	5	5.218379197	GO:1905360 Gtpase complex
0.000145524	14	4.357804382	GO:0015934 Large ribosomal subunit
0.000893596	12	4.258197425	GO:0000786 Nucleosome
1.85E-06	21	4.234003121	GO:0022626 Cytosolic ribosome

3.61E-06	21	3.922023944	GO:0044391 Ribosomal subunit
0.019788547	9	3.629145533	GO:0070821 Tertiary granule membrane
0.005442063	12	3.548497854	GO:0032993 Protein–DNA complex
3.61E-06	24	3.490325758	GO:0005840 Ribosome
1.85E-06	34	2.942656757	GO:1990904 Ribonucleoprotein complex
0.019825895	12	2.916573579	GO:0035578 Azurophil granule lumen
0.03350761	26	1.852629402	GO:0034774 Secretory granule lumen
0.03350761	26	1.845218884	GO:0060205 Cytoplasmic vesicle lumen
0.033827443	26	1.837867414	GO:0031983 Vesicle lumen
0.008225999	49	1.655965665	GO:0030141 Secretory granule
0.024437323	50	1.532166604	GO:0099503 Secretory vesicle
0.02015753	69	1.420222459	GO:0005654 Nucleoplasm
0.017812054	99	1.321675273	GO:0070062 Extracellular exosome
0.017812054	99	1.315735159	GO:1903561 Extracellular vesicle
0.000743541	12	4.628475462	GO:0030527 Structural constituent of chromatin
3.17E-06	21	4.33246831	GO:0003735 Structural constituent of ribosome
0.000743541	32	2.280159264	GO:0045296 Cadherin binding
0.000417841	44	2.038301639	GO:0005198 Structural molecule activity
0.000325572	67	1.737933891	GO:0003676 Nucleic acid binding
0.035352151	48	1.616014203	GO:0003723 RNA binding

Table S6: Results of functional enrichment analysis for the downregulated DEPs of the SSc-ILD vs. CTRL contrast showing top 20 significant enriched terms (or all significant results if less than 20 were identified) in all analysed pathway databases.

Enrichment FDR	Number of genes	Fold Enrichment	Pathway name
5.69E-22	35	7.916073969	Path:hsa04610 Complement and coagulation cascades
0.003438385	10	4.404432133	Path:hsa05133 Pertussis
0.001121248	14	3.779286927	Path:hsa05150 Staphylococcus aureus infection
0.005321224	20	2.498036135	Path:hsa05171 Coronavirus disease-COVID-19
0.000537961	5	13.94736842	GO:2000427 Pos. reg. of apoptotic cell clearance
0.00309775	5	10.46052632	GO:2000425 Reg. of apoptotic cell clearance
0.048515399	3	10.04210526	GO:0002604 Reg. of dendritic cell antigen processing and presentation
0.048515399	3	10.04210526	GO:0030049 Muscle filament sliding
0.048515399	3	10.04210526	GO:0032490 Detection of molecule of bacterial origin
0.048515399	3	10.04210526	GO:0042308 Neg. reg. of protein import into nucleus
0.000113749	8	8.926315789	GO:0006957 Complement activation alternative pathway
2.08E-09	16	8.638370119	GO:0006958 Complement activation classical pathway

0.008045785	5	8.368421053	GO:0010575 Pos. reg. of vascular endothelial growth factor production
0.024349464	4	8.368421053	GO:0048265 Response to pain
0.001075475	7	7.810526316	GO:0003044 Reg. of systemic arterial blood pressure mediated by a chemical signal
0.001075475	7	7.810526316	GO:0008228 Opsonization
2.34E-06	12	7.724696356	GO:0006953 Acute-phase response
3.21E-09	17	7.689900427	GO:0002455 Humoral immune response mediated by circulating immunoglobulin
0.011809794	5	7.607655502	GO:0097421 Liver regeneration
0.000518947	8	7.438596491	GO:0006910 Phagocytosis recognition
0.036359581	4	7.438596491	GO:0045777 Pos. reg. of blood pressure
0.017170335	5	6.973684211	GO:0045916 Neg. reg. of complement activation
0.002623191	7	6.891640867	GO:0030449 Reg. of complement activation
1.21E-09	21	6.759109312	GO:0006956 Complement activation
0.003483157	3	16.73684211	GO:0070938 Contractile ring
0.017757775	4	7.438596491	GO:0034707 Chloride channel complex
6.20E-19	37	6.384156267	GO:0072562 Blood microparticle
0.013931255	5	5.977443609	GO:0031092 Platelet alpha granule membrane
2.55E-10	24	5.149797571	GO:0031091 Platelet alpha granule
1.16E-07	18	5.106155219	GO:0031093 Platelet alpha granule lumen
0.033754927	5	4.922600619	GO:0046930 Pore complex
0.000244627	11	4.720647773	GO:0005604 Basement membrane
0.000572066	13	3.687778769	GO:0005793 Endoplasmic reticulum–Golgi intermediate compartment
7.40E-10	34	3.578947368	GO:0005788 Endoplasmic reticulum lumen
8.98E-17	58	3.529952153	GO:0031012 Extracellular matrix
8.98E-17	58	3.517162471	GO:0030312 External encapsulating structure
3.62E-14	50	3.486842105	GO:0062023 Collagen-containing extracellular matrix
0.04200438	9	2.896761134	GO:0005796 Golgi lumen
0.045352656	9	2.842105263	GO:0042383 Sarcolemma
0.000333253	25	2.490601504	GO:0009897 External side of plasma membrane
0.000235574	33	2.200461313	GO:0031983 Vesicle lumen
0.000450155	32	2.150919467	GO:0034774 Secretory granule lumen
0.000466833	32	2.142315789	GO:0060205 Cytoplasmic vesicle lumen
2.09E-05	45	2.097932854	GO:0009986 Cell surface
0.011232487	5	6.973684211	GO:0005254 Chloride channel activity
0.005682165	6	6.694736842	GO:0050431 Transforming growth factor beta binding
0.033831039	4	6.694736842	GO:0005244 Voltage-gated monoatomic ion channel activity
0.033831039	4	6.694736842	GO:0016805 Dipeptidase activity
0.033831039	4	6.694736842	GO:0022832 Voltage-gated channel activity
0.033831039	4	6.694736842	GO:0097493 Structural molecule activity conferring elasticity

0.007448182	6	6.276315789	GO:0005253 Monoatomic anion channel activity
0.007448182	6	6.276315789	GO:0015108 Chloride transmembrane transporter activity
0.046879982	4	6.086124402	GO:0005540 Hyaluronic acid binding
0.005682165	7	5.578947368	GO:0008509 Monoatomic anion transmembrane transporter activity
0.003157755	8	5.355789474	GO:0001848 Complement binding
0.020707117	6	5.021052632	GO:0015103 Inorganic anion transmembrane transporter activity
3.01E-10	27	4.859083192	GO:0030414 Peptidase inhibitor activity
9.13E-10	26	4.781954887	GO:0004866 Endopeptidase inhibitor activity
8.98E-06	16	4.781954887	GO:0004867 Serine-type endopeptidase inhibitor activity
3.01E-10	28	4.733652313	GO:0061135 Endopeptidase regulator activity
0.010708793	8	4.319185059	GO:0008235 Metalloexopeptidase activity
3.01E-10	31	4.287951283	GO:0061134 Peptidase regulator activity
3.65E-05	18	3.862348178	GO:0008236 Serine-type peptidase activity
2.25E-05	19	3.831325301	GO:0005201 Extracellular matrix structural constituent

Table S7: Results of functional enrichment analysis for the upregulated DEPs of the SSc w/o ILD vs. CTRL contrast showing top 20 significant enriched terms (or all significant results if less than 20 were identified) in all analysed pathway databases.

Enrichment FDR	Number of genes	Fold Enrichment	Pathway name
1.89E-06	25	3.55227882	Path:hsa03010 Ribosome
0.00224341	18	2.891246066	Path:hsa05034 Alcoholism
0.038925628	12	2.891246066	Path:hsa05202 Transcriptional misregulation in cancer
0.002099287	28	2.315873715	Path:hsa05171 Coronavirus disease-COVID-19
0.003718318	15	3.778332927	GO:0006334 Nucleosome assembly
0.002333439	17	3.694369973	GO:0065004 Protein–DNA complex assembly
0.004100274	15	3.694369973	GO:0034728 Nucleosome organization
0.002871241	17	3.554959786	GO:0071824 Protein–DNA complex organization
0.022797501	14	3.301351891	GO:0040029 Epigenetic reg. of gene expression
0.022362059	17	2.943951072	GO:0042254 Ribosome biogenesis
8.04E-05	31	2.936550492	GO:0002181 Cytoplasmic translation
0.022362059	46	1.807883178	GO:0006412 Translation
0.000840478	6	8.31233244	GO:0016281 Eukaryotic translation initiation factor 4f complex
0.000840478	6	8.31233244	GO:0034518 RNA cap binding complex
0.025321611	7	4.310098302	GO:0005839 Proteasome core complex
0.000103939	15	4.054796312	GO:0022625 Cytosolic large ribosomal subunit
0.00179899	12	3.799923401	GO:0022627 Cytosolic small ribosomal subunit
0.004089282	12	3.499929448	GO:0015935 Small ribosomal subunit
2.63E-06	27	3.400499634	GO:0022626 Cytosolic ribosome

5.49E-06	27	3.149936503	GO:0044391 Ribosomal subunit
0.004170419	14	3.103270777	GO:0000786 Nucleosome
0.004680648	15	2.916607874	GO:0015934 Large ribosomal subunit
3.60E-06	32	2.907045225	GO:0005840 Ribosome
0.026201117	14	2.586058981	GO:0032993 Protein–DNA complex
2.36E-05	42	2.270685935	GO:1990904 Ribonucleoprotein complex
3.91E-05	117	1.504320024	GO:0005654 Nucleoplasm
0.004845529	14	3.37312041	GO:0030527 Structural constituent of chromatin
2.46E-05	25	3.221834279	GO:0003735 Structural constituent of ribosome
0.002989632	43	1.913950709	GO:0045296 Cadherin binding
8.16E-07	106	1.71755797	GO:0003676 Nucleic acid binding
0.000618173	78	1.64038439	GO:0003723 RNA binding

Table S8: Results of functional enrichment analysis for the downregulated DEPs of the SSc w/o ILD vs. CTRL contrast showing top 20 significant enriched terms (or all significant results if less than 20 were identified) in all analysed pathway databases.

Enrichment FDR	Number of genes	Fold Enrichment	Pathway name
1.63E-23	45	5.636589504	Path:hsa04610 Complement and coagulation cascades
0.001666384	14	3.414916214	Path:hsa05133 Pertussis
0.000461777	20	2.990018805	Path:hsa05150 Staphylococcus aureus infection
0.009568189	29	2.005990228	Path:hsa05171 Coronavirus disease-COVID-19
0.044247887	4	7.415246637	GO:0032490 Detection of molecule of bacterial origin
2.93E-13	23	6.877043252	GO:0006958 Complement activation classical pathway
0.000160438	10	6.179372197	GO:0006957 Complement activation alternative pathway
5.41E-12	24	6.012362138	GO:0002455 Humoral immune response mediated by circulating immunoglobulin
0.044247887	5	5.793161435	GO:0098883 Synapse pruning
3.27E-15	32	5.704035874	GO:0006956 Complement activation
0.020258146	6	5.561434978	GO:0051918 Neg. reg. of fibrinolysis
0.020258146	6	5.561434978	GO:1903027 Reg. of opsonization
4.47E-06	15	5.347533632	GO:0006953 Acute-phase response
0.000792352	10	5.149476831	GO:0006910 Phagocytosis recognition
0.037515121	6	5.05584998	GO:0001867 Complement activation lectin pathway
0.00664213	8	4.943497758	GO:0008228 Opsonization
0.017950825	8	4.361909786	GO:0030449 Reg. of complement activation
0.039450474	7	4.325560538	GO:0003044 Reg. of systemic arterial blood pressure mediated by a chemical signal
0.039450474	7	4.325560538	GO:0051917 Reg. of fibrinolysis

0.002233262	11	4.248318386	GO:0042730 Fibrinolysis
0.017373175	9	3.972453555	GO:0030194 Pos. reg. of blood coagulation
0.017373175	9	3.972453555	GO:1900048 Pos. reg. of haemostasis
0.037515121	8	3.902761388	GO:0009593 Detection of chemical stimulus
0.010616702	10	3.862107623	GO:0050820 Pos. reg. of coagulation
0.0212927	3	9.269058296	GO:0070938 Contractile ring
2.19E-21	50	4.777865101	GO:0072562 Blood microparticle
0.015427331	6	4.634529148	GO:0031089 Platelet dense granule lumen
0.036587351	5	4.634529148	GO:0001750 Photoreceptor outer segment
0.036587351	5	4.634529148	GO:1905370 Serine-type endopeptidase complex
0.00402687	8	4.361909786	GO:0046930 Pore complex
0.021130638	7	3.816671063	GO:0042827 Platelet dense granule
0.000720247	13	3.544051701	GO:0005581 Collagen trimer
1.17E-05	21	3.299156343	GO:0031093 Platelet alpha granule lumen
1.65E-14	55	3.206278027	GO:0005788 Endoplasmic reticulum lumen
1.68E-20	87	2.932392988	GO:0031012 Extracellular matrix
1.68E-20	87	2.921768376	GO:0030312 External encapsulating structure
1.48E-16	74	2.857959641	GO:0062023 Collagen-containing extracellular matrix
3.48E-05	24	2.852017937	GO:0031091 Platelet alpha granule
0.010972839	12	2.852017937	GO:0005604 Basement membrane
0.007105771	15	2.623318386	GO:0042383 Sarcolemma
0.007443494	16	2.513642928	GO:0005793 Endoplasmic reticulum-Golgi intermediate compartment
0.016334533	14	2.495515695	GO:0005796 Golgi lumen
5.70E-05	39	2.151745676	GO:0009897 External side of plasma membrane
1.66E-06	72	1.85897548	GO:0009986 Cell surface
0.045498496	4	6.179372197	GO:0140828 Metal cation:monoatomic cation antiporter activity
0.001974648	7	5.898491643	GO:0005540 Hyaluronic acid binding
0.018794543	5	5.793161435	GO:0005501 Retinoid binding
0.018794543	5	5.793161435	GO:0019840 Isoprenoid binding
0.00033741	9	5.561434978	GO:0050431 Transforming growth factor beta binding
0.010258871	6	5.561434978	GO:0097493 Structural molecule activity conferring elasticity
0.012283292	7	4.634529148	GO:0030169 Low-density lipoprotein particle binding
0.000217248	12	4.449147982	GO:0001848 Complement binding
0.003198852	9	4.390606561	GO:0001846 Opsonin binding
0.016027018	8	3.902761388	GO:0071813 Lipoprotein particle binding
0.016027018	8	3.902761388	GO:0071814 Protein–lipid complex binding
2.49E-07	30	3.350262035	GO:0005201 Extracellular matrix structural constituent

5.05E-05	20	3.310377963	GO:0004867 Serine-type endopeptidase inhibitor activity
2.79E-06	30	2.990018805	GO:0030414 Peptidase inhibitor activity
2.72E-05	25	2.970852018	GO:0008236 Serine-type peptidase activity
5.36E-06	29	2.953875721	GO:0004866 Endopeptidase inhibitor activity
3.19E-05	25	2.933246296	GO:0017171 Serine hydrolase activity
0.000200974	22	2.872102571	GO:0004252 Serine-type endopeptidase activity
2.26E-06	34	2.813821268	GO:0005539 Glycosaminoglycan binding
8.37E-06	30	2.808805544	GO:0061135 Endopeptidase regulator activity

Supplementary table S9. Pairwise AUC comparisons (DeLong's Test) of the three biomarker candidate proteins. No statistically significant difference was found among them.

		**95% Confidence Interval**			
	**AUC Difference**	**Lower**	**Upper**	**z**	**p**
**SP-B vs. Cav-1**	-0.02812	-0.134	0.0775	-0.5220	0.602
**SP-B vs. Siglec-5**	-0.03125	-0.220	0.1571	-0.3252	0.745
**Cav-1 vs. Siglec-5**	-0.00313	-0.199	0.1929	-0.0313	0.975

Supplementary table S10. Correlation matrix of MAXLFQ, KNIME normalized data with %predicted values for diffusion capacity for CO (DLco), forced vital capacity (FVC), Goh score and immunosuppression (IS) (yes/no) for patients with SSc.

Correlation Matrix

		Siglec-5	Cav-1	SP-B	DLco	FVC	GOH	IS	SSc-ILD
**Siglec-5**	**Spearman's rho**	—							
	**df**	—							
	**p-value**	—							
**Cav-1**	**Spearman's rho**	0.453 **	—						
	**df**	41	—						
	**p-value**	0.002	—						
**SP-B**	**Spearman's rho**	0.159	0.417 **	—					
	**df**	41	41	—					
	**p-value**	0.309	0.005	—					
**DLco**	**Spearman's rho**	-0.356	-0.401	-0.484 *	—				
	**df**	21	21	21	—				
	**p-value**	0.095	0.058	0.019	—				
**FVC**	**Spearman's rho**	-0.543 **	-0.351	-0.358	0.509*	—			
	**df**	23	23	23	21	—			
	**p-value**	0.005	0.085	0.079	0.013	—			
**GOH**	**Spearman's rho**	0.694 ***	0.710 ***	0.639 ***	-0.509*	-0.620 ***	—		
	**df**	24	24	24	21	23	—		
	**p-value**	<.001	<.001	<.001	0.013	<.001	—		
**IS**	**Spearman's rho**	0.245	0.339	0.154	0.040	0.091	0.213	—	
	**df**	24	24	24	21	23	24	—	
	**p-value**	0.228	0.090	0.453	0.857	0.666	0.296	—	
**SSc-ILD**	**Spearman's rho**	0.677 ***	0.705 ***	0.635 ***	-0.483*	-0.618 ***	0.989 ***	0.197	—
	**df**	24	24	24	21	23	24	24	—
	**p-value**	<.001	<.001	<.001	0.019	<.001	<.001	0.335	—

Note. * p < .05, ** p < .01, *** p < .001, IS - immunosuppression

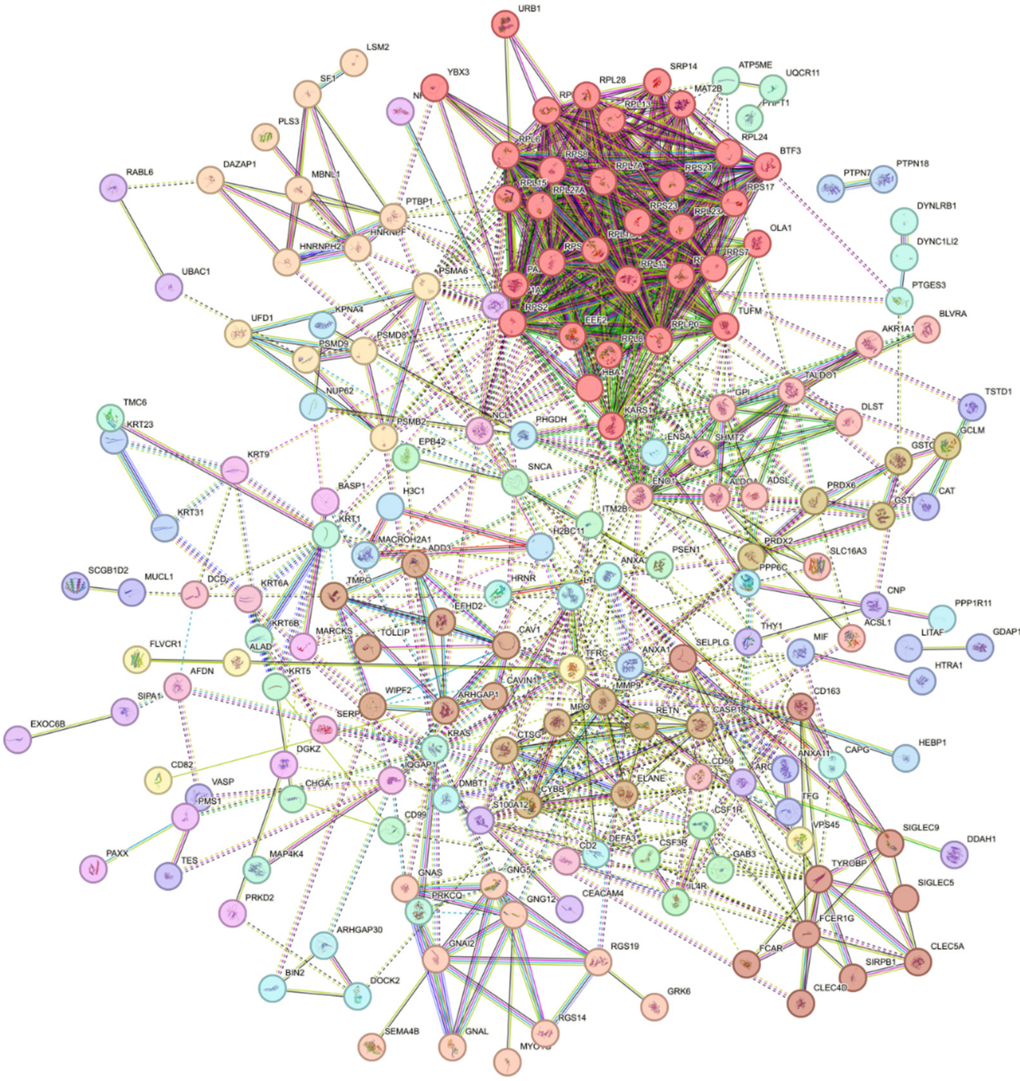

Figure S1: PPI network for the upregulated DEPs from the SSc-ILD vs. CTRL contrast (created using STRING v.12.0).

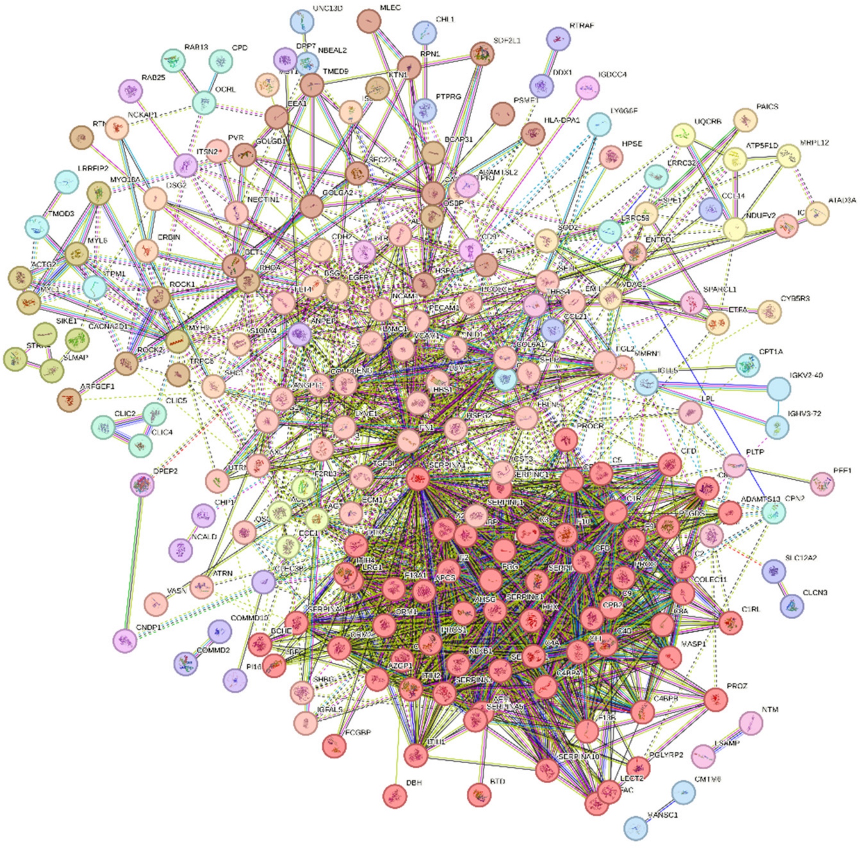

Figure S2: PPI network for the downregulated DEPs from the SSc-ILD vs. CTRL contrast (created using STRING v.12.0).

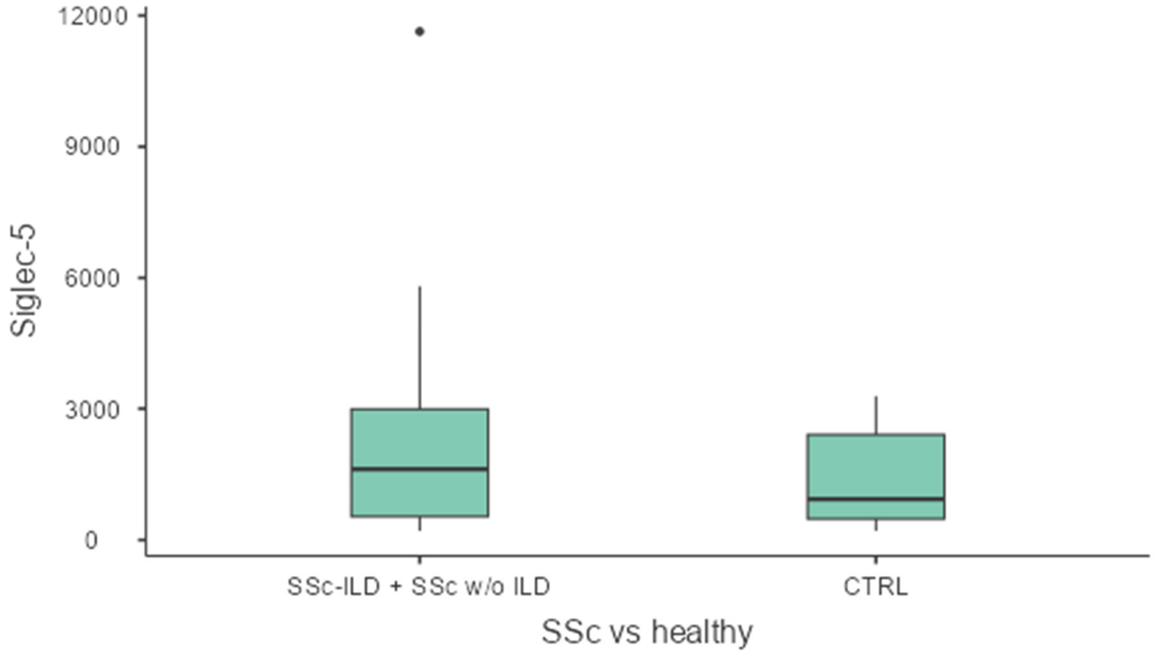

Figure S3. Box plot comparing the distribution of expression (MAXLFQ, KNIME normalized values) of the biomarker candidate protein Siglec-5 between all subjects with SSc (the SSc-ILD and SSc w/o ILD groups combined) and the control (CTRL) group.

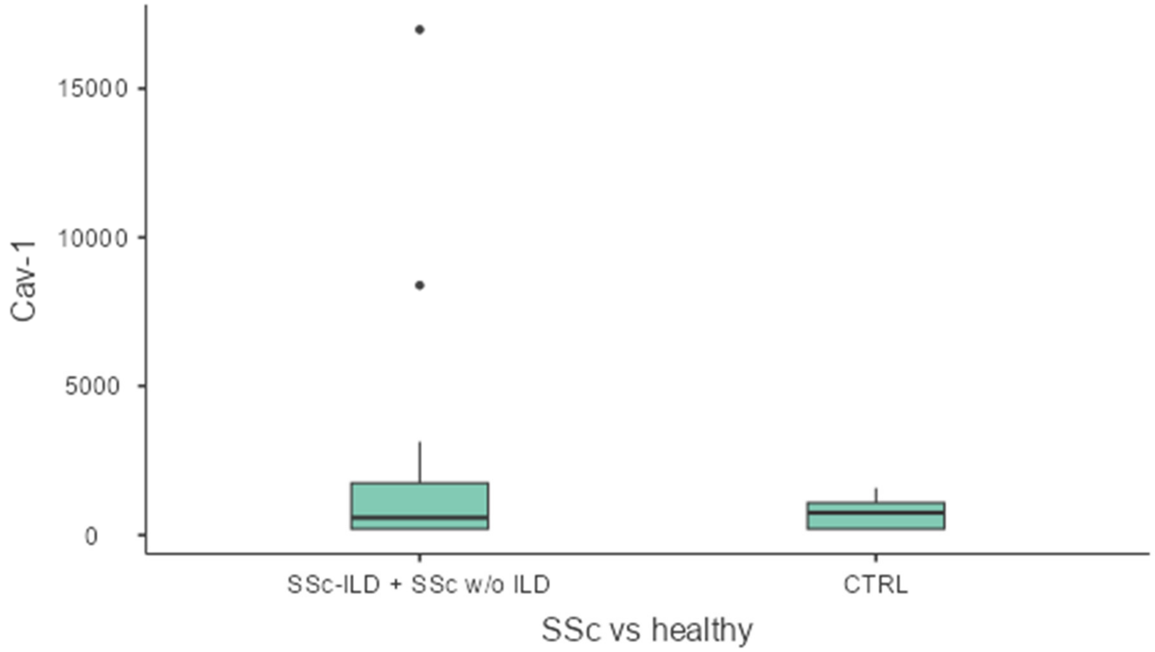

Figure S4. Box plot comparing distribution of expression (MAXLFQ, KNIME normalized values) of the biomarker candidate protein Cav-1 between all subjects with SSc (the SSc-ILD and SSc w/o ILD groups combined) and the control (CTRL) group.

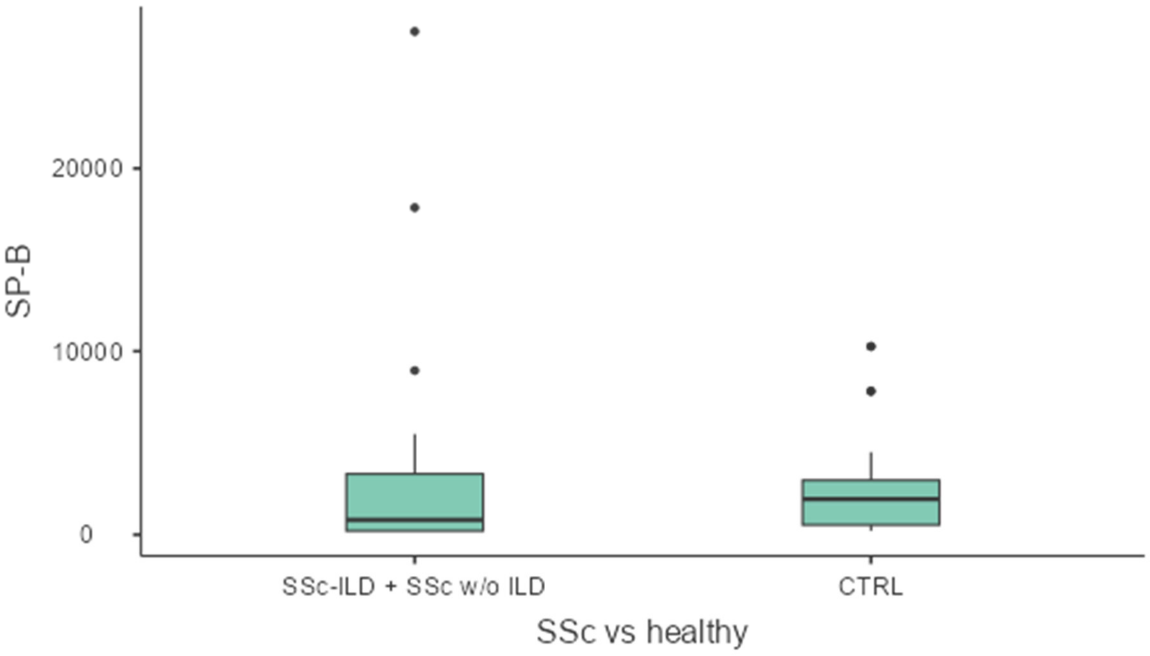

Figure S5. Box plot comparing distribution of expression (MAXLFQ, KNIME normalized values) of the biomarker candidate protein SP-B between all subjects with SSc (the SSc-ILD and SSc w/o ILD groups combined) and the control (CTRL) group.
